# AHA1 Regulates Aβ Production via Modulation of APP Abundance and γ‐Secretase Assembly

**DOI:** 10.1111/jnc.70544

**Published:** 2026-08-27

**Authors:** Arshad Ali Noorani, Sadequl Islam, Emma Filip, Kevin Catalfano, Shravan Paswan, Vaishnavi Nagarajan, Heather M. Wilkins, Brian S. J. Blagg, Kun Zou, Michael S. Wolfe

**Affiliations:** ^1^ Department of Medicinal Chemistry The University of Kansas Lawrence Kansas USA; ^2^ Department of Pharmacology & Immunology Medical University of South Carolina Charleston South Carolina USA; ^3^ Department of Neuro‐Oncology, Institute of Brain Science, Graduate School of Medical Sciences Nagoya City University Nagoya Japan; ^4^ Department of Chemistry and Biochemistry University of Notre Dame Notre Dame Indiana USA; ^5^ School of Natural Sciences (Biology) Northwest Missouri State University Maryville Missouri USA; ^6^ University of Kansas Alzheimer's Disease Research Center University of Kansas Medical Center Kansas Kansas USA

**Keywords:** activator of Hsp90 ATPase homolog 1 (AHA1), Alzheimer's disease (AD), amyloid β‐protein (Aβ), amyloid‐β precursor protein (APP), co‐chaperones, heat shock protein 90 (Hsp90), γ‐secretase

## Abstract

Deposition of amyloid β‐protein (Aβ) is a hallmark of Alzheimer's disease (AD), produced by γ‐secretase–mediated cleavage of amyloid precursor protein (APP). The 90‐kDa heat shock protein (Hsp90) co‐chaperone, activator of Hsp90 ATPase homolog 1 (AHA1), is known to promote the accumulation of toxic tau species; however, its effects on Aβ production remain unclear. Using cellular models—including human embryonic kidney 293 T (HEK293T) cells, stable HEK‐APP cells, presenilin 1/2 double‐knockout mouse embryonic fibroblasts (PS‐DKO MEFs), and Chinese hamster ovary (CHO) cells—in which AHA1 was experimentally manipulated, together with observational analyses of human iPSC‐derived neurons carrying an FAD‐linked APP mutation, we demonstrate that AHA1 regulates Aβ generation through two mechanisms: regulation of APP protein abundance through pathways that are at least partially independent of Hsp90 interaction and Hsp90‐dependent promotion of γ‐secretase assembly. Knockdown of endogenous AHA1 reduces Aβ production and decreases APP and γ‐secretase component levels, whereas AHA1 overexpression elevates Aβ generation and increases the expression of these proteins. The AHA1‐E67K mutant, which has impaired Hsp90 binding, lowers Aβ production and the levels of APP and γ‐secretase components compared with wild‐type AHA1. AHA1 associates with APP and APH1, an immature γ‐secretase component, indicating its role in APP proteolysis and Aβ production. Disruption of the AHA1/Hsp90 complex—through AHA1 knockdown, the E67K mutant, or a small‐molecule inhibitor—reduces γ‐secretase assembly. Notably, Familial AD mutations (APP‐C99‐I45F, PS1‐L286V) upregulate AHA1/Hsp90, elevating APP/APH1 and Aβ42 production. AHA1 knockdown rescues FAD‐linked Aβ42 overproduction in a mutant cell model, while AHA1 overexpression exacerbates PS1 mutant Aβ production. Collectively, these findings reveal that AHA1 regulates Aβ production by modulating APP abundance and γ‐secretase assembly, establishing AHA1 as a potential target for therapeutic intervention in AD.

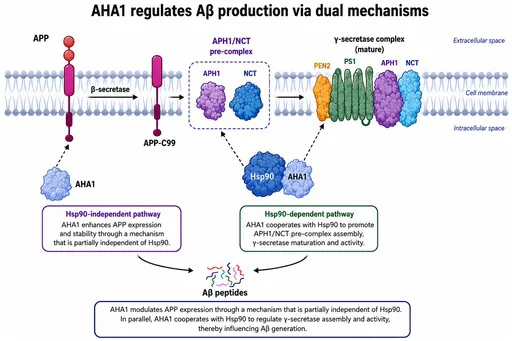

## Graphical abstract

AHA1 regulates Aβ production through dual mechanisms. AHA1 promotes APP abundance through mechanisms that are at least partially independent of Hsp90 interaction, while cooperating with Hsp90 to regulate APH1/NCT pre‐complex formation, γ‐secretase assembly, and γ‐secretase activity. These coordinated mechanisms contribute to increased Aβ generation and suggest that AHA1 is an important regulator of amyloidogenic processing in AD.

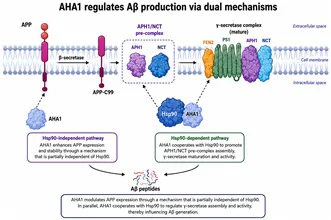

**Abbreviations**
AD: Alzheimer's disease
AHA1: activator of Hsp90 ATPase homolog 1
ANOVA: analysis of variance
APH1: anterior pharynx‐defective‐1
APP: amyloid precursor protein
BACE1: β‐site APP‐cleaving enzyme 1
BisTris: bis(2‐hydroxyethyl) aminotris (hydroxymethyl) methane
BN‐PAGE: blue‐native polyacrylamide gel electrophoresis
CHO: Chinese hamster ovary
EV: empty vector
FAD: Familial Alzheimer's disease
HEK: human embryonic kidney
HSP70: heat shock protein 70
HSP90: heat shock protein 90
IP: immunoprecipitation
NCT: nicastrin
NICD: notch intracellular domain
NT: non‐transfected
PEN‐2: presenilin enhancer‐2
PS: presenilin
PS1‐CTF: C‐terminal fragment of presenilin 1
PS1‐NTF: N‐terminal fragment of presenilin 1
PS‐DKO MEF: presenilin 1 and 2 double knockout mouse embryonic fibroblast
RRID: Research Resource Identifier (see scicrunch.org)
SAD: Sporadic Alzheimer's disease

## 1 Introduction

Alzheimer's disease (AD) is characterized by the formation of amyloid plaques and neurofibrillary tangles (NFTs) made up of aggregated β‐amyloid (Aβ) and tau, respectively (Querfurth and LaFerla 2010). Aβ peptides are products of the sequential processing of amyloid precursor protein (APP) by the β‐amyloid cleaving enzyme 1 (BACE1) and the γ‐secretase complex (Hardy and Selkoe 2002), which is comprised of presenilin (PS), nicastrin (NCT), anterior pharynx‐defective phenotype 1 (APH1), and presenilin enhancer 2 (PEN‐2) (Haass and Selkoe 2007; De Strooper et al. 2012; Hur 2022). AD is broadly categorized based on its onset and genetic factors into Sporadic Alzheimer's Disease (SAD) and Familial Alzheimer's Disease (FAD). Autosomal dominant mutations in APP, PS1, and PS2 are associated with early‐onset FAD and result in increased molar ratios of Aβ42/Aβ40, which is uncommon compared to late‐onset SAD (Xu et al. 2016; Islam et al. 2022; Wolfe 2019a; Devkota et al. 2024). The exact cause of SAD is less clear and is believed to result from a complex interplay of genetic, environmental, and lifestyle factors (Bufill et al. 2009). Both SAD and FAD share common clinical features, such as progressive memory loss and cognitive decline, as well as pathological hallmarks, including accumulation of Aβ plaques and tau protein aggregates in the brain (Zhang et al. 2023; DeTure and Dickson 2019). Nevertheless, the precise mechanisms and contributions of Aβ to disease pathogenesis and progression continue to be subjects of active investigation (Chen and Mobley 2019; Hampel et al. 2021).

Molecular chaperones and stress‐response proteins regulate the folding, stability, and clearance of proteins and are particularly important in preventing dysfunction and aggregation of mutated and misfolded proteins (Hartl et al. 2011). Many studies have demonstrated that heat shock protein 90 (Hsp90) is the well‐known molecular chaperone protein that assists in the proper folding of other proteins, stabilizes them under stress, and aids in their degradation (Jolly and Morimoto 2000; Rutledge et al. 2022). Recently, we reported that high temperature regulates Aβ production by modulating γ‐secretase complex formation through the interaction of Hsp90 to the APH1/NCT partially assembled γ‐secretase complex (Noorani et al. 2020). Previous findings have indicated that Hsp90 can influence both Aβ production and tau accumulation through its direct association with γ‐secretase complexes and tau protein (Tortosa et al. 2009; Oroz et al. 2018; Karagoz et al. 2014). However, for Hsp90 to function properly, co‐chaperones regulate its enzymatic (ATPase) and folding activities and guide specific proteins to interact with Hsp90, as it does not operate independently (Backe et al. 2020). One such Hsp90 co‐chaperone is the activator of Hsp90 ATPase homolog 1 (AHA1), which stimulates the ATPase activity of Hsp90. Notably, AHA1 is the only known activator of Hsp90 ATPase activity in mammals (Wolmarans et al. 2016; Mondol et al. 2023). This small 38‐kDa co‐chaperone binds to the N‐terminal and middle domains of Hsp90, inducing a partially closed conformation that dramatically accelerates the ATPase cycle's progression (Li et al. 2013; Oroz et al. 2019). Apart from its role in stimulating the ATPase activity of Hsp90, AHA1 also functions in the late stage of the Hsp90 chaperone cycle. In addition, AHA1 can act independently of Hsp90 as an autonomous chaperone to prevent the aggregation of stressed or misfolded proteins (Panaretou et al. 2002; Lotz et al. 2003; Tripathi et al. 2014; Liu and Wang 2022). Previous research has indicated that individuals with AD have increased levels of AHA1/Hsp90 in their brains when compared to age‐matched controls (Bai et al. 2021). In addition, AHA1 has been found to increase the production of harmful tau aggregates (Shelton et al. 2017; Criado‐Marrero et al. 2021). On the other hand, it has been observed that AHA1 regulates the expression of Dicer1 protein, which plays a key role in the development of cancer (Liu et al. 2022). Another report has suggested that AHA1 may play an oncogenic role in osteosarcoma by increasing the levels of isocitrate dehydrogenase 1 (IDH1) (Zheng et al. 2021). Moreover, AHA1 has been found to increase the misfolding of cystic fibrosis transmembrane conductance regulator, including the Δ508 mutant that is commonly seen in cystic fibrosis patients (Wang et al. 2006). However, the role of AHA1 in regulating the other proteins is largely unknown. Therefore, identifying the proteins that AHA1 interacts with in the Hsp90 network will help us understand AHA1's role in biological processes regulated by Hsp90.

In this study, we hypothesized that AHA1 may regulate the production of Aβ through mechanisms that are independent of Hsp90 interaction or dependent on its interaction with Hsp90. We demonstrated that AHA1 can regulate APP levels through mechanisms that are at least partially independent of Hsp90 interaction while promoting γ‐secretase complex formation through pathways dependent on Hsp90 interaction, ultimately affecting Aβ production. Our findings show that AHA1 is associated with APP and APH1, a component of the immature γ‐secretase complex, highlighting the role of AHA1 in modulating APP and γ‐secretase assembly. Additionally, we found a novel link between the upregulation of AHA1 function and FAD mutations, connecting the mechanism associated with loss of function in FAD mutations and abnormal Aβ production. Our findings support a mechanism by which AHA1 may contribute to AD pathogenesis through the regulation of Aβ production.

## 2 Materials and Methods

### 2.1 Cell Culture and DNA Transfection

HEK‐APP695 cells (derived from HEK293T cells, RRID:CVCL_0063; no RRID available for the stable derivative) were described previously (Mitsuishi et al. 2010). CHO cells (RRID:CVCL_0214) stably overexpressing all human γ‐secretase components WT or PS1 mutants were previously prepared in our lab (Quintero‐Monzon et al. 2011). HEK293T cells (RRID:CVCL_0063), wild‐type mouse embryonic fibroblasts (WT MEFs; no RRID available), and PS1/2 double‐knockout mouse embryonic fibroblasts (PS‐DKO MEFs; no RRID available) were cultured in Dulbecco's Modified Eagle's Medium (DMEM; Thermo Fisher Scientific, cat. no. 11965‐092) at 37°C in 5% CO_2_. CHO cells were grown in DMEM/F12 medium (Thermo Fisher Scientific, cat. no. 11320‐033) under the same conditions. All cell lines were maintained in media supplemented with 10% fetal calf serum (Thermo Fisher Scientific, cat. no. 26140‐079) and 1% penicillin/streptomycin (Thermo Fisher Scientific, cat. no. 15140‐122). DNA transfections were performed using Lipofectamine 3000 (Thermo Fisher Scientific, cat. no. L3000008) according to the manufacturer's instructions. Transfected cells were harvested 48 h post‐transfection for overexpression or knockdown experiments. The AHA1/Hsp90 interaction inhibitor ND‐AHA1‐46 (synthesized as described previously; no RRID available) was added at 10 μM for 24 h in HEK‐APP or PS‐DKO MEF cells prior to harvest. For Hsp90 inhibition experiments, HEK293T cells transfected with APP695 were treated with the Hsp90 inhibitor 17‐AAG (Sigma–Aldrich, cat. no. A8476) at 1 μM for 24 h prior to harvest. All experiments were performed using cells within 20 passages after thawing from frozen stocks to minimize genetic and phenotypic drift. The cell lines used in this study were verified against the International Cell Line Authentication Committee (ICLAC) database and are not listed as commonly misidentified cell lines. All cell lines were obtained from established sources and were routinely tested for mycoplasma contamination during the course of this study.

### 2.2 iPS Cell Culture

Human iPSC lines were obtained from the WiCell Research Institute. The FAD iPSC line carrying the APP‐V717I mutation was HVRDi002‐A (female, 33 years; RRID:CVCL_JV26). The non‐demented control iPSC line was UCSD079i‐1‐12 (female, 32 years; RRID: CVCL_EN22). All iPSC lines were generated from human donors with informed consent prior to repository deposition and were distributed under a material transfer agreement (MTA). Use of these human iPSC lines was considered IRB‐exempt. iPSCs were differentiated into neurons in the laboratory of Dr. Heather M. Wilkins, and neuronal cell lysates and conditioned media were provided for use in this study. Briefly, iPSCs were differentiated into neural progenitor cells (NPCs) using STEMDiff Neural Induction Medium (StemCell Technologies, cat. no. 05835). iPSCs were placed into a single‐cell suspension in NIM with SMAD inhibitor and ROCK inhibitor (StemCell Technologies, cat. no. 72307) on an AggreWell 800 plate (StemCell Technologies, cat. no. 34815). Embryoid bodies were cultured in the AggreWell plate for 5 days with NIM/SMADi partial medium changes daily. Embryoid bodies were then plated onto Matrigel‐coated plates (Corning, cat. 354277) and fed daily with NIM/SMADi medium until Day 12 to allow neural rosette formation. Neural rosettes were selected using Neural Rosette Selection Reagent (StemCell Technologies, cat. no. 05832) and plated onto Matrigel‐coated dishes with NIM/SMADi. Media was changed daily for 7 days, after which neural progenitor cells (NPC) were cryopreserved and split into defined Neural Progenitor Medium (StemCell Technologies, cat. no. 05833). NPCs were plated onto PLO/Laminin coated dishes (Sigma‐Aldrich, cat. no. P3655 and Thermo Fisher Scientific, cat. no. 23017015) in neural progenitor medium. The following day, the media was changed to StemDiff Forebrain Neural Differentiation Medium (StemCell Technologies, cat. no. 0860). Media was changed daily for 7 days (Muratore, Srikanth, et al. 2014; Muratore, Rice, et al. 2014). Following this, cells were plated onto PLO/laminin‐coated dishes in defined Brain Phys Medium (Stemcell Technologies, cat. no. 05790) supplemented with N2A, SM1, BDNF, GDNF, cAMP, and ascorbic acid for neuronal maturation. Neurons were matured for 7–10 days and used for downstream experiments.

### 2.3 Plasmid Constructs

The pcDNA plasmids encoding full‐length human APP695 and APP‐C99 (C‐terminal 99 amino acid residues of APP) were kindly provided by Dr. L. Liu (Harvard Medical School/Brigham and Women's Hospital, USA). The mutant form of the FAD‐linked APP‐C99‐I45F construct and plasmids overexpressing all human γ‐secretase components, including WT and FAD‐linked PS1‐E280A, were generated in our laboratory. The WT AHA1 and AHA1 E67K constructs were provided by Dr. Laura J. Blair (University of South Florida) and were generated using the pCMV6 backbone. All plasmids generated in this study are available from the corresponding authors upon reasonable request.

### 2.4 Quantification of Aβ40 and Aβ42 Levels by ELISA

Levels of secreted Aβ40 and Aβ42 in conditioned medium were quantified using sandwich enzyme‐linked immunosorbent assay (ELISA) kits for human Aβ40 and Aβ42 (Thermo Fisher Scientific, cat. no. KHB3481 for Aβ40 and cat. no. KHB3441 for Aβ42) according to the manufacturer's instructions.

### 2.5 AHA1 Knockdown

AHA1 knockdown experiments were performed using predesigned Stealth siRNA against AHA1 (AHA1 siRNA) and Stealth siRNA negative control (Thermo Fisher Scientific), as previously described (Bettayeb et al. 2016). The siRNA sequences for human AHA1: sense: 5′‐CCAUGCUCCUCCAACAUUATT‐3′ anti‐sense: 5′‐UAAUGUUGCAGGAGCAUGGGT‐3′. The siRNA sequences for mouse AHA1: sense: 5′‐GAACUGGACAGGUACCUTT‐3′ anti‐sense: 5′‐AGAGGUACCUGUCCAGUUCAG‐3′. Briefly, the cells were transiently transfected with 25 pmol AHA1 siRNA or control siRNA using Lipofectamine RNAiMAX (Thermo Fisher Scientific, cat. no. 13778150) according to the manufacturer's instructions. The knockdown effects were examined after 48 h of incubation. The cultures were then processed for ELISA and western blot analysis.

### 2.6 qPCR

Total RNA was isolated from HEK‐APP cells using TRIzol (Thermo Fisher Scientific, cat. no. 15596026) followed by chloroform extraction. RNA was precipitated with isopropanol and washed with 70% ethanol. Equal amounts of RNA were reverse transcribed into cDNA using a cDNA synthesis kit (Bio‐Rad, cat. no. 1708841) according to the manufacturer's instructions. Quantitative PCR was performed using TaqMan Gene Expression Assays (Thermo Fisher Scientific) on a QuantStudio real‐time PCR system (Applied Biosystems). The following assays were used: APP (Hs00169098_m1) and GAPDH (Hs02786624_g1) as the endogenous control. Relative gene expression levels were calculated using the ΔΔCT method after normalization to GAPDH.

### 2.7 Fluorogenic γ‐Secretase Substrate‐Cleavage Assay

Membrane‐associated cleavage of a fluorogenic γ‐secretase substrate was measured using a previously described assay (Vorobyeva et al. 2014). AHA1 was knocked down in HEK293T cells by siRNA or transfected with EV or WT AHA1 or AHA1 E67K mutant grown in 150‐mm culture dishes, collected in ice‐cold PBS, and pelleted at 5000 RPM for 5 min. The pellet was homogenized in 500 μL of Buffer B (20 mM HEPES, pH 7.5, 150 mM KCl, 2 mM EGTA, protease, and phosphatase inhibitors) using a 27‐gauge needle. The resulting homogenate was cleared at 45000 RPM at 4°C for 1 h. The resulting supernatant was collected, and the pellet was washed with 500 μL Buffer B and passed through a 27‐gauge needle on ice. The suspension was cleared again at 45000 RPM for 1 h at 4°C. Supernatant was discarded, and the pellet was resuspended in 75 μL Buffer B + 1% CHAPSO (Sigma–Aldrich, cat. no. C3649) and passed through a 27‐gauge needle on ice. The resulting membrane samples were solubilized on a rotator at 4°C for 2 h. Solubilized samples were cleared at 45000 RPM for 1 h at 4°C; supernatant (total cell membrane) was collected, and the pellet was discarded. 100 μg of protein from cells was used for γ‐secretase assay. 8 μM γ‐secretase fluorogenic substrate (Sigma–Aldrich, cat. no. 565764) was added into samples with or without L‐685458 (Sigma–Aldrich, cat. no. L1790), and the samples were incubated in γ‐secretase assay buffer (100 mM Tris–HCl, pH 6.8, 4 mM EDTA, 0.5% CHAPSO) for 2 h. The values were measured by a plate reader (BioTek) at an excitation wavelength of 355 nm and an emission wavelength of 440 nm.

### 2.8 Western Blot Analysis and Antibodies

Cells were washed with PBS and lysed in 1% digitonin (Sigma–Aldrich, cat. no. D141) or RIPA buffer (25 mM Tris–HCl, pH 7.6, 150 mM NaCl, 1% Nonidet P‐40, 1% sodium deoxycholate, 0.1% SDS) supplemented with protease inhibitor cocktail (Thermo Fisher Scientific, cat. no. 78430). Lysates were incubated on ice for 20–30 min and clarified by centrifugation at 20000×*g* for 15 min at 4°C. Protein concentration was determined using the Pierce BCA Protein Assay Kit (Thermo Fisher Scientific, cat. no. 23225) according to the manufacturer's instructions. Equal amounts of protein (20–30 μg per lane) were mixed with 4× Laemmli sample buffer (Bio‐Rad, cat. no. 1610747) containing β‐mercaptoethanol (Sigma–Aldrich, cat. no. M6250), heated at 95°C for 5 min, and separated by SDS–PAGE using 4%–12% Bis‐Tris gradient gels (Thermo Fisher Scientific, cat. no. NP0321BOX). Proteins were transferred onto PVDF membranes (Millipore‐Sigma, cat. no. IPVH00010) using wet transfer (30 V, 60 min).

Membranes were blocked for 1 h at room temperature in 5% non‐fat dry milk (Bio‐Rad, cat. no. 1706404) prepared in TBST (20 mM Tris–HCl, 150 mM NaCl, 0.1% Tween‐20). Membranes were incubated overnight at 4°C with primary antibodies diluted in blocking buffer. After primary incubation, membranes were washed 3 × 10 min with TBST and incubated with HRP‐conjugated secondary antibodies (Cell Signaling Technology, anti‐rabbit IgG, cat. no. 7074; anti‐mouse IgG, cat. no. 7076) for 1 h at room temperature. Membranes were washed again 3 × 10 min with TBST, and signal was developed using SuperSignal West Pico PLUS Chemiluminescent Substrate (Thermo Fisher Scientific, cat. no. 34580). Chemiluminescent signals were captured using an Azure Biosystems imaging system. Densitometric quantification was performed using ImageJ software (RRID:SCR_002285) on non‐saturated exposures. Band intensities were background‐subtracted and normalized to β‐actin or GAPDH. For reprobing, membranes were stripped using Restore PLUS western Blot Stripping Buffer (Thermo Fisher Scientific, cat. no. 46430) for 10–15 min at room temperature with gentle agitation, washed 3 × 10 min with TBST, re‐blocked in 5% milk/TBST for 30–60 min, and incubated with the next primary antibody. Primary antibodies used for western blot analysis were AHA1 (Abcam, cat. no. ab83036); Hsp70 (Enzo Life Sciences, cat. no. ADI‐SPA‐810‐J); Hsp90 (Cell Signaling Technology, cat. no. 4874S); BACE1 (Cell Signaling Technology, cat. no. 5606S); NCT (Cell Signaling Technology, cat. no. 3632S); PEN2 (Cell Signaling Technology, cat. no. 5451S); PS1‐CTF (Cell Signaling Technology, cat. no. 3622S); Cleaved Notch1 (Cell Signaling Technology, cat. no. 4147S); β‐actin (Cell Signaling Technology, cat. no. 4967S); GAPDH (Cell Signaling Technology, cat. no. 5174S); α‐ tubulin (Cell Signaling Technology, cat. no. 2144S); APP (6E10) (BioLegend, cat. no. 803003); APH1 (BioLegend, cat. no. 823101); PS1‐NTF (BioLegend, cat. no. 811101); and APP (22C11) (Millipore‐Sigma, cat. no. MAB348). For immunoprecipitation experiments, AHA1 antibody (StressMarq, cat. no. SPC‐183) and Hsp90α antibody (Enzo Life Sciences, cat. no. ADI‐SPS‐771‐D) were used.

### 2.9 Blue Native–Polyacrylamide Gel Electrophoresis (BN‐PAGE)

BN‐PAGE was performed as described previously (Swamy et al. 2006). Cells were lysed in a native sample buffer (Thermo Fisher Scientific, cat. no. BN2003) containing 1% digitonin (Sigma–Aldrich, cat. no. D141) and a protease inhibitor mixture (Thermo Fisher Scientific, cat. no. 78430). After centrifugation at 20000×*g* at 4°C for 30 min, the supernatant was separated on a 3%–12% NativePAGE BisTris gel (Thermo Fisher Scientific, cat. no. BN1001BOX) according to the instructions of the Novex BisTris gel system (Thermo Fisher Scientific). The transferred blot was incubated in 20 mL of 8% acetic acid for 15 min to fix the proteins, rinsed with deionized water, and analyzed with western blotting.

### 2.10 Co‐Immunoprecipitation (Co‐IP) Assays

HEK‐APP and HEK293T cells were lysed in lysis buffer containing 1% digitonin (Sigma–Aldrich, cat. no. D141) or RIPA buffer supplemented with protease inhibitor cocktail (Roche Applied Science, cat. no. 11873580001), followed by centrifugation at 20000×*g* for 30 min at 4°C. All IP steps were performed at 4°C. Cell lysates were subjected to reciprocal immunoprecipitation overnight using either anti‐APP antibody (BioLegend, clone 6E10, cat. no. 803003), anti‐AHA1 antibodies (Abcam, cat. no. ab83036; StressMarq, cat. no. SPC‐183), or anti‐Hsp90α antibody (Enzo Life Sciences, cat. no. ADI‐SPS‐771‐D), along with control IgG (Santa Cruz Biotechnology, cat. no. sc‐2027), in the presence of Protein G‐Sepharose or Protein G‐Agarose resin (Abcam, cat. no. ab193259; Thermo Fisher Scientific, cat. no. 20398). The beads were washed five times with lysis buffer supplemented with protease inhibitor cocktail. Samples were resolved on 4%–12% gradient SDS‐PAGE gels and transferred to polyvinylidene difluoride membranes for Western blot analysis.

### 2.11 Immunostaining

HEK‐APP cells were fixed in 2% paraformaldehyde. Next, they were permeabilized with 0.1% Triton X‐100 (Sigma–Aldrich, cat. no. T8787) and blocked for 45 min in 10% normal goat serum (Thermo Fisher Scientific, cat. no. 50062Z) in PBS. Fixed cells were incubated with primary antibodies (AHA1 and APP or APH1) for 12 h at 4 C. Immunofluorescent labeling was carried out with Alexa Fluor 488– and Alexa Fluor 568–tagged secondary antibodies (Thermo Fisher Scientific, goat anti‐rabbit IgG Alexa Fluor 488, cat. no. A11008; goat anti‐mouse IgG Alexa Fluor 568, cat. no. A11004). The mounting was done with Vectashield reagent with DAPI (Vector Laboratories, cat. no. H‐1200). Images were captured on a confocal microscope (Leica) using an oil‐immersion plan Apo × 60 A/1.40 numerical aperture objective lens.

### 2.12 Statistical Analysis

Statistical analysis was performed using GraphPad Prism 10 software (GraphPad Software, San Diego, CA; RRID:SCR_002798). No statistical methods were used to predetermine sample size. Sample sizes were selected based on previous studies using similar cellular models and experimental approaches and are consistent with previously published studies in the field (Noorani et al. 2020; Islam et al. 2022). All experiments were performed with at least three independent biological replicates. Data are presented as mean ± SEM. Data distribution was assessed for normality using the Shapiro–Wilk test when sample size permitted. No formal test for outliers was conducted, and no data points were excluded from the analysis. Two‐tailed Student's *t*‐test was used for comparisons between two groups. For comparisons among three or more groups, one‐way analysis of variance (ANOVA) followed by Tukey's multiple‐comparison test was used. For experiments involving two independent variables, two‐way ANOVA followed by Tukey's multiple‐comparison test was performed. A *p*‐value < 0.05 was considered statistically significant. Detailed statistical results, including ANOVA outputs and post hoc multiple‐comparison tests, are provided in the Supporting Information: Statistical Tables.

## 3 Results

### 3.1 AHA1 Regulates the Production of Aβ and the Levels of APP Through a Mechanism Partially Independent of Hsp90 Interaction

**FIGURE 1:**
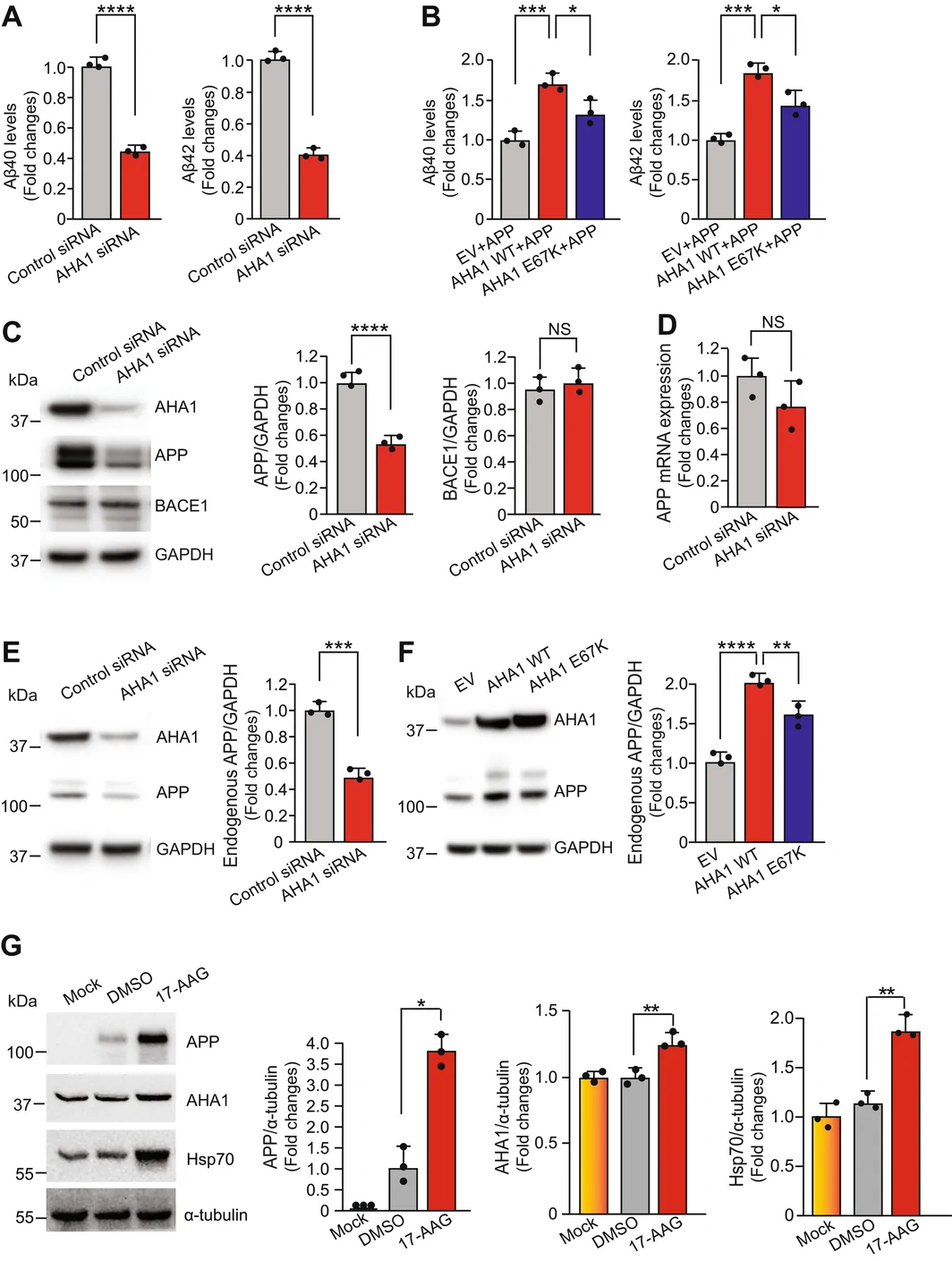
AHA1 controls Aβ production and regulates APP levels through mechanisms that are at least partially independent of its interaction with Hsp90. (A) The amount of secreted Aβ40 and Aβ42 levels in the media of HEK‐APP cells transfected with AHA1 siRNA or Control siRNA were measured with an Aβ ELISA kit. (B) The amount of secreted Aβ40 and Aβ42 levels in the media of HEK293T cells transiently co‐transfected with APP695 and either WT AHA1 or AHA1 E67K or EV were measured with an Aβ ELISA kit. (C) The expression levels of AHA1, full‐length APP, and BACE1 in HEK‐APP cells after the knockdown of AHA1 were detected with western blotting. (D) mRNA expression of APP assessed by qPCR in HEK‐APP cells after knockdown of AHA1. (E) The expression levels of AHA1 and full‐length endogenous APP in HEK293T cells after knockdown of AHA1 were detected with western blotting. (F) The expression levels of AHA1 and full‐length endogenous APP in HEK293T cells after overexpression of WT AHA or AHA1 E67K mutant were determined using western blotting. (G) HEK293T cells transiently expressing APP695 were treated with the Hsp90 inhibitor 17‐AAG for 24 h, and the expression levels of APP, AHA1, and Hsp70 were analyzed by western blotting. Data are presented as mean ± SEM (*n* = 3 independent cell culture preparations). Statistical significance was determined using a two‐tailed Student's *t*‐test for comparisons between two groups or one‐way ANOVA followed by Tukey's multiple‐comparison test for multiple groups. *p < 0.05; **p < 0.01; ***p < 0.001; ****p < 0.0001; NS, not significant.

Because AHA1 can affect the accumulation of toxic tau species (Shelton et al. 2017), we investigated whether altering AHA1 expression can affect Aβ production. To determine the role of AHA1 in Aβ production, we performed the knockdown of AHA1 by transfecting siRNA in human embryonic kidney 293 T (HEK293T) cells, stably overexpressing human APP695. Interestingly, we found that the levels of Aβ40 and Aβ42 were significantly decreased in the culture medium of AHA1 siRNA transfected cells compared to control siRNA‐transfected cells (Figure 1A). To determine whether the reduction in Aβ levels was solely due to decreased substrate availability, we normalized Aβ40 and Aβ42 levels to full‐length APP. The reduction in Aβ40 and Aβ42 remained evident after normalization (Figure S6A), consistent with an effect on APP processing beyond altered substrate abundance; however, this normalization does not measure catalytic efficiency per assembled γ‐secretase complex. We next examined whether AHA1 overexpression enhances Aβ production in HEK293T cells. We co‐transfected HEK293T cells with human APP695 and either wild‐type (WT) AHA1 or AHA1 E67K (a mutant that cannot bind to Hsp90) or an empty vector (EV) control. We found that WT AHA1 significantly increased the levels of secreted Aβ40 and Aβ42 compared to EV control, whereas the AHA1 E67K mutant enhanced Aβ40 and Aβ42 secretion to a lesser degree than WT AHA1 (Figure 1B). These results indicate that AHA1 positively regulates Aβ40 and Aβ42 production in this system.

To gain mechanistic insight into how AHA1 affects Aβ production, we investigated the cellular levels of APP and BACE1 in HEK‐APP cells where AHA1 expression was knocked down using siRNA. Notably, we found that the cellular levels of APP were dramatically reduced; however, the BACE1 levels remained unchanged in AHA1 knocked‐down cells compared to control siRNA‐transfected cells (Figure 1C). This finding implies that the reduction of AHA1 in cells leads to a decrease in exogenous APP levels. We also examined the mRNA expression levels of AHA1 in the HEK‐APP cells after the knockdown of AHA1 using siRNA. We found that silencing AHA1 did not significantly alter APP mRNA levels (Figure 1D), indicating that AHA1 does not regulate APP at the transcriptional level. Instead, the reduction in APP protein upon AHA1 knockdown is likely due to post‐transcriptional mechanisms such as impaired folding, maturation, trafficking, or increased degradation.

Furthermore, the AHA1 knockdown also reduced endogenous APP expression in HEK293T cells (Figure 1E). In addition, transfection of WT AHA1 and mutant AHA1 E67K in HEK293T cells resulted in an increase in endogenous APP protein levels compared to EV control (Figure 1F). To further investigate whether APP regulation by AHA1 depends on Hsp90 activity, HEK293T cells expressing APP695 were treated with the Hsp90 inhibitor 17‐AAG. Interestingly, 17‐AAG treatment increased APP protein levels compared with DMSO‐treated cells and was also associated with elevated Hsp70 and AHA1 expression, consistent with previous studies showing that Hsp90 inhibition induces AHA1 and Hsp70 as part of the cellular heat shock response (Holmes et al. 2008) (Figure 1G). These findings suggest that AHA1 can regulate Aβ production by modulating APP levels through mechanisms that are at least partially independent of Hsp90 interaction.

### 3.2 AHA1 Modulates γ‐Secretase Components and Membrane‐Associated Substrate Cleavage Through a Pathway Dependent on Hsp90 Interaction

**FIGURE 2:**
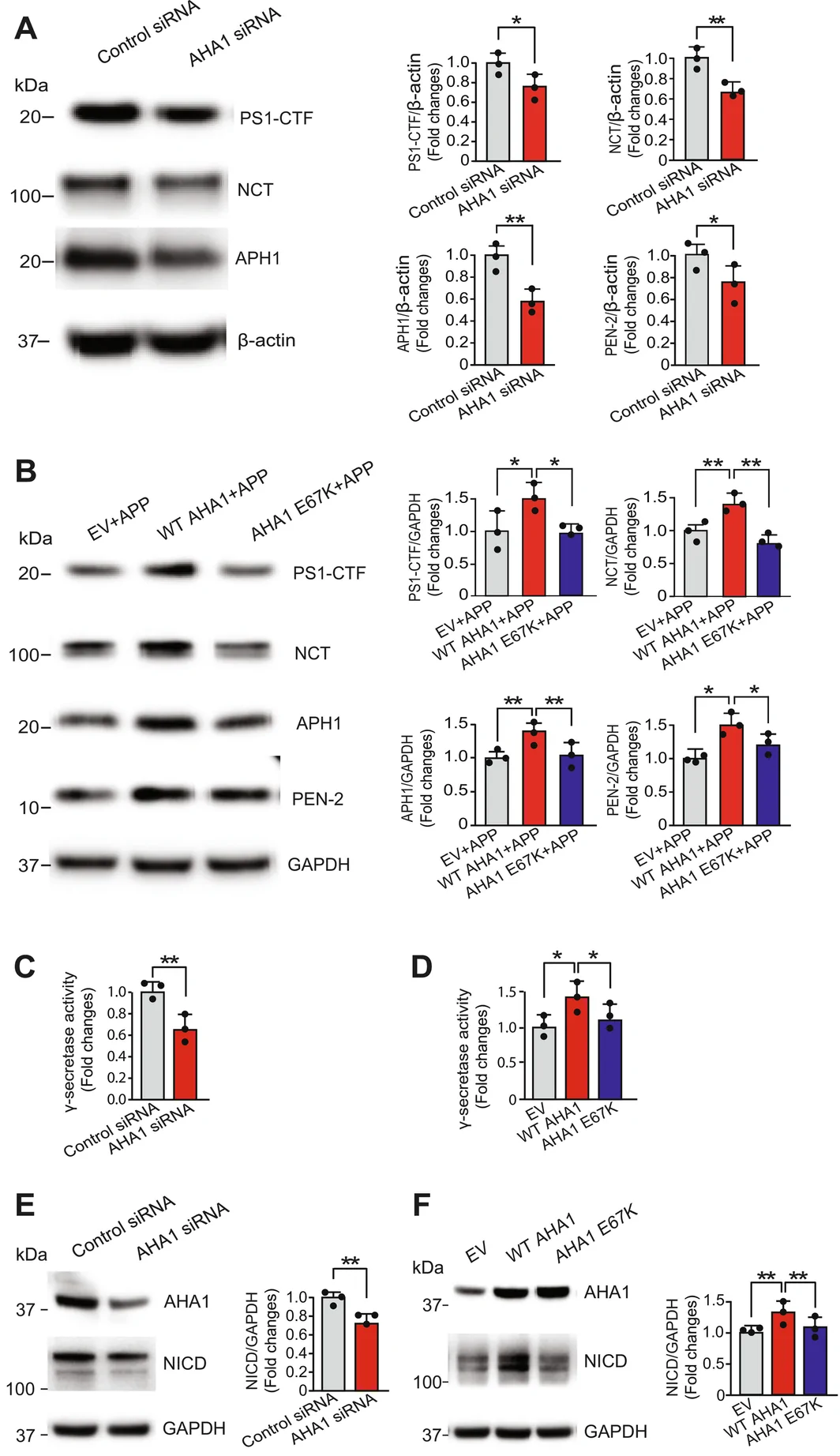
AHA1 modulates γ‐secretase components and membrane‐associated fluorogenic substrate cleavage through an Hsp90‐dependent pathway. (A) The expression levels of PS1‐CTF, NCT, and APH1 in HEK‐APP cells after the knockdown of AHA1 were detected with western blotting. (B) The expression levels of PS1‐CTF, NCT, APH1, and PEN‐2 in HEK293T cells after overexpression of WT AHA1 or AHA1 E67K mutant were detected with western blotting. (C) Cleavage of a fluorogenic γ‐secretase substrate was measured in membrane preparations from HEK293T cells following AHA1 knockdown. (D) Cleavage of a fluorogenic γ‐secretase substrate was measured in membrane preparations from HEK293T cells following overexpression of WT AHA1 or the AHA1 E67K mutant. (E) The expression levels of AHA1 and NICD in WT MEF cells after the knockdown of AHA1 were detected with western blotting. (F) The expression levels of AHA1 and NICD in WT MEF cells after overexpression of WT AHA1 or AHA1 E67K mutant were detected with western blotting. Data are presented as mean ± SEM (*n* = 3 independent cell culture preparations). Statistical significance was determined using a two‐tailed Student's *t*‐test for comparisons between two groups or one‐way ANOVA followed by Tukey's multiple‐comparison test for multiple groups. *p < 0.05; **p < 0.01; ***p < 0.001.

AHA1 is known to stimulate the ATPase activity of Hsp90, thereby promoting the folding of its client proteins (Wolmarans et al. 2016). While reducing Hsp90 levels decreased γ‐secretase components (Noorani et al. 2020), it is unknown whether altering the Hsp90 activity by AHA1 affects γ‐secretase levels. Interestingly, we found that knockdown of AHA1 by using AHA1 siRNA significantly reduced the levels of C‐terminal fragment of presenilin 1 (PS1‐CTF), NCT, and APH1 γ‐secretase components compared to control siRNA (Figure 2A). In contrast, transient co‐transfection of WT AHA1 and APP (AHA1 + APP) in HEK293T cells remarkably increased the levels of PS1‐CTF, NCT, APH1, and PEN‐2 compared to EV control (Figure 2B). These findings suggest that AHA1 modulates the expression of γ‐secretase components by altering Hsp90 activity.

To investigate whether this modulation depends on the interaction between AHA1 and Hsp90, we co‐transfected Hsp90‐binding‐defective mutant AHA1 E67K and APP in HEK293T cells. As expected, the mutant AHA1 E67K + APP overexpression decreased the levels of γ‐secretase components compared to WT AHA1 + APP overexpression or with levels equivalent to the EV + APP overexpression (Figure 2B). Consistent with this finding, we also observed a significant decrease in the complex formation between Hsp90 and AHA1 in HEK293T cells upon treatment with the AHA1/Hsp90 small molecule disruptor ND‐AHA1‐46 (Figure S1A,B). ND‐AHA1‐46 is a small‐molecule inhibitor that selectively interferes with the AHA1–Hsp90 protein–protein interaction by binding the N‐terminal region of AHA1 and reducing its stimulation of Hsp90 ATPase activity. Unlike classical ATP‐competitive Hsp90 inhibitors, ND‐AHA1‐46 does not broadly inhibit Hsp90 function or induce the canonical heat‐shock response including Hsp70 upregulation. Previous research has shown that ND‐AHA1‐46 can reduce P301L tau aggregation in vitro (Keegan et al. 2022). Furthermore, we also observed that blocking AHA1/Hsp90 complex formation with ND‐AHA1‐46 for 24 h reduced the levels of N‐terminal fragment of presenilin 1 (PS1‐NTF), NCT and APH1 γ‐secretase components compared to DMSO control (Figure S2A). However, the cellular levels of APP were significantly increased in ND‐AHA1‐46‐treated cells (Figure S2A). In addition, using blue‐native polyacrylamide gel electrophoresis (BN‐PAGE), the formation of PS1‐NTF, NCT, and APH1 γ‐secretase complex was also remarkably downregulated in ND‐AHA1‐46‐treated cells (Figure S2B). Taken together, these findings suggest that modulation of the expression of γ‐secretase components by AHA1 depends on the AHA1/Hsp90‐associated mechanism.

To identify the role of AHA1 on γ‐secretase assembly in the absence of PS, we observed the precomplex formation in PS1/2 double‐KO (PS‐DKO) mouse embryonic fibroblasts (MEFs) at ~240 kDa. We found that blockade of AHA1/Hsp90 interaction using ND‐AHA1‐46 for 24 h significantly reduced the precomplex formation compared to DMSO control (Figure S2C). This result suggests that AHA1/Hsp90 interaction is also crucial for γ‐secretase assembly irrespective of the PS‐mediated γ‐secretase assembly.

AHA1 knockdown reduced cleavage of the fluorogenic γ‐secretase substrate in HEK293T membrane preparations (Figure 2C). Conversely, WT AHA1 overexpression increased the fluorogenic signal compared with EV, whereas AHA1 E67K produced a lower signal than WT AHA1 and did not differ from EV (Figure 2D). Because equal amounts of total membrane protein were assayed without normalization to assembled γ‐secretase complex abundance, these data do not establish altered enzymatic potency or catalytic efficiency per complex. Together with the BN‐PAGE results, the findings are consistent with AHA1/Hsp90‐dependent changes in the abundance of functional γ‐secretase complexes.

Because high temperature increases the levels of Hsp90 and Notch1 intracellular domain (NICD) in mouse brains (Noorani et al. 2020), we further investigated whether AHA1 can also affect the cleavage of other substrates of γ‐secretase. We used WT MEF cells because HEK293T cells express trace endogenous NICD. Notably, we found that knockdown of AHA1 significantly decreased the NICD levels in WT MEF cells (Figure 2E). Overexpression of WT AHA1 increased the NICD levels in WT MEF cells (Figure 2F). However, mutant AHA1 E67K decreased the levels of NICD compared to WT AHA1 (Figure 2F). Overexpression of AHA1 E67K did not change NICD levels compared to EV control (Figure 2F). These findings indicate that AHA1 can play a role in regulating the γ‐secretase‐mediated cleavage of Notch. Collectively, these results suggest that AHA1 regulates γ‐secretase components and substrate processing through a pathway that depends on Hsp90 interaction.

### 3.3 AHA1 Associates With APP and APH1

**FIGURE 3:**
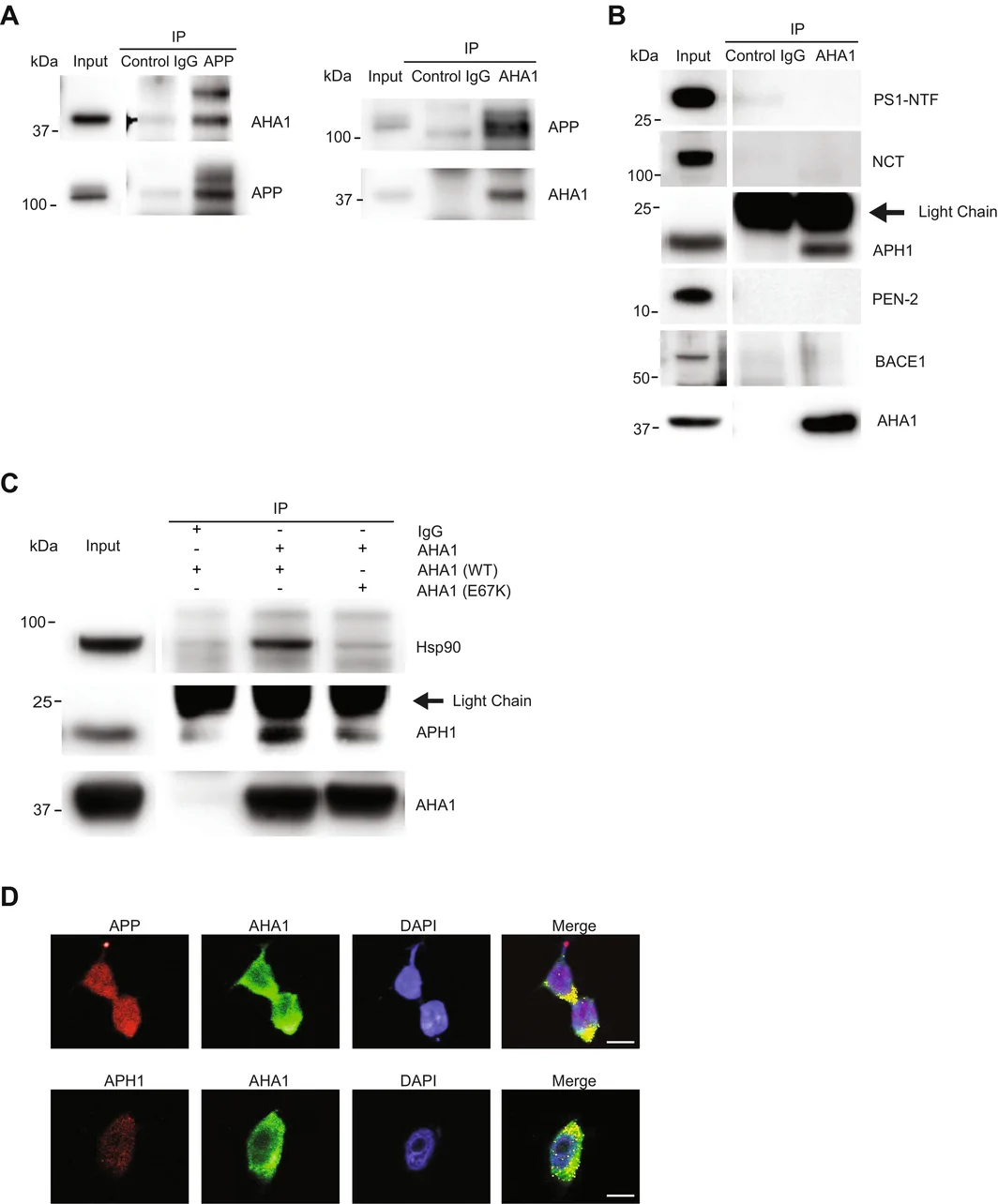
AHA1 is bound to both APP and APH1. (A) Lysates from HEK‐APP cells at 48 h were immunoprecipitated with the APP antibody (clone 6E10), and the immunoprecipitates were analyzed by western blotting using the AHA1 antibody. Reciprocal immunoprecipitation was also performed using the AHA1 antibody followed by immunoblotting for APP. (B) Lysates from HEK‐APP cells at 48 h were immunoprecipitated with the AHA1 antibody. The obtained samples were subjected to western blotting and analyzed using antibodies that recognize γ‐secretase components and BACE1. (C) HEK293T cells transfected with either WT AHA1 or AHA1 E67K. Lysates from cells were immunoprecipitated with the AHA1 antibody. The obtained samples were subjected to western blotting and analyzed using Hsp90 and APH1 antibodies. (D) Immunofluorescence analysis showed that AHA1 colocalized with APP or APH1. HEK‐APP cells were stained with secondary antibodies labeled Alexa Fluor 488 and Alexa Fluor 568: AHA1 (green), APP (red), and APH1 (red). Nuclear boundaries were determined with DAPI (blue). Data are obtained from three independent biological experiments (independent cell culture preparations). Scale bar (D): 10 μm.

To investigate whether AHA1 is involved in the regulation of APP and the formation of the γ‐secretase complex, we performed immunoprecipitation (IP) experiments using HEK293T cells. We found that AHA1 is associated with APP, and reciprocal IP of APP also pulled down AHA1 (Figure 3A), supporting a consistent cellular association between these proteins. In contrast, we did not detect an interaction between Hsp90 and APP (Figure S7A), suggesting that AHA1‐mediated regulation of APP stability may occur at least partially independently of direct Hsp90 association. In addition, AHA1 interacted with APH1 but not with the other γ‐secretase components PS1‐NTF, NCT, or PEN‐2 (Figure 3B). These results suggest that AHA1 is involved in the regulation of APP and the formation of the γ‐secretase complex. However, AHA1 did not interact with BACE1 (Figure 3B), suggesting that AHA1 regulates Aβ production through interacting with APP and γ‐secretase components without hampering β‐secretase activity. To confirm further that AHA1 regulates γ‐secretase by a pathway dependent on Hsp90 interaction, we overexpressed the WT AHA1 and AHA1 E67K mutant in HEK‐293 T cells and performed IP experiments. As reported previously (Shelton et al. 2017), we found that AHA1 E67K mutant showed a weaker interaction with Hsp90 than WT AHA1 (Figure 3C). Interestingly, AHA1 E67K mutant showed reduced interaction with APH1 compared with WT AHA1 (Figure 3C). These results suggest that the AHA1/Hsp90 complex plays a crucial role in regulating γ‐secretase assembly. In addition, confocal microscopy studies demonstrated that colocalization between AHA1 and APP or APH1 (Figure 3D) is consistent with the findings in the IP studies. These results suggest that AHA1 may promote the assembly of the γ‐secretase, possibly first by forming the precomplex through its interaction with APH1 and modulating Aβ production by interacting with APP and APH1.

### 3.4 AHA1 Modulates the Formation of the γ‐Secretase Complex

**FIGURE 4:**
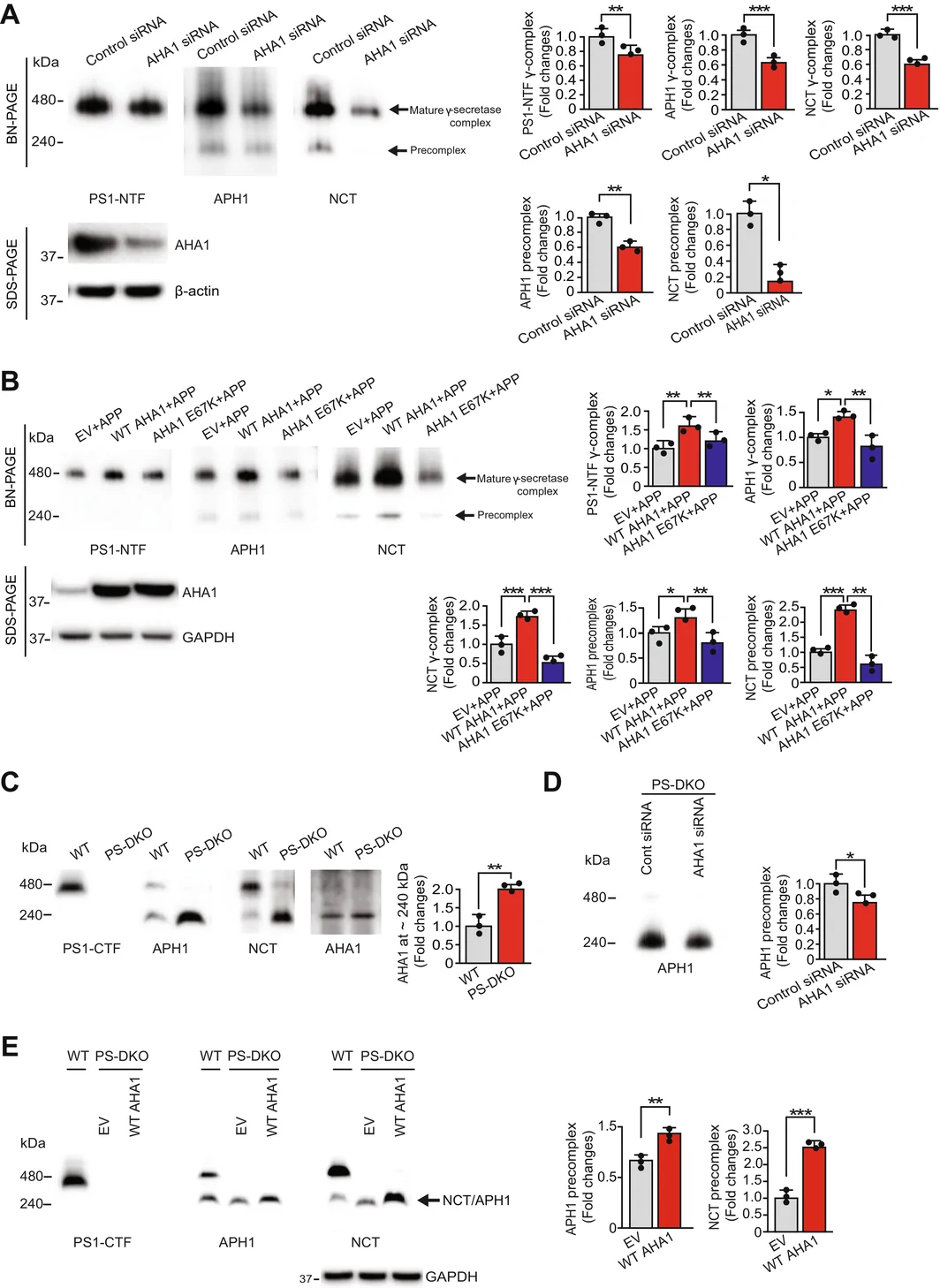
AHA1 regulates the assembly of the γ‐secretase complex. (A) 10 μg of protein from cells transfected with control siRNA or AHA1 siRNA were subjected to BN‐PAGE or SDS‐PAGE and analyzed with western blotting using the indicated antibodies. (B) 10 μg of protein from cells co‐transfected with EV + APP or WT AHA1 + APP or AHA1 E67K + APP cells were subjected to BN‐PAGE or SDS‐PAGE and were analyzed with western blotting using the indicated antibodies. (C) 10 μg of protein from WT or PS‐DKO MEF cells were subjected to BN‐PAGE and analyzed with western blotting using the indicated antibodies. (D) PS‐DKO MEF cells transfected with control siRNA or AHA1 siRNA were subjected to BN‐PAGE and analyzed with western blotting using APH1 antibody. (E) PS‐DKO MEF cells transfected with EV or WT AHA1 were subjected to BN‐PAGE or SDS‐PAGE and analyzed with western blotting using the indicated antibodies. Data are presented as mean ± SEM (*n* = 3 independent cell culture preparations). Statistical significance was determined using a two‐tailed Student's *t*‐test for comparisons between two groups or one‐way ANOVA followed by Tukey's multiple‐comparison test for multiple groups. *p < 0.05; **p < 0.01; ***p < 0.001.

As a previous study reported that knockdown of Hsp90 significantly reduced the levels of precomplex and mature complex of γ‐secretase (Noorani et al. 2020), we further investigated whether the AHA1/Hsp90 complex affects the formation of the γ‐secretase complex. Given that the precomplex of γ‐secretase is formed by APH1 and NCT before the formation of the mature γ‐secretase complex (De Strooper et al. 2012; Wolfe 2019b), in BN‐PAGE, the band at ∼440 kDa represents the mature γ‐secretase, while the band at ∼240 kDa represents the APH1/NCT precomplex (Jung et al. 2020). Using BN‐PAGE, we found that knockdown of AHA1 decreased the levels of both precomplex (Figure 4A, lower panel at ∼240 kDa) and mature γ‐secretase complex (Figure 4A, upper panel at ∼440 kDa) in HEK‐APP cells. Similarly, we also found that knockdown of AHA1 remarkably decreased the mature γ‐secretase complex levels of PS1‐NTF and APH1 as well as the levels of APH1 precomplex in WT MEF cells (Figure S3A). On the other hand, co‐transfection of WT AHA1 + APP significantly increased the levels of both precomplex and mature γ‐secretase complex compared to EV + APP control in HEK293T cells (Figure 4B). However, co‐transfection of AHA1 E67K + APP did not upregulate the levels of precomplex and mature γ‐secretase complex compared to WT AHA1 + APP or EV + APP (Figure 4B). These results indicate that AHA1 is positively correlated with the formation of the γ‐secretase complex and suggest that AHA1/Hsp90 complexes are essential for regulating γ‐secretase assembly. We further verified our findings by treating HEK‐APP cells with ND‐AHA1‐46 inhibitor for 24 h. We found that the formation of the APH1/NCT precomplex and mature γ‐secretase complex formation was remarkably decreased in these cells compared to DMSO (Figure S2B), indicating that AHA1 positively regulates the γ‐secretase complex formation through its interaction with Hsp90.

AHA1 knockdown reduced the steady‐state levels of multiple γ‐secretase subunits (PS1‐CTF, NCT, APH1, and PEN2), so we cannot completely exclude the possibility that the decreased APH1/NCT precomplex and mature γ‐secretase bands observed by BN‐PAGE partially reflect reduced subunit abundance. Thus, the current data do not definitively distinguish whether AHA1 primarily stabilizes unassembled γ‐secretase subunits or directly promotes complex assembly. However, the concurrent reduction of both the ~240‐kDa precomplex and the ~440‐kDa mature complex, together with decreased APH1 and NCT levels and the observed interaction between AHA1 and APH1, support a model in which AHA1 contributes to γ‐secretase homeostasis by influencing both subunit stability and complex formation.

The formation of the active γ‐secretase complex is thought to begin with an intermediate APH1/NCT precomplex, which is then followed by the addition of PS and PEN‐2 (Wolfe 2019b; De Strooper et al. 2012). In the absence of PS, the mature γ‐secretase complex cannot form, resulting in the accumulation of the APH1/NCT precomplex (Liu et al. 2013; Jung et al. 2020). This led us to hypothesize that the AHA1/Hsp90 complex might regulate the formation of the mature γ‐secretase complex by controlling the formation of the APH1/NCT precomplex. We observed that in the absence of PS, mature γ‐secretase complex formation was completely abolished, leading to the accumulation of the APH1/NCT precomplex (∼240 kDa band) in PS‐DKO MEF cells, as reported previously (Figure 4C). Interestingly, we found that AHA1 did not incorporate into the mature γ‐secretase complex but instead co‐migrated with the APH1/NCT precomplex at ~240 kDa (Figure 4C). Additionally, we observed that the expression levels of AHA1 were elevated in PS‐DKO cells compared to WT MEF cells (Figure 4C). This increase in AHA1 expression may reflect a compensatory proteostatic response to stalled γ‐secretase assembly in the absence of presenilin. These findings indicate that AHA1 may play a role in promoting the assembly of the APH1/NCT precomplex, thereby facilitating the formation of the γ‐secretase mature complex through its initial interaction with APH1. We further investigated the role of AHA1 on γ‐secretase precomplex formation in PS‐DKO fibroblasts by BN‐PAGE. We observed that knockdown of AHA1 significantly decreased the levels of APH1/NCT precomplex formation at ~240 kDa in PS‐DKO fibroblasts compared to control siRNA (Figure 4D). Similar results were obtained when PS‐DKO cells were treated with ND‐AHA1‐46 (Figure S2C). Overexpression of WT AHA1 remarkably increased the levels of APH1/NCT precomplex formation in PS‐DKO fibroblasts compared to EV control (Figure 4E). Collectively, these findings suggest that AHA1 can modulate γ‐secretase complex formation by regulating APH1/NCT γ‐secretase precomplex formation, and this modulation of γ‐secretase complex is positively correlated with AHA1 expression.

### 3.5 AHA1 Controls Abnormal Aβ Production in Cells Expressing an FAD‐Linked APP Mutation

**FIGURE 5:**
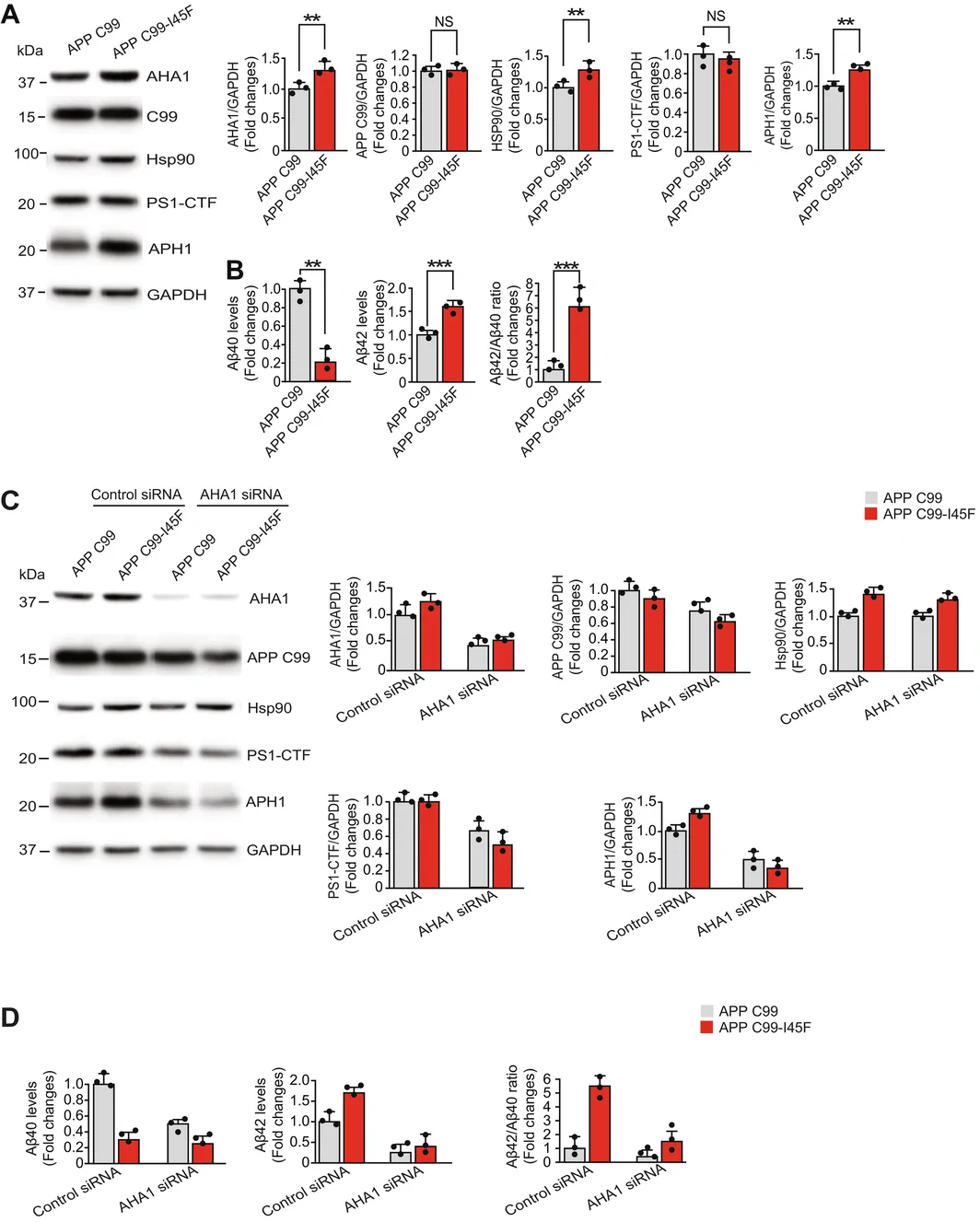
AHA1 controls abnormal Aβ production in HEK293T cells expressing an FAD‐linked APP mutation. (A) Transient transfection of APP C99 or FAD‐linked APP C99 mutant I45F (APP C99‐I45F) in HEK293T cells. The expression levels of AHA1, APP C99, Hsp90, PS1‐CTF, and APH1 were detected with western blotting. (B) The amount of secreted Aβ40 and Aβ42 levels in the media of HEK293T cells transfected with APP C99 or APP C99‐I45F were measured with an Aβ ELISA kit. (C) Expression levels of AHA1, APP C99, Hsp90, PS1‐CTF, and APH1 were assessed by western blotting in HEK293T cells after co‐expressing AHA1 knockdown and APP C99 or APP C99‐I45F. (D) The amount of secreted Aβ40 and Aβ42 levels in the media of the HEK293T cells after co‐expressing AHA1 knockdown and APP C99 or APP C99‐I45F was measured with an Aβ ELISA kit. Data are presented as mean ± SEM (*n* = 3 independent cell culture preparations). Statistical significance was determined using a two‐tailed Student's *t*‐test for comparisons between two groups and two‐way ANOVA followed by Tukey's multiple‐comparison test for panels C and D. *p < 0.05; **p < 0.01; ***p < 0.001; NS, not significant.

Because Hsp90 stabilizes mutant proteins and prevents their degradation by interacting with them (Koren 3rd et al. 2009; Trepel et al. 2010; Whitesell and Lindquist 2005), we hypothesized that APP mutants could be a client protein of Hsp90 that is modulated by AHA1. To test this, we transiently transfected FAD‐linked APP C99‐I45F mutant or APP C99 as a control in HEK293T cells. Interestingly, we found that overexpression of APP C99‐I45F mutant remarkably increased the levels of AHA1, Hsp90, and γ‐secretase components APH1 expression but not the expression of PS1‐CTF compared to APP C99 control (Figure 5A). In a separate observational comparison, age‐ and sex‐matched APP‐V717I iPSC‐derived neurons showed an increased Aβ42/Aβ40 ratio (Figure S4A), increased levels of AHA1, Hsp90, APP, and PS1‐NTF (Figure S4B), and increased PS1‐NTF‐containing mature γ‐secretase complex abundance compared with WT controls (Figure S4C). Because AHA1 was not experimentally manipulated in these neurons, these findings establish an association between the APP‐V717I genotype, AHA1 abundance, and γ‐secretase‐related measures, but do not demonstrate that AHA1 causes the neuronal changes. Furthermore, transfection of APP C99‐I45F mutant did not alter the levels of C99 (Figure 5A), suggesting that upregulation of AHA1 and Hsp90 depends on the APP C99‐I45F mutation regardless of the levels of the C99 proteins. We further conducted a deeper investigation into the impact of the APP C99‐I45F mutant on Aβ secretion. We found that the levels of Aβ40 secretion were dramatically reduced, and the levels of Aβ42 secretion were dramatically increased in APP C99‐I45F transfected cells compared to APP C99 control. In consequence, the levels of Aβ42/Aβ40 ratio were dramatically upregulated in APP C99‐I45F transfected cells (Figure 5B) as reported previously (Devkota et al. 2021). These results show that the FAD‐linked APP mutation was associated with increased AHA1/Hsp90 and APH1 abundance and an increased Aβ42/Aβ40 ratio.

We examined the effect of AHA1 knockdown on the levels of C99, Hsp90, PS1‐CTF, and APH1 in HEK293T cells transfected with both C99 and C99‐I45F mutants. We observed that the expression levels of C99, PS1‐CTF, and APH1 were significantly decreased in AHA1 knockdown cells as compared to control siRNA of APP C99 and APP C99‐I45F (Figure 5C). However, Hsp90 levels did not change after AHA1 knockdown in these cells (Figure 5C). To investigate whether AHA1 upregulation by the influence of FAD‐linked APP mutants is essential for abnormal Aβ production, we performed a knockdown of AHA1 using siRNA in HEK293T cells transfected with both APP C99 and APP C99‐I45F mutants. We found that the knockdown of AHA1 remarkably decreased the levels of Aβ40 in APP‐C99 transfected cells compared to the APP C99 control siRNA (Figure 5D). However, the knockdown of AHA1 did not significantly alter the levels of Aβ40 in APP‐C99‐I45F transfected cells compared to the APP‐C99‐I45F control siRNA (Figure 5D). On the other hand, knockdown of AHA1 remarkably decreased the levels of Aβ42 both in APP C99 and APP‐C99‐I45F transfected cells compared to the control siRNA of APP‐C99 and APP C99‐I45F, resulting in the reduction of Aβ42/Aβ40 ratio (Figure 5D). To determine whether these effects were solely due to altered substrate abundance, Aβ40 and Aβ42 levels were normalized to C99 expression levels (Aβ40/C99 and Aβ42/C99). After normalization, the pattern and statistically significant Tukey pairwise comparisons indicated in Figure S6B showed that altered C99 abundance did not fully account for all changes in Aβ output. We therefore do not infer a uniform reduction across all peptide and genotype conditions or a change in catalytic efficiency per assembled γ‐secretase complex. Taken together, these results suggest that FAD‐linked APP mutant (APP C99‐I45F) mutation increases the AHA1 level, which in turn contributes to an increased level of γ‐secretase, mainly APH1, and leads to abnormal Aβ production.

### 3.6 AHA1 Regulates Abnormal Aβ Production and APP Expression in FAD‐Linked PS1 Mutants

**FIGURE 6:**
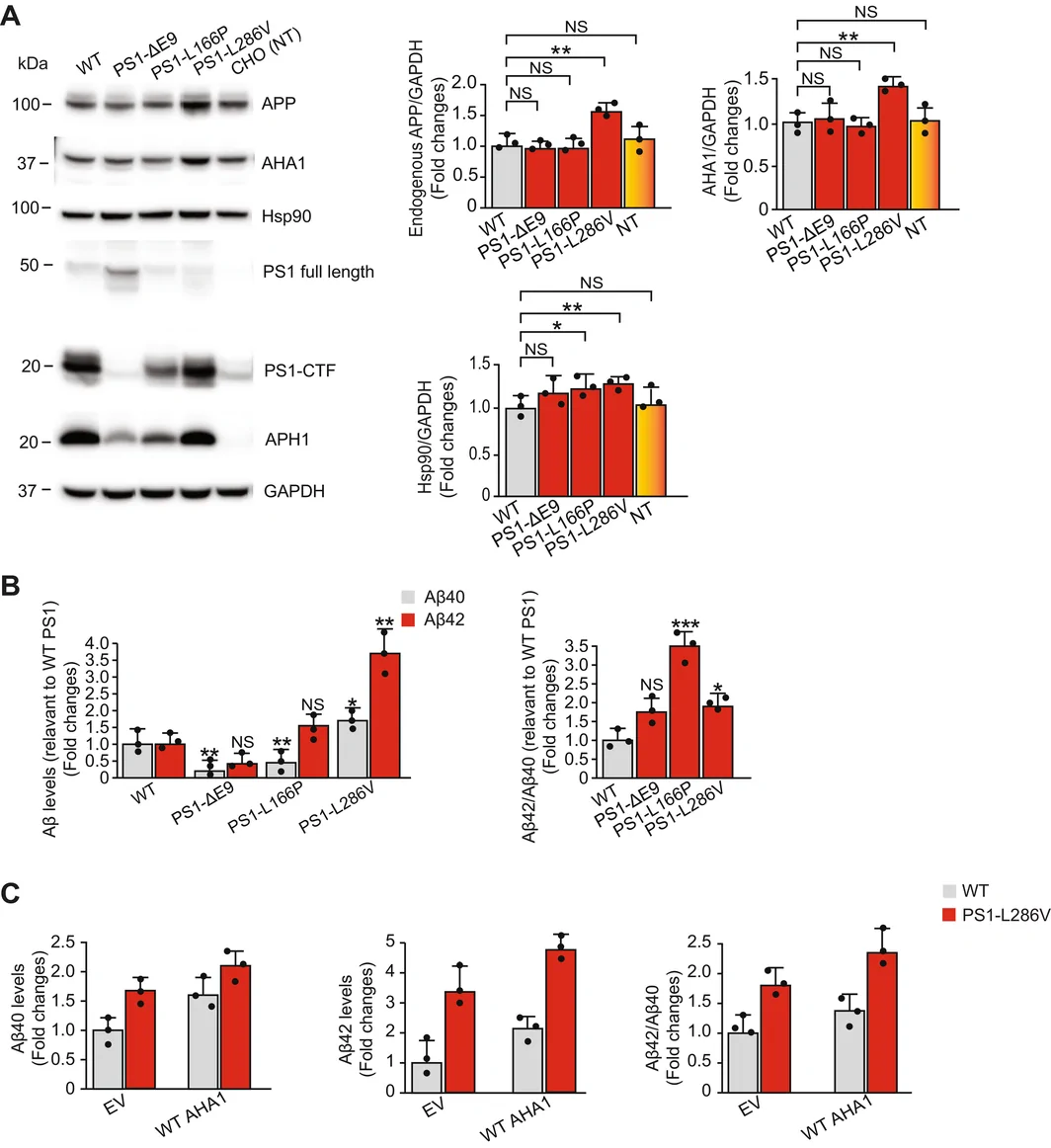
AHA1 modulates abnormal Aβ production and APP expression in cells carrying FAD‐linked PS1 mutations. (A) The expression levels of endogenous full‐length APP, AHA1, Hsp90, full‐length PS1, PS1‐CTF, and APH1 were assessed by western blotting in CHO cells stably overexpressing WT or PS1 mutants. NT‐ non‐transfected parental CHO cells. (B) The amount of secreted Aβ40 and Aβ42 levels in the media of CHO cells stably overexpressing WT or PS1 mutants transfected with APP695 were measured with an Aβ ELISA kit. (C) The levels of secreted Aβ40 and Aβ42 in the media of CHO cells stably overexpressing WT or PS1‐L286V were co‐transfected with APP695 and either WT AHA1 or EV control. Data are presented as mean ± SEM (*n* = 3 independent cell culture preparations). Statistical significance was determined using one‐way ANOVA followed by Tukey's multiple‐comparison test for panels A and B, and two‐way ANOVA followed by Tukey's multiple‐comparison test for panel C. *p < 0.05; **p < 0.01; ***p < 0.001; NS, not significant.

Previous studies have shown that certain PS1 mutations linked to FAD upregulate the expression of APP and may produce more abnormal Aβ production. These findings were reported using various cell lines, including iPSC‐derived neurons (Li et al. 2018; Ochalek et al. 2017; Podvin et al. 2021; Hook et al. 2023; Woodruff et al. 2016), Neuro2a (N2a) cells (Takada et al. 2022), MEF cells (Cacquevel et al. 2012), and transgenic mice brains (Deyts et al. 2016). However, the specific molecular mechanism behind this phenomenon is not well understood. Because PS1 mutations induce significant cellular stress (Rojas‐Charry et al. 2020), Hsp90, with its co‐chaperone AHA1, plays a critical role in responding to this stress. We hypothesized that PS1 mutations might interact with Hsp90 as a client protein and that AHA1 could modulate this interaction. To test this, we used Chinese hamster ovary (CHO) cells stably overexpressing all human γ‐secretase components with FAD‐linked PS1 mutants (PS1‐ΔE9, PS1‐L166P, and PS1‐L286V). We also included non‐transfected (NT) parental CHO cells as a control. We found that the PS1‐L286V mutant increased the expression of endogenous APP, AHA1, and Hsp90 compared to WT (Figure 6A). However, the other PS1 mutants, ΔE9 and L166P, did not alter the expression of APP and AHA1 compared to WT. These findings suggest that AHA1 upregulation is not a universal feature of all FAD mutations but instead appears to be allele‐dependent and linked to the specific cellular stress environment associated with particular mutations, as several PS1 mutations are known to differentially alter calcium homeostasis, mitochondrial function, and oxidative stress (Guo et al. 1997; Sepulveda‐Falla et al. 2014). However, they did increase the expression of Hsp90 (Figure 6A). To gain further insight into whether increased endogenous APP expression in the PS1‐L286V mutant leads to more abnormal Aβ production than WT, we transiently transfected APP695 in WT and FAD‐linked PS1 mutants. We found that the PS1‐L286V mutant increased Aβ42 production three‐fold and increased the ratio of Aβ42/Aβ40 compared to WT (Figure 6B), consistent with previous reports (Hsu et al. 2020). On the other hand, PS1‐ΔE9 and PS1‐L166P mutations did not produce higher abnormal Aβ than WT. Still, the Aβ42/Aβ40 ratio increased compared to WT (Figure 6B). Additionally, we transfected all human γ‐secretase components, with either WT or FAD‐linked PS1 mutant E280A, in HEK293T cells. We found that the PS1‐E280A mutant increased AHA1 and endogenous APP expression compared to WT (Figure S5A). To further determine whether AHA1 directly regulates APP expression in the context of FAD‐linked PS1 mutations, we co‐transfected HEK293T cells with APP695 along with either WT or PS1‐E280A γ‐secretase components, followed by AHA1 knockdown using siRNA. As shown in Figure S5B, APP overexpression resulted in a marked increase in APP protein levels, which was further enhanced in PS1‐E280A–expressing cells compared to WT. Importantly, knockdown of AHA1 significantly reduced APP levels under both WT and PS1‐E280A conditions, despite exogenous APP overexpression. These findings suggest that specific FAD‐linked PS1 mutations can increase the expression of APP, AHA1, and Hsp90.

To investigate whether the increase in AHA1 expression caused by the FAD‐linked PS1 mutant L286V is necessary for the abnormal production of Aβ, we conducted a co‐transfection experiment of APP with either WT AHA1 or EV in CHO cells that stably express both WT and the PS1‐L286V mutant. The two‐way ANOVA and Tukey comparisons shown in Figure 6C indicated that the effects of AHA1 overexpression were not uniform across Aβ species and PS1 backgrounds. Accordingly, we do not conclude that AHA1 increased every Aβ measure in both backgrounds. Overall, these data support the conclusion that AHA1 expression can influence Aβ output in this CHO‐cell model.

## 4 Discussion

In this study, we found that the Hsp90 co‐chaperone AHA1 has a substantial influence on the production of Aβ. Our data indicate that AHA1 plays a crucial role in regulating the levels of Aβ by interacting with both APP and APH1. Specifically, AHA1 modulates APP abundance and influences the formation of the pre‐complex and mature γ‐secretase complex. Our data support a model in which APP or its C‐terminal fragment C99 functionally depends on AHA1 for post‐translational stability. Because APP folding and early maturation occur in the endoplasmic reticulum (ER), where molecular chaperone networks including Hsp90 and its co‐chaperones are highly active, AHA1 may regulate APP stability during early biosynthetic stages prior to γ‐secretase processing. While our results do not establish APP as a direct AHA1 client protein, the reduction of C99 levels following AHA1 depletion—even when C99 is expressed independently of APP processing—strongly supports a role for AHA1 in maintaining APP/C99 stability within a broader chaperone‐mediated proteostasis network. This modulation is associated with increased membrane‐associated fluorogenic substrate cleavage. Furthermore, we found that using an Hsp90‐binding‐defective AHA1 E67K mutant or a pharmacological inhibitor that blocks the AHA1/Hsp90 complex resulted in a reduction in both the pre‐complex and mature complex of γ‐secretase. Importantly, the changes in fluorogenic substrate cleavage observed in the biochemical assay paralleled the changes in assembled γ‐secretase complex abundance detected by BN‐PAGE. Because the assay was not normalized to assembled‐complex abundance, it does not establish enzymatic potency; instead, the altered signal likely reflects differences in the abundance of functional γ‐secretase complexes. Together, these findings support the idea that AHA1 contributes to γ‐secretase subunit stability and complex assembly, and that AHA1 modulates γ‐secretase, at least in part, through Hsp90‐dependent pathways. However, with the AHA1 E67K mutant or ND‐AHA1‐46 inhibitor, the expression of APP was increased compared to EV and DMSO. Similarly, direct inhibition of Hsp90 with 17‐AAG also increased APP expression and elevated Hsp70 and AHA1 levels, consistent with previous findings showing activation of the cellular heat shock response following Hsp90 inhibition (Holmes et al. 2008). In contrast, depletion of AHA1 reduced APP abundance, suggesting that the APP‐lowering effect of AHA1 knockdown is not simply a consequence of global Hsp90 inhibition. These findings further support the idea that AHA1 regulates APP stability through mechanisms that are at least partially independent of its interaction with Hsp90. Together, these findings support the idea that AHA1 can modulate APP levels through mechanisms that are at least partially independent of canonical Hsp90 interaction. Our findings are consistent with previous reports suggesting that AHA1 can function as a partially Hsp90‐independent autonomous chaperone. A previous study suggests that most AHA1 exists in cells as a self‐contained protein independent of high‐molecular‐weight Hsp90 protein complexes, and AHA1 itself can prevent the aggregation of denatured rhodanese and luciferase (Tripathi et al. 2014). A recent study found that Dicer1 is a client protein of AHA1, and AHA1 can influence the expression of Dicer1 independently of its interaction with Hsp90 (Liu et al. 2022). Another study suggests that SULT1A1 is a client of AHA1 but not of Hsp90 (Liu and Wang 2022). These findings indicate that AHA1 may have a role beyond its known function as a potent stimulator of Hsp90's ATPase activity. Importantly, independent large‐scale human proteomic studies support a role for AHA1 in AD pathology. A previous study identified AHA1 (AHSA1) as part of a proteostasis‐associated chaperone module that is significantly upregulated in AD brain and correlates with disease progression (Johnson et al. 2020). Similarly, another group reported increased AHA1 abundance within amyloid‐associated proteomic networks in AD brain tissue (Bai et al. 2021). These findings are consistent with our mechanistic data and suggest that dysregulation of AHA1 may contribute to proteostasis imbalance and altered APP processing in AD.

It is believed that targeting AHA1 could be a promising approach to reducing tau pathology in AD (Song et al. 2021; Wang et al. 2021). However, the role of AHA1 inhibitor in reducing γ‐secretase assembly formation was previously unknown. Our discovery revealed that the ND‐AHA1‐46 inhibitor significantly reduced the expression of the γ‐secretase components and formation of γ‐secretase complex. Notably, disruption of the AHA1/Hsp90 interaction reduced γ‐secretase assembly while increasing APP levels, suggesting that APP regulation is mechanistically distinct from AHA1/Hsp90‐dependent γ‐secretase assembly. The observed increase in APP may reflect reduced substrate processing and turnover secondary to impaired γ‐secretase assembly. The AHA1 inhibitor decreases tau aggregation and the formation of the γ‐secretase complex, showing potential for treating AD. Nevertheless, further research is needed to develop an inhibitor that can reduce tau and Aβ production without affecting the expression level of APP. It would also be important to show that other essential γ‐secretase substrates such as Notch1 are not unduly affected.

Importantly, because γ‐secretase also mediates Notch processing, the observed reduction in NICD levels following AHA1 knockdown and AHA1‐E67K expression raises potential concerns regarding Notch‐related liabilities. However, unlike classical γ‐secretase inhibitors, which directly block catalytic activity and previously failed in part because of Notch‐associated toxicities, AHA1 modulation appears to affect γ‐secretase assembly and homeostasis rather than direct enzymatic inhibition. Nevertheless, additional studies will be required to determine the extent to which downstream Notch signaling is altered and whether APP/Aβ‐related effects can be therapeutically separated from Notch‐associated pathways.

APP and PS1 autosomal dominant mutations are causative of FAD (Xu et al. 2016; Kelleher 3rd and Shen 2017). The upregulation of Hsp90 expression is a natural response to cellular stress, which includes responding to mutations. This upregulation helps to stabilize mutant proteins, allowing cells to maintain their proper function (Calderwood and Gong 2016; Whitesell and Lindquist 2005). In this regard, it was previously reported that cells carrying FAD‐linked APP‐I45F mutant increased the expression of Hsp90 (Pope et al. 2021). In this study, we used the APP C99 Iberian (I45F) mutation to show that it substantially increases Aβ42 production while decreasing Aβ40, resulting in a significant increase in the Aβ42/Aβ40 ratio, considered a crucial indicator of AD pathogenicity. Here, we also uncovered that APP‐C99‐I45F mutations increased AHA1, Hsp90, and APH1 levels. However, the levels of PS1‐CTF remained unchanged. Knockdown of AHA1 led to a decrease in C99, PS1‐CTF, and APH1 levels. As a result, there was a decrease in the Aβ42/Aβ40 ratio. Interestingly, APP C99‐I45F mutation increased APH1 levels, and recent genome‐wide association studies identified APH1 as an AD risk gene (Schwartzentruber et al. 2021). Furthermore, an upregulation of APH1 expression was seen in AD patients compared to the control group (Park et al. 2021). Previous studies reported that iPSC‐derived neurons carrying APP‐V717I mutation increased the levels of APP and tau (Muratore, Rice, et al. 2014). Consistent with previous observations that the APP‐V717I mutation elevates APP expression, we additionally found increased levels of Hsp90, AHA1, and PS1‐CTF together with altered Aβ generation. These observations extend the correlative findings to a disease‐relevant neuronal system; however, because AHA1 was not manipulated in the iPSC‐derived neurons, they do not establish AHA1‐mediated regulation in this model.

PS1 mutations have been associated with increased cellular stress, such as ER stress (Katayama et al. 2001) and oxidative stress (Schuessel et al. 2006). Previous studies have reported increased Hsp90 expression in fibroblasts derived from FAD patients carrying PS1‐A246E and PS1‐M146L mutations (Lopez‐Toledo et al. 2022). A recent study suggests that the PS1‐E280A mutation increases the levels of Hsp90 compared to sporadic AD in postmortem human brain samples (Almeida et al. 2024). Other studies suggest that human iPSC neurons expressing the PS1‐A246E mutant increase the levels of Hsp90, APP, and tau (Podvin et al. 2021; Hook et al. 2023). Moreover, iPSC‐derived neurons carrying PS1‐L150P and PS1‐E120K mutations increase APP and tau levels compared to control iPSC (Ochalek et al. 2017; Li et al. 2018). Furthermore, transgenic PS1‐M146V knock‐in mice showed increased levels of APP (Deyts et al. 2016). Other reports suggest that neuroblastoma N2A cells expressing the PS1‐P264S mutant exhibit increased APP levels (Takada et al. 2022). Despite increasing evidence, the molecular mechanism underlying the relationship between increased APP expression and FAD‐linked PS1 mutations remains unexplored. In this study, we reveal for the first time that AHA1 plays a role in regulating APP expression in FAD‐linked PS1 mutants. Our research indicates that the PS1‐L286V mutant increases CHO cells' endogenous APP, AHA1, and Hsp90. Furthermore, the PS1‐L286V mutant increases the abnormal Aβ production compared to the WT. As reflected by the two‐way ANOVA and Tukey comparisons in Figure 6C, WT AHA1 overexpression altered Aβ output, although the effects were not uniform across Aβ species and PS1 backgrounds. Additionally, we found that the PS1‐E280A mutation increases levels of both APP and AHA1. Several FAD mutants have been discovered to increase the Aβ42/Aβ40 ratio, leading to early amyloid deposition in the brain (Jankowsky et al. 2004; Borchelt et al. 1997; Piccini et al. 2007; Scheuner et al. 1996; Tanzi and Bertram 2005). Therefore, further research is needed to investigate the connection between the upregulation of AHA1/Hsp90 and the abnormal production of Aβ and accumulation of tau in FAD.

While our data consistently demonstrate that AHA1 regulates APP protein abundance, γ‐secretase assembly, and Aβ production, some mechanistic questions remain. Although APP mRNA levels were not significantly changed upon AHA1 knockdown, we did not determine whether AHA1 influences APP translation, folding, trafficking, or degradation, and similar analyses were not performed for all γ‐secretase components. We also did not define the specific interaction domains through which AHA1 engages APP or APH1, which would clarify the structural basis of AHA1‐driven assembly. Finally, our conclusions are based on cellular models, and future studies using AHA1 knockout or tissue‐specific AHA1 deletion mice would provide valuable insight into the role of AHA1 in AD progression, although a fully characterized AHA1‐null mouse line has not been widely reported.

Together, our research has shown that AHA1 plays a crucial role in the production of Aβ. We have discovered that AHA1 regulates the expression of APP and the assembly of γ‐secretase. By disrupting the complex formation between Hsp90 and AHA1, we can reduce the assembly of γ‐secretase. Additionally, we observed that AHA1 is up‐regulated and stimulates the expression of APP or APH1 under FAD‐linked mutations, indicating a molecular connection between AHA1/Hsp90 and FAD‐linked APP and PS1 mutations. Our findings suggest that targeting AHA1 could be a promising approach for developing treatments that control Aβ levels, which are critical in AD. Our findings may offer significant implications for the development of new AD treatments.

## Author Contributions

**Sadequl Islam:** writing – original draft, data curation, investigation. **Michael S. Wolfe:** conceptualization, formal analysis, supervision, writing – review and editing, funding acquisition, project administration, validation. **Arshad Ali Noorani:** conceptualization, methodology, data curation, investigation, writing – original draft, formal analysis. **Vaishnavi Nagarajan:** data curation. **Emma Filip:** investigation, conceptualization, writing – original draft. **Kevin Catalfano:** data curation. **Kun Zou:** writing – review and editing, formal analysis, validation. **Shravan Paswan:** data curation. **Brian S. J. Blagg:** formal analysis, writing – review and editing, validation. **Heather M. Wilkins:** data curation.

## Funding

This work was supported by grant R01 AG066986 from the National Institute on Aging (NIA), National Institutes of Health to Michael S. Wolfe.

## Conflicts of Interest

The authors declare no conflicts of interest.

## Supporting information


**Data S1:** jnc70544‐sup‐0001‐Supinfo1.zip.

**Figure S1:** ND‐AHA1‐46 inhibits interaction between Hsp90 and AHA1.
**Figure S2:** ND‐AHA1‐46 reduces γ‐secretase complex formation.
**Figure S3:** Knockdown of AHA1 decreases γ‐secretase complex formation.
**Figure S4:** APP‐V717I mutation increases the levels of AHA1, Hsp90, APP, PS1‐NTF, and γ‐secretase complex.
**Figure S5:** PS1‐E280A mutation increases the levels of AHA1 and APP.
**Figure S6:** Normalization of Aβ production to APP and C99 levels.
**Figure S7:** Hsp90 does not associate with APP.

**Data S2:** jnc70544‐sup‐0003‐Supinfo2.xlsx.

## Data Availability

The data supporting the findings of this study are available from the corresponding author upon reasonable request. All relevant data are included within the article and its Supporting Information files.
