# Supplementary material for: AHA1 Regulates Aβ Production via Modulation of APP Abundance and γ‐Secretase Assembly
Source: J Neurochem. 2026 Aug 27;170(9):e70544. doi: 10.1111/jnc.70544 (PMC13522734; doi:10.1111/jnc.70544)
Supplement: Supplementary file 1 [file JNC-170-e70544-s001.pdf]

SUPPLEMENTARY MATERIAL

AHA1 regulates Aβ production via modulation of APP abundance and γ-secretase assembly

Arshad Ali Noorani[1,2]*, Sadequl Islam[3], Emma Filip[4], Kevin Catalfano[4], Vaishnavi Nagarajan[5], Shravan Paswan[2], Heather M. Wilkins[6], Brian S. J. Blagg[4], Kun Zou[3] and Michael S. Wolfe[1]*

[1]Department of Medicinal Chemistry, The University of Kansas, Lawrence, Kansas 66045, USA.
[2]Department of Pharmacology & Immunology, Medical University of South Carolina, Charleston, South Carolina 29425, USA.
[3]Department of Neuro-oncology, Institute of Brain Science, Graduate School of Medical Sciences, Nagoya City University, 1-Kawasumi, Mizuho-cho, Mizuho-ku, Nagoya 467-8601, Japan.
[4]Department of Chemistry and Biochemistry, University of Notre Dame, Notre Dame, Indiana 46556, USA.
[5]School of Natural Sciences (Biology), Northwest Missouri State University, Maryville, MO, USA.
[6]University of Kansas Alzheimer's Disease Research Center, University of Kansas Medical Center, Kansas City, Kansas, USA.

***To whom correspondence should be addressed:**

Arshad Ali Noorani
Department of Pharmacology & Immunology,
Medical University of South Carolina, Charleston, SC 29425, USA.
Email: noorani@musc.edu;

and

Michael S. Wolfe
Department of Medicinal Chemistry,
University of Kansas, Lawrence, KS 66045, USA.
Email: mswolfe@ku.edu

Running title: AHA1 regulates γ-secretase and APP protein levels

Supplementary Figure Legends

Figure S1. ND-AHA1-46 inhibits interaction between Hsp90 and AHA1. (A) Chemical structure of ND-AHA1-46. The 1,5-bisubstituted-1,2,3,4-tetrazole containing AHA1/Hsp90 disruptor ND-AHA1-46. **(B)** Immunoprecipitated AHA1 from HEK293T cells treated with DMSO or ND-AHA1-46 10 μM for 24 h was analyzed by Western blotting.

Figure S2. ND-AHA1-46 reduces γ-secretase complex formation. (A) The expression levels of full-length APP, PS1-NTF, NCT, and APH1 in HEK-APP cells after treatment of DMSO or ND-AHA1-46 10 μM were detected with Western blotting. **(B)** 10 μg of protein from DMSO or ND-AHA1-46 treated cells were subjected to BN-PAGE or SDS-PAGE and analyzed with Western blotting using the indicated antibodies. **(C)** PS-DKO MEF cells treated with DMSO or ND-AHA1-46 10 μM for 24 h. 10 μg of protein from DMSO or ND-AHA1-46 treated cells were subjected to BN-PAGE and analyzed with Western blotting using the APH1 antibody. Data are presented as mean ± SEM (n = 3 independent cell culture preparations). Statistical significance was determined using a two-tailed Student's t-test for comparisons between two groups. **, $p < 0.01$; ***, $p < 0.001$.

Figure S3. Knockdown of AHA1 decreases γ-secretase complex formation. (A) 10 μg of protein from WT MEF cells after knockdown of AHA1 were subjected to BN-PAGE or SDS-PAGE and analyzed with Western blotting using the indicated antibodies. Data are presented as mean ± SEM (n = 3 independent cell culture preparations). Statistical significance was determined using a two-tailed Student's t-test for comparisons between two groups. **, $p < 0.01$.

Figure S4. APP-V717I mutation increases the levels of AHA1, Hsp90, APP, PS1-NTF, and γ-secretase complex. (A) The amount of secreted Aβ40 and Aβ42 levels in the media of iPSC-derived neurons with WT or APP-V717I mutation were measured with an Aβ ELISA kit. **(B)** The expression levels of AHA1, Hsp90, full-length APP, and PS1-NTF in iPSC-derived neurons with WT or APP-V717I mutation were detected with Western blotting. **(C)** 10 μg of protein from WT or APP-V717I cells was subjected to BN-PAGE and analyzed with Western blotting using PS1-NTF antibody. Data are presented as mean ± SEM (n = 3 independent cell culture preparations). Statistical significance was determined using a two-tailed Student's t-test for comparisons between two groups. **, $p < 0.01$; ***, $p < 0.001$; NS, not significant.

Figure S5. PS1-E280A mutation increases the levels of AHA1 and APP. (A) The expression levels of AHA1, full-length APP, PS1-NTF, NCT, and PEN2 were assessed by Western blotting in HEK293T cells after transfection of overexpressing WT or PS1-E280A. **(B)** HEK293T cells were co-transfected with APP695 and either WT or PS1-E280A constructs, followed by siRNA-mediated knockdown of AHA1. The expression levels of full-length APP and AHA1 were assessed by Western blotting. Data are presented as mean ± SEM (n = 3 independent cell culture preparations). Statistical significance was determined using a two-tailed Student's t-test for comparisons between

two groups and two-way ANOVA followed by Tukey's multiple-comparison test for panel B. **p < 0.01; ***p < 0.001.

Figure S6. Normalization of Aβ production to APP and C99 levels. (A) Aβ40 and Aβ42 levels normalized to full-length APP (Aβ40/APP and Aβ42/APP) in HEK-APP cells following AHA1 knockdown. **(B)** Aβ40 and Aβ42 levels normalized to C99 (Aβ40/C99 and Aβ42/C99) in HEK293T cells co-expressing AHA1 knockdown and either APP C99 or APP C99-I45F. Data are presented as mean ± SEM (n = 3 independent cell culture preparations). Statistical significance was determined using a two-tailed Student's t-test for comparisons between two groups and two-way ANOVA followed by Tukey's multiple-comparison test for panel B. *p < 0.05; **p < 0.01; ***p < 0.001.

Figure S7. Hsp90 does not associate with APP. (A) Co-IP of APP and Hsp90 in HEK293T cells expressing APP695 following 24 h treatment with 17-AAG (1 μM). No detectable APP–Hsp90 interaction was observed under these experimental conditions. Data are obtained from three independent biological experiments (independent cell culture preparations).

Noorani et. al.,
Fig. S1.

A

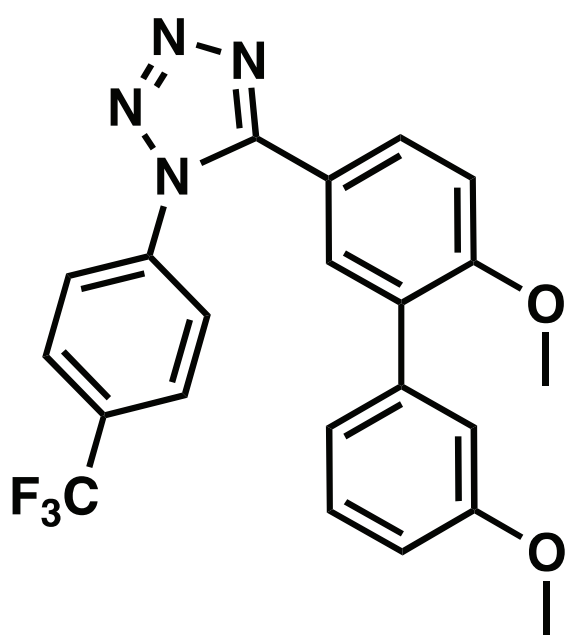

ND-AHA-46

B

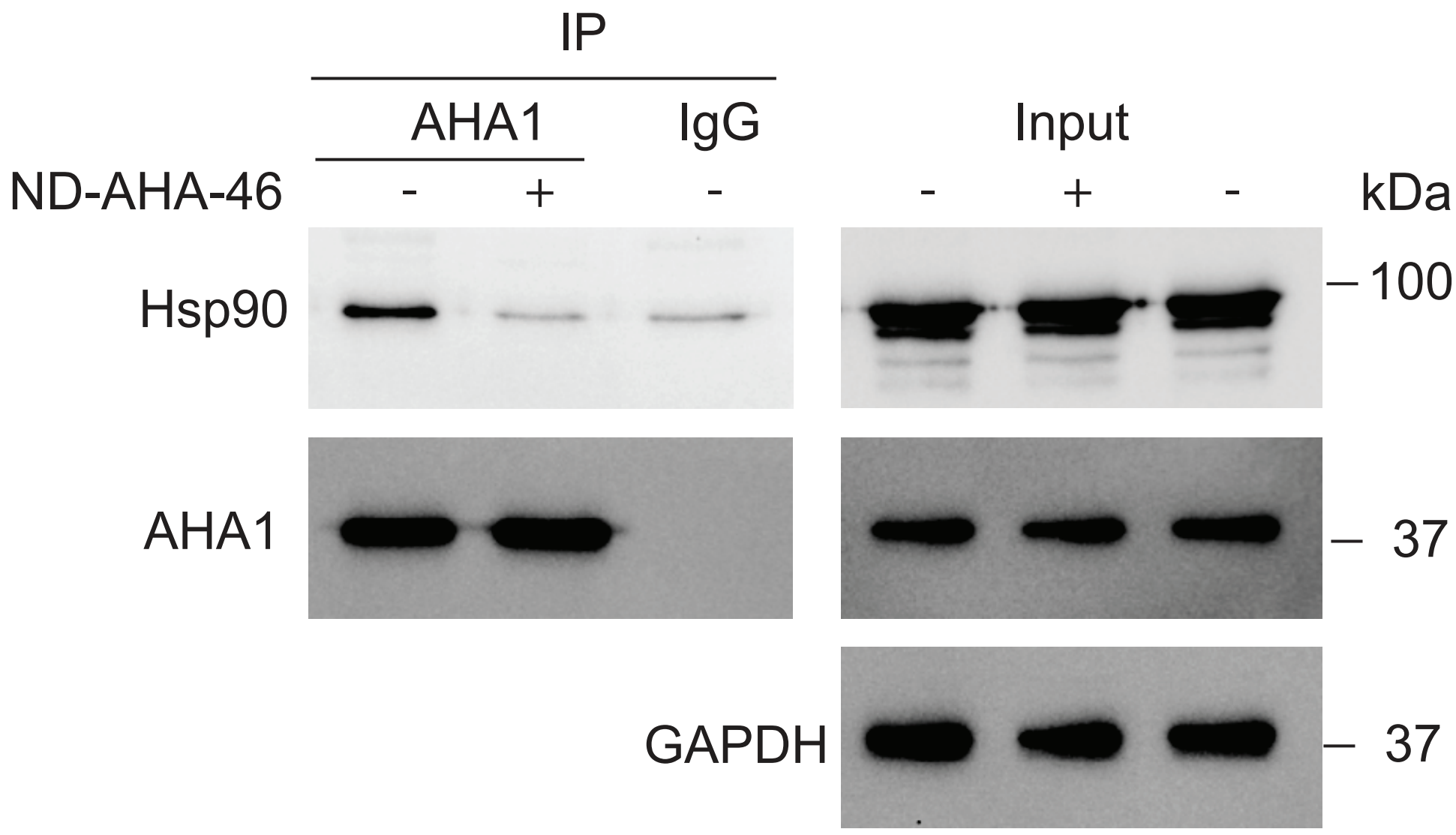

Noorani et. al.,
Fig. S2.

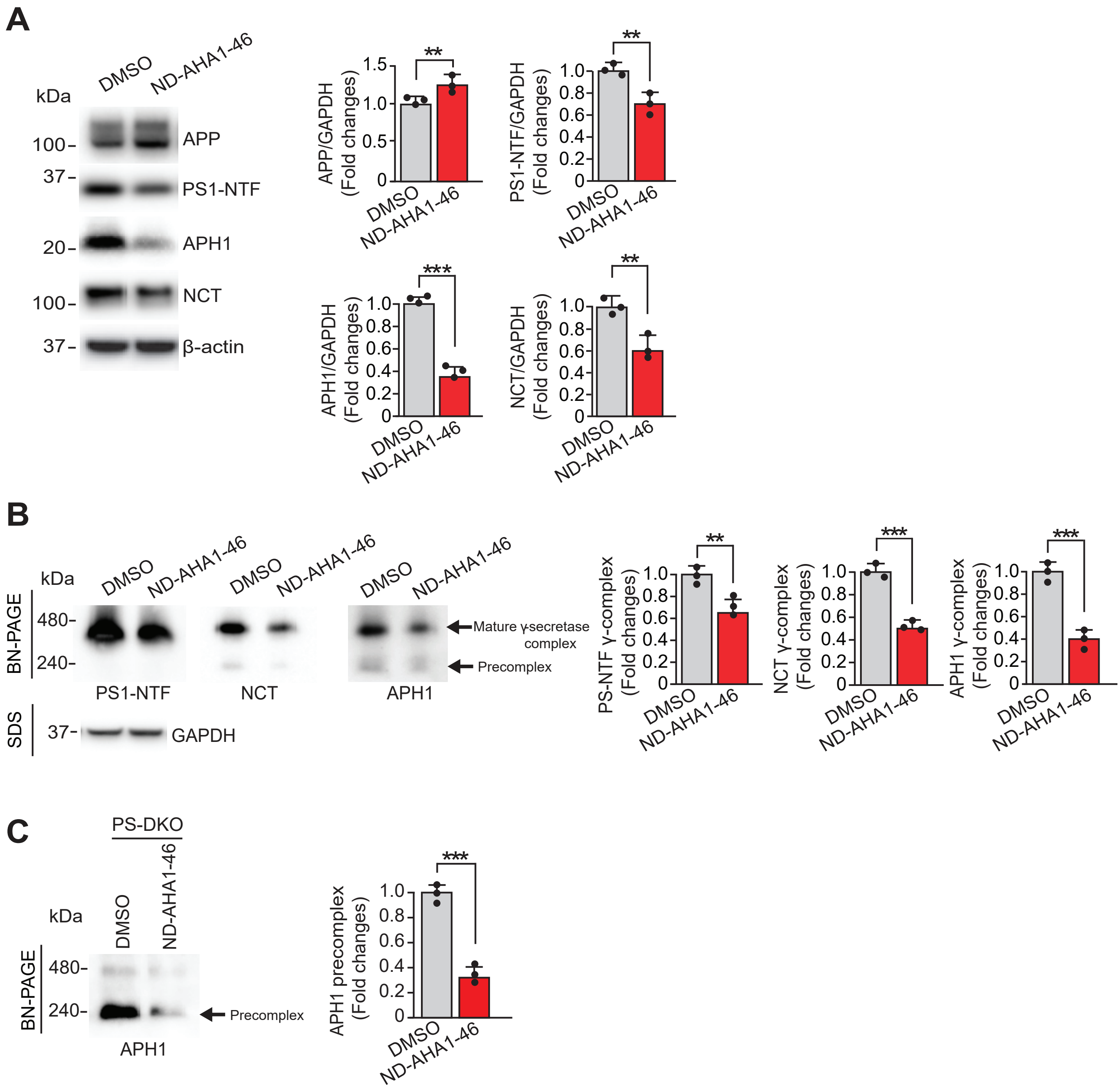

Noorani et. al.,
Fig. S3.

A

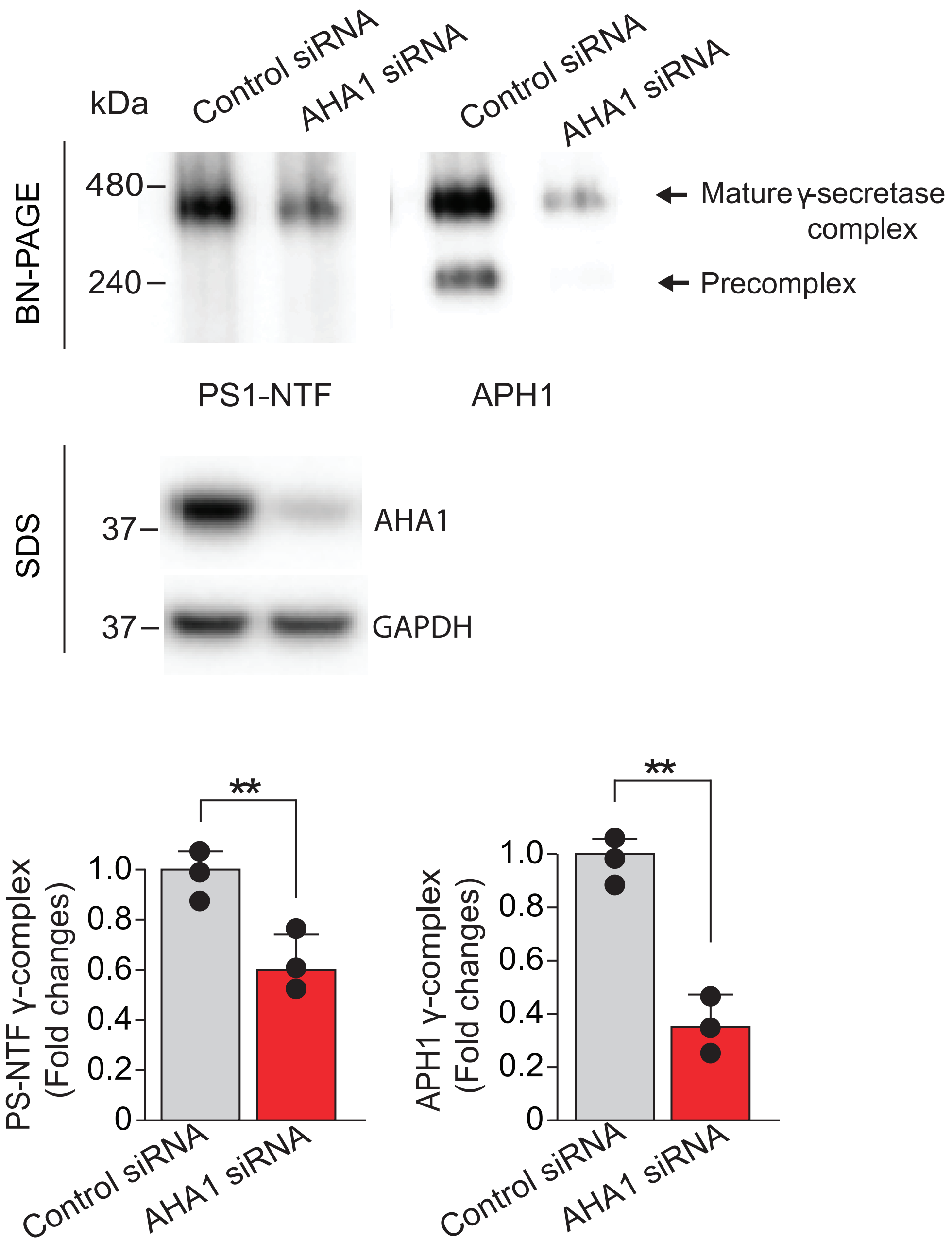

Noorani et. al.,
Fig. S4.

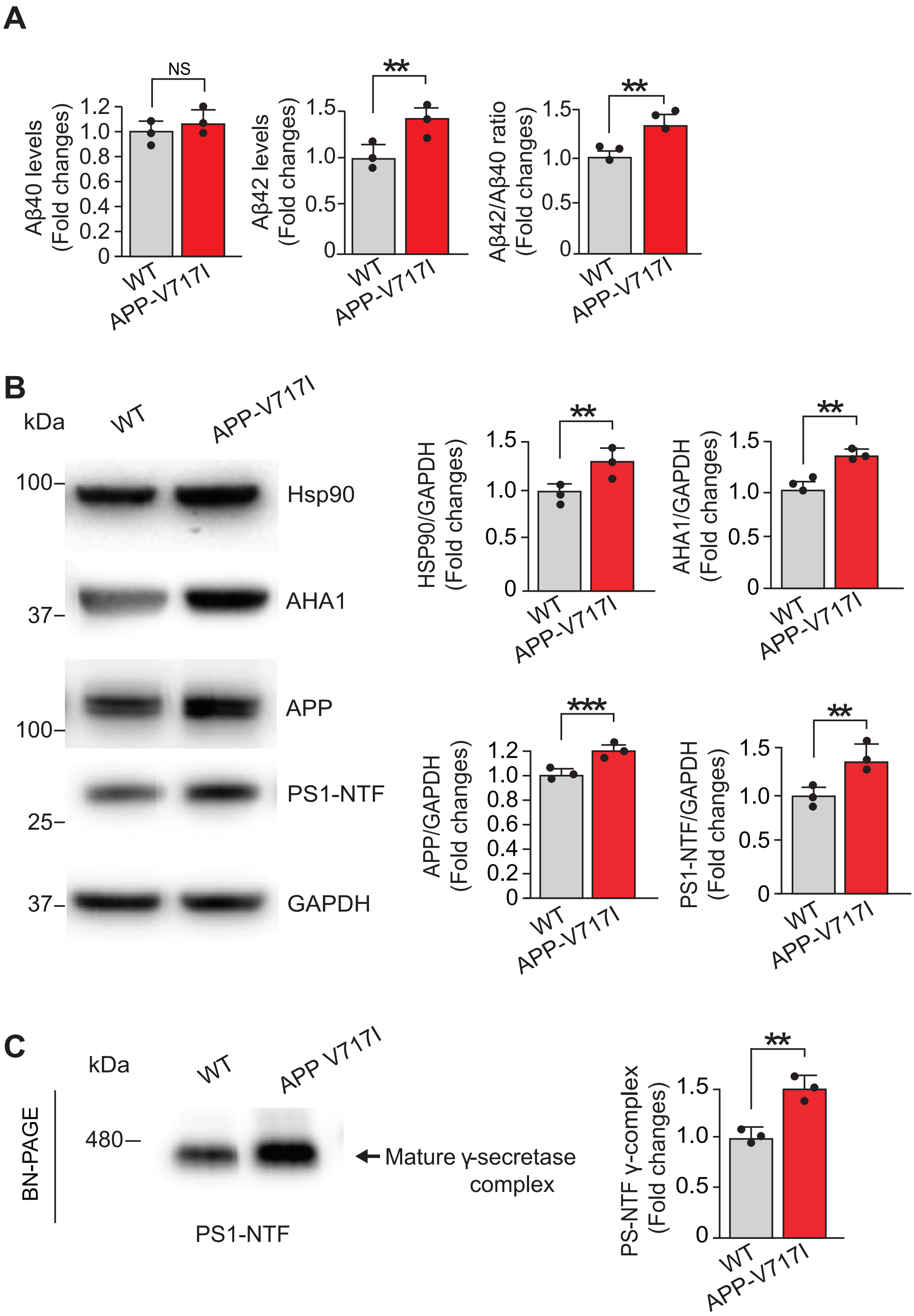

Noorani et. al.,
Fig. S5.

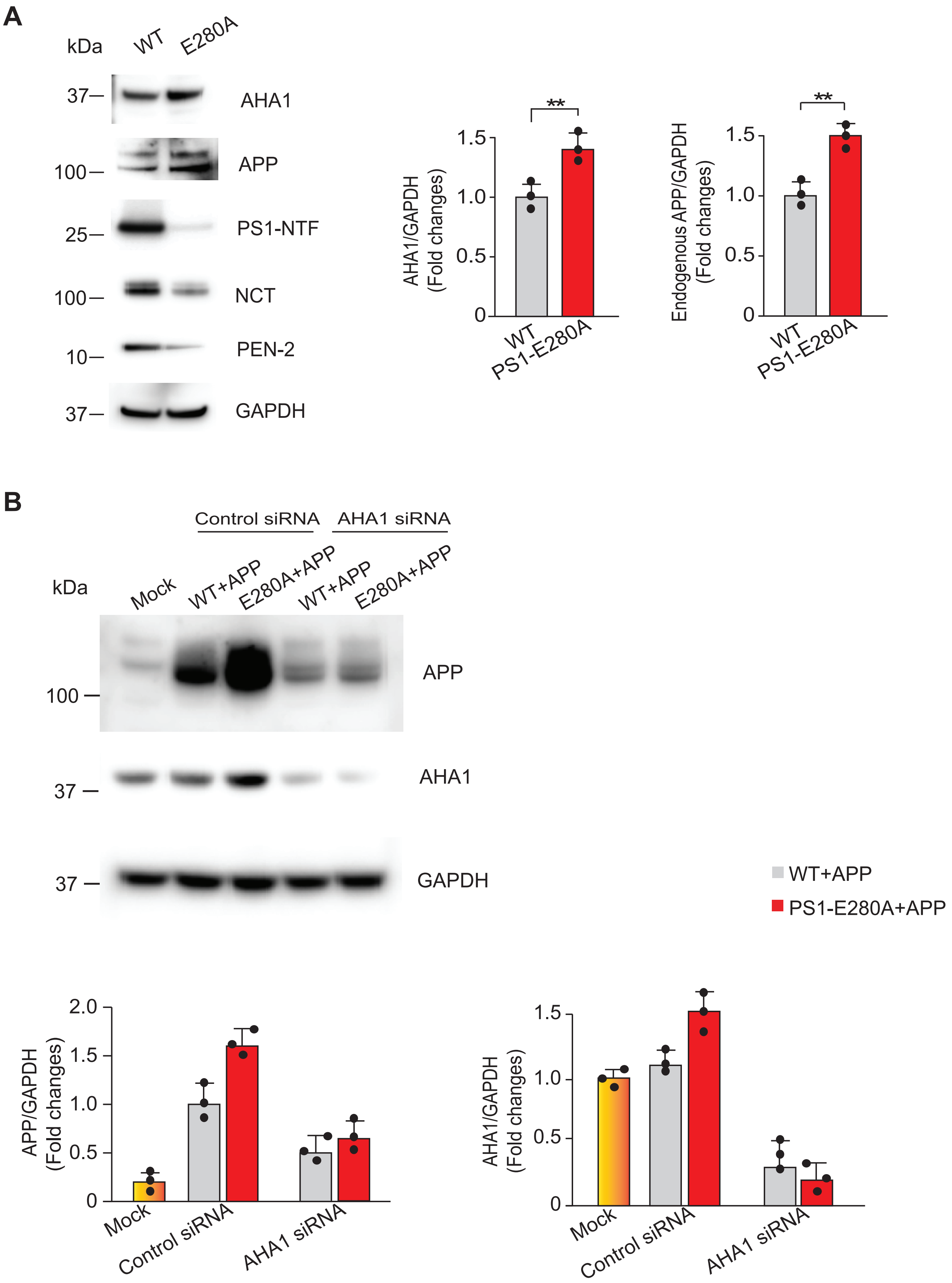

Noorani et. al.,
Fig. S6.

A

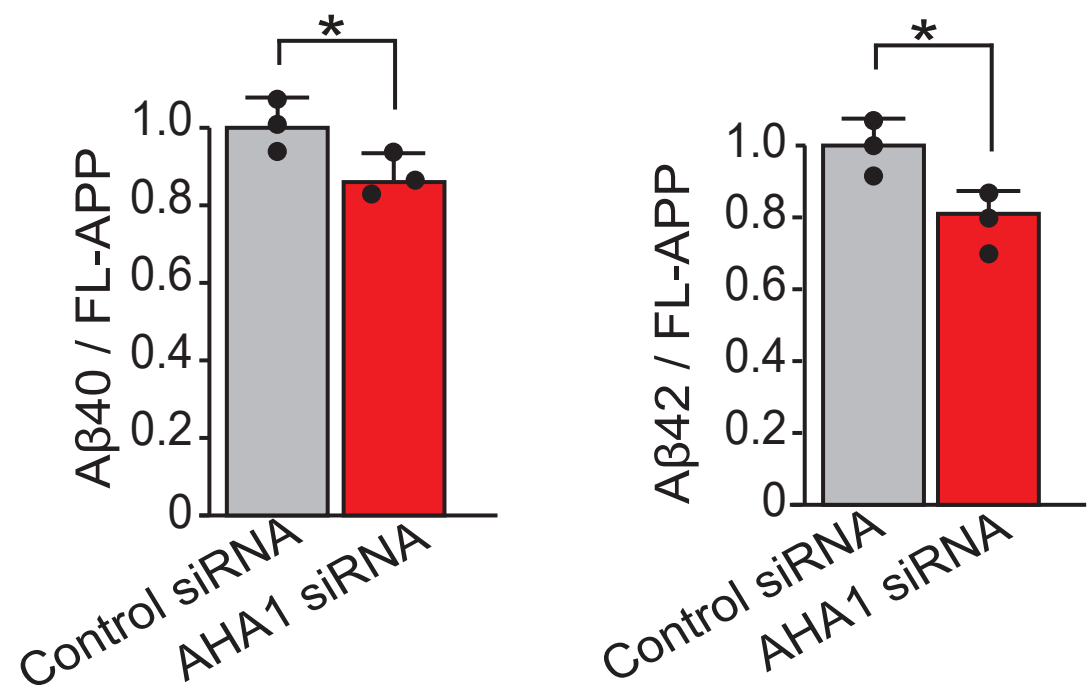

B

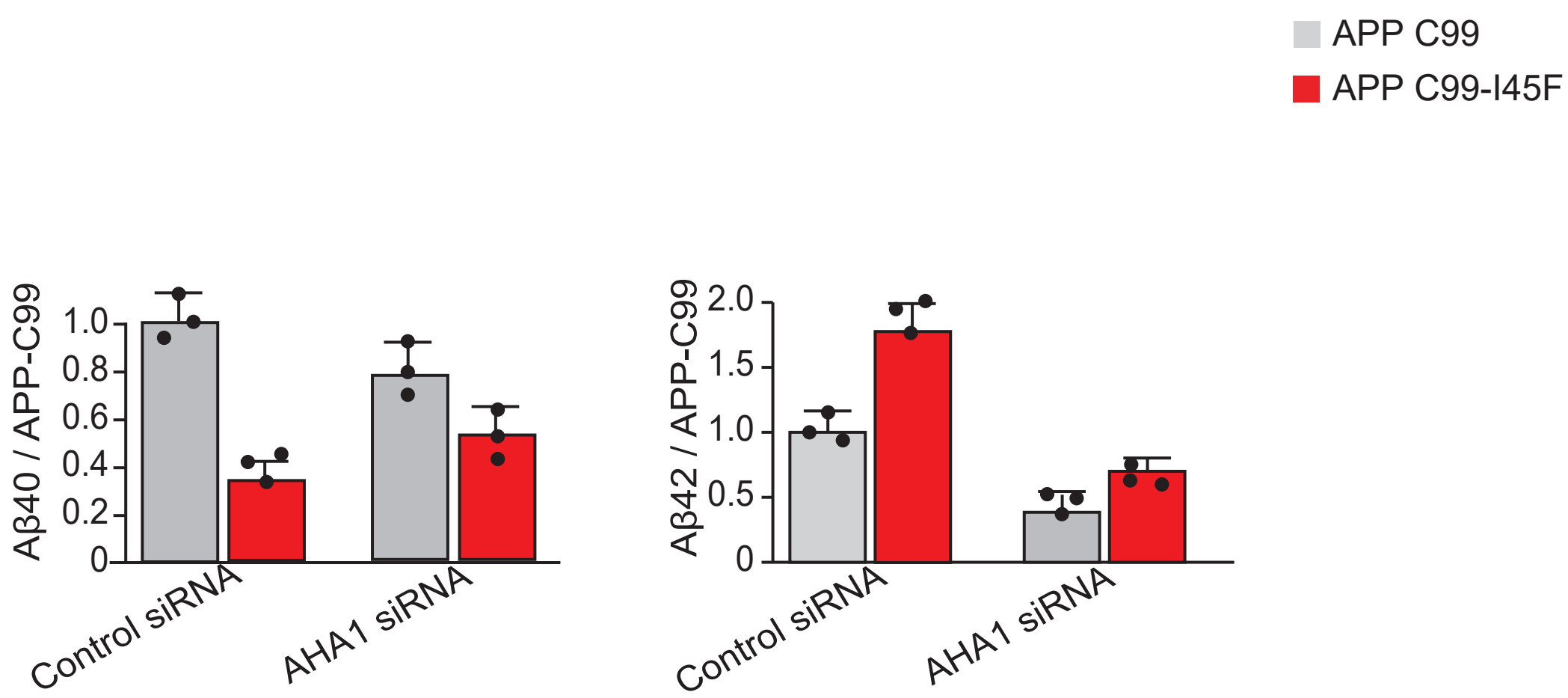

Noorani et. al.,
Fig. S7.

A

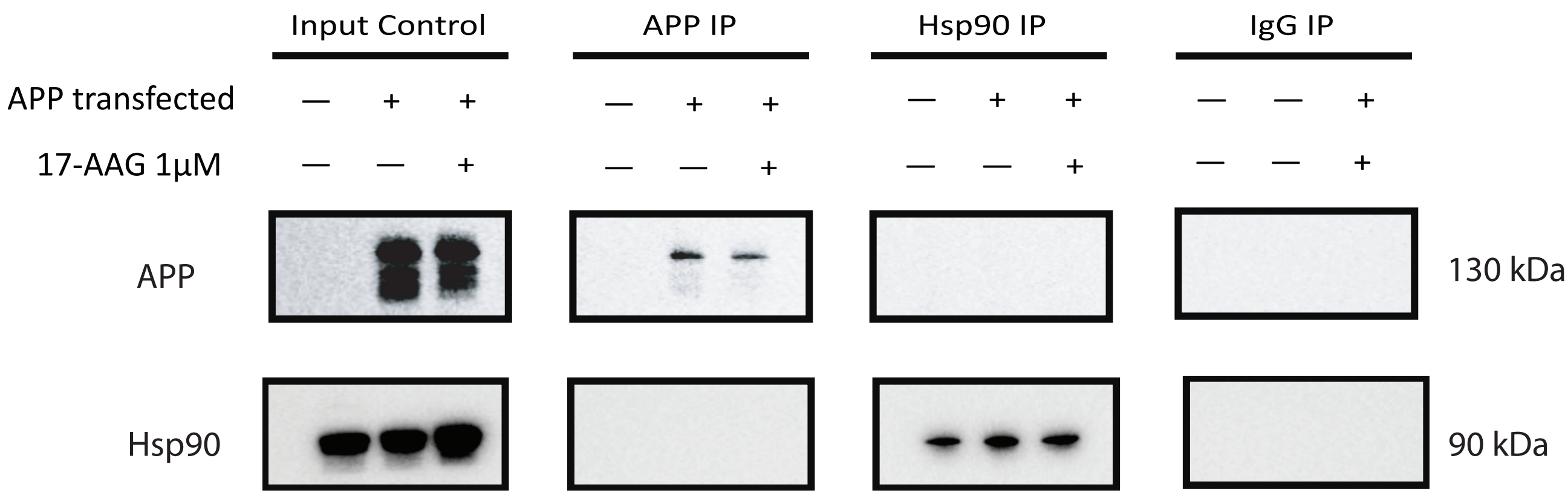

Original Western Blot Membranes

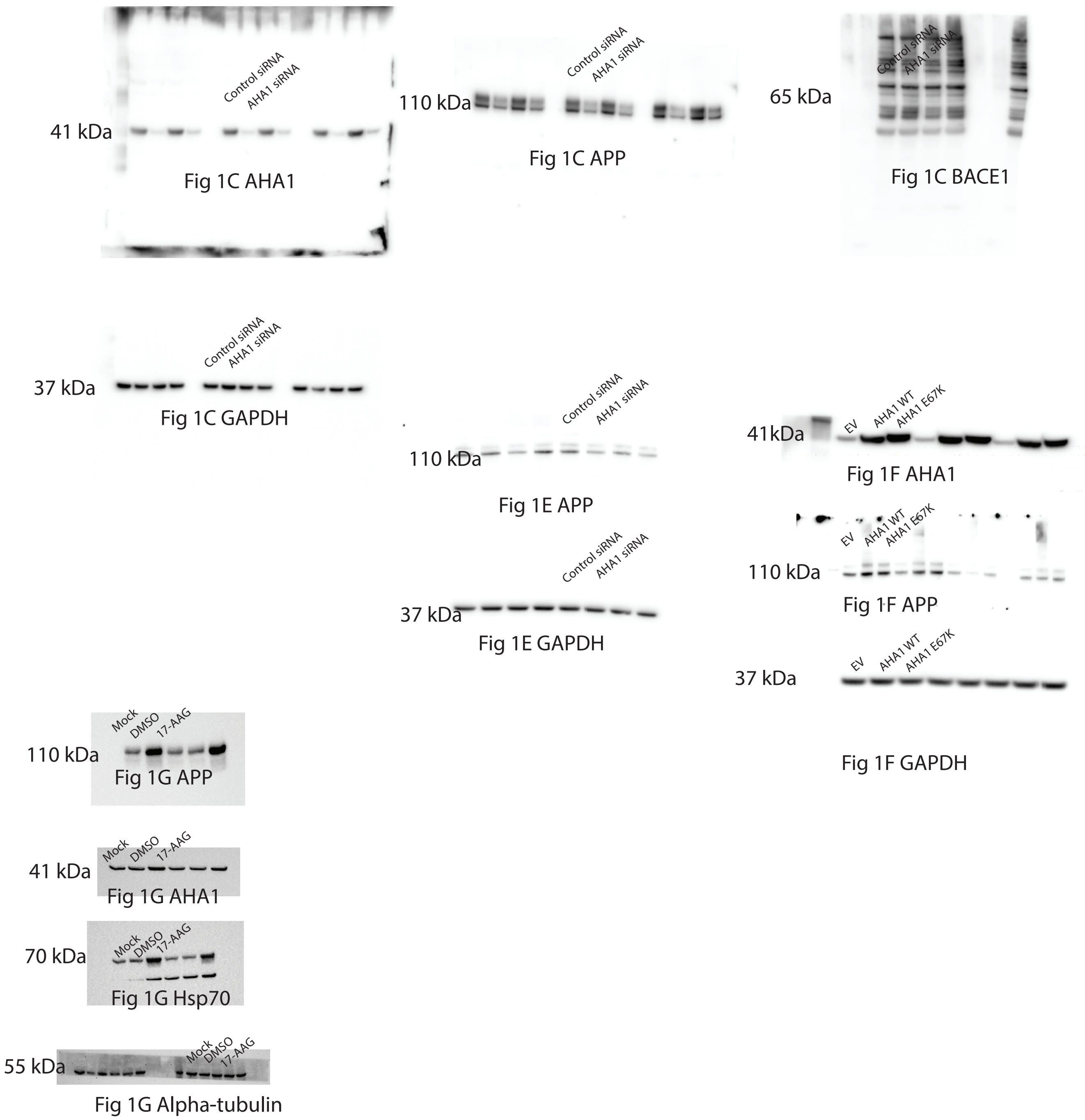

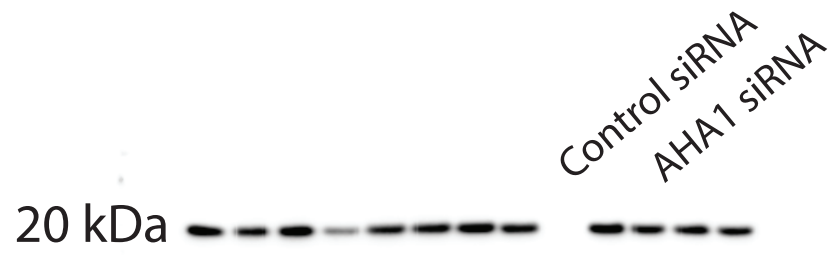

Fig 2A PS1-CTF

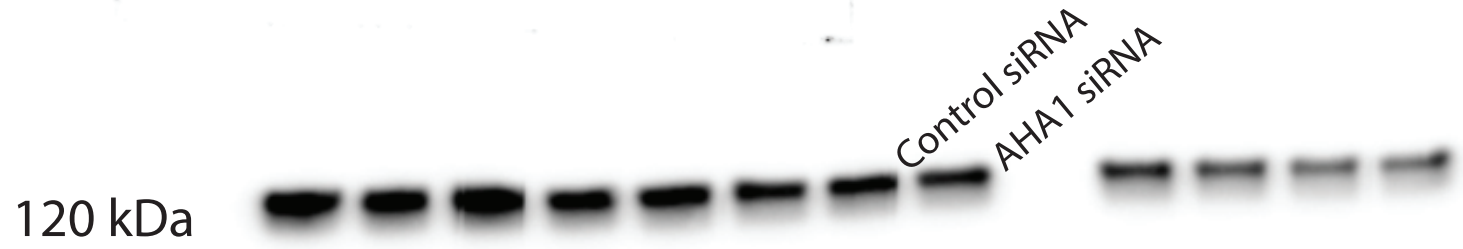

Fig 2A NCT

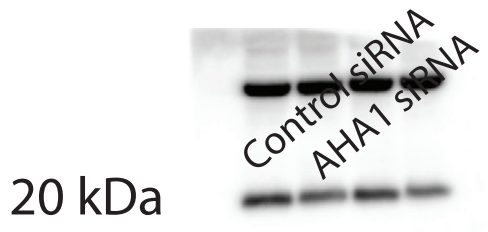

Fig 2A APH1

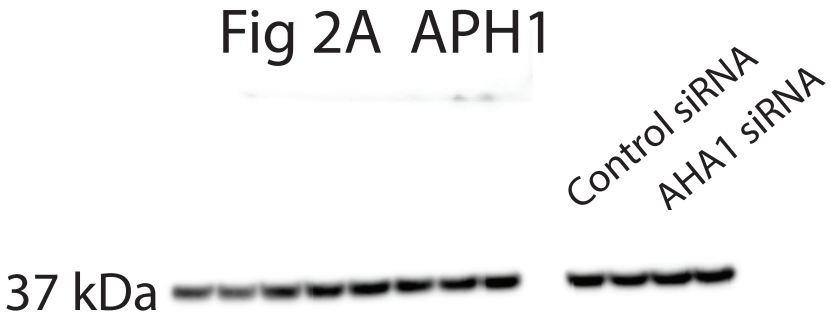

Fig 2A beta-actin

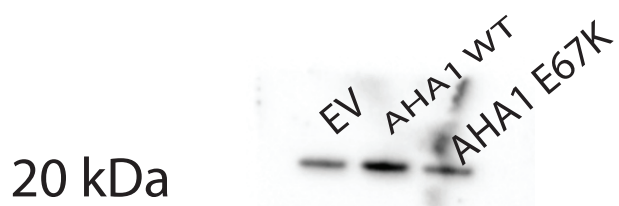

Fig 2B PS1-CTF

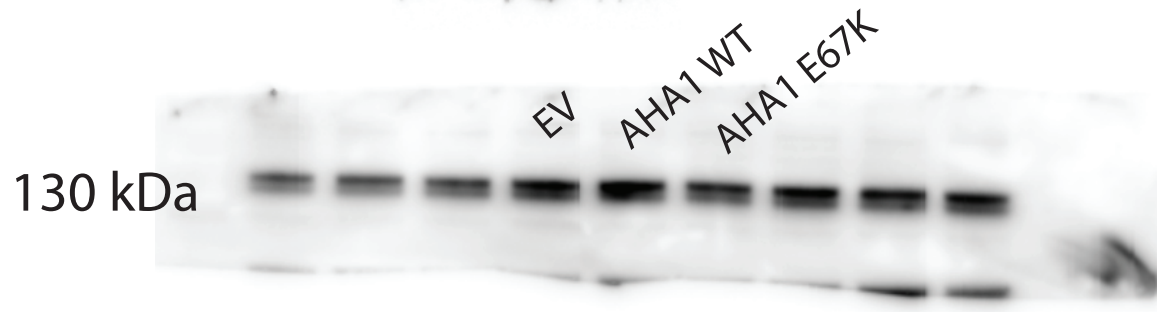

Fig 2B NCT

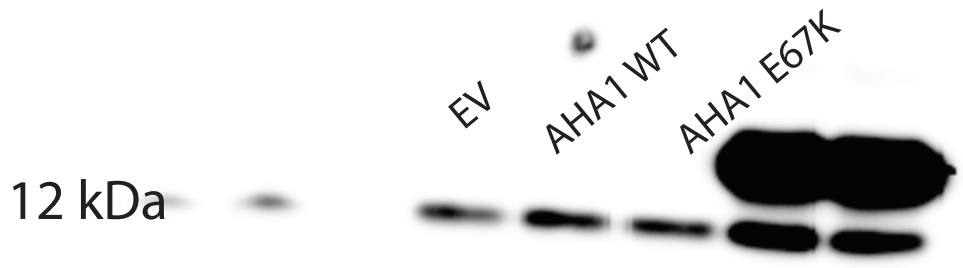

Fig 2B PEN2

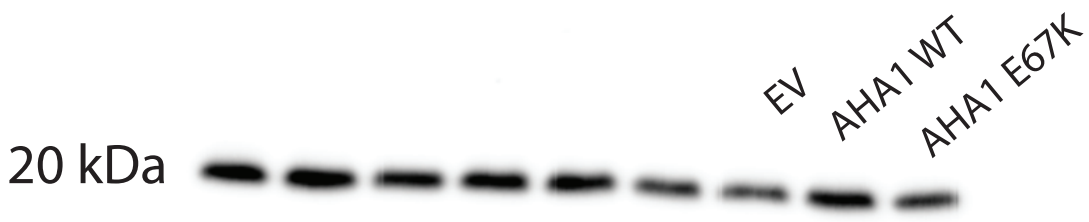

Fig 2B APH1

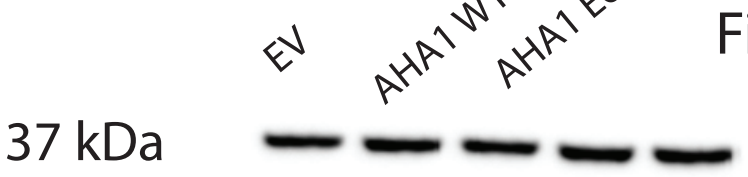

Fig 2B GAPDH

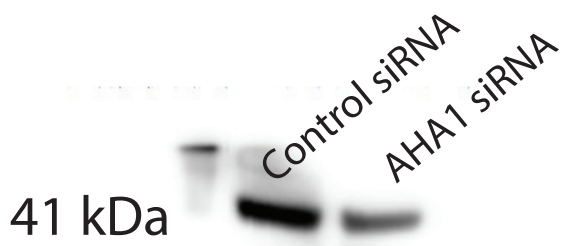

Fig 2E AHA1

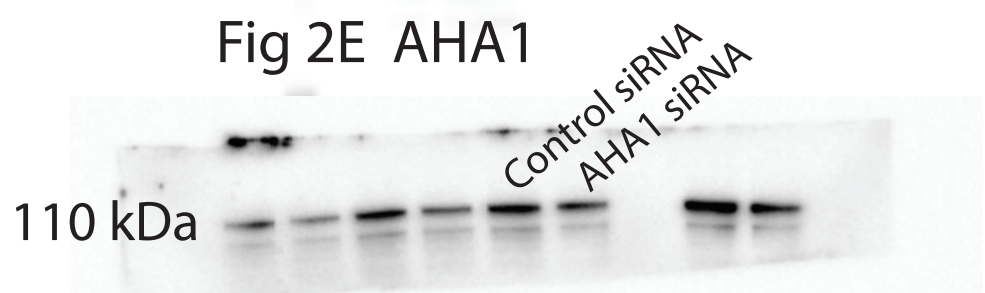

Fig 2E NICD

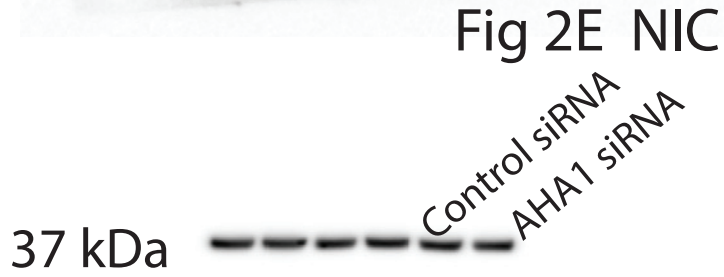

Fig 2E GAPDH

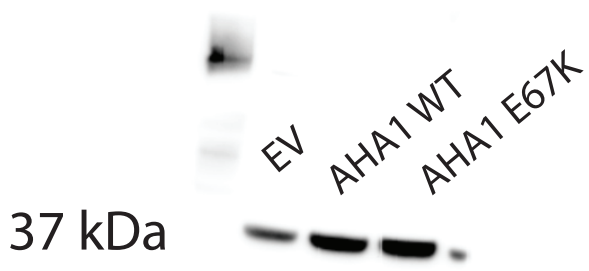

Fig 2F AHA1

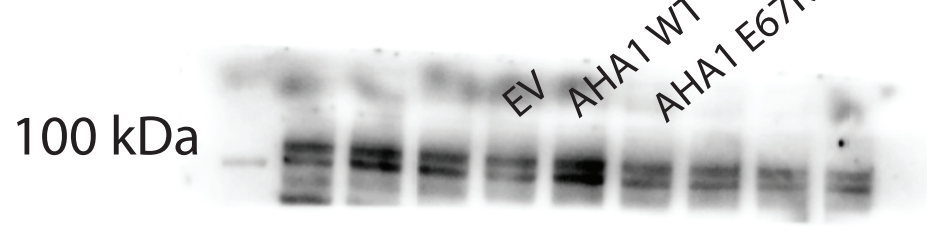

Fig 2F NICD

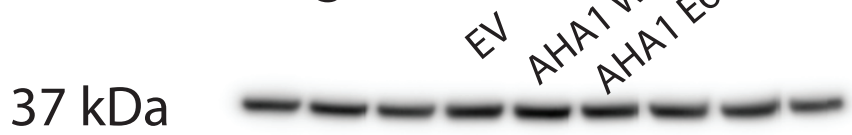

Fig 2F GAPDH

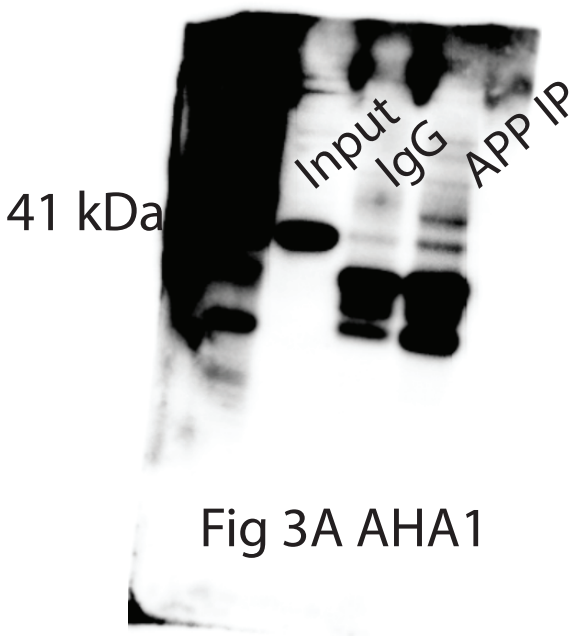

Fig 3A AHA1

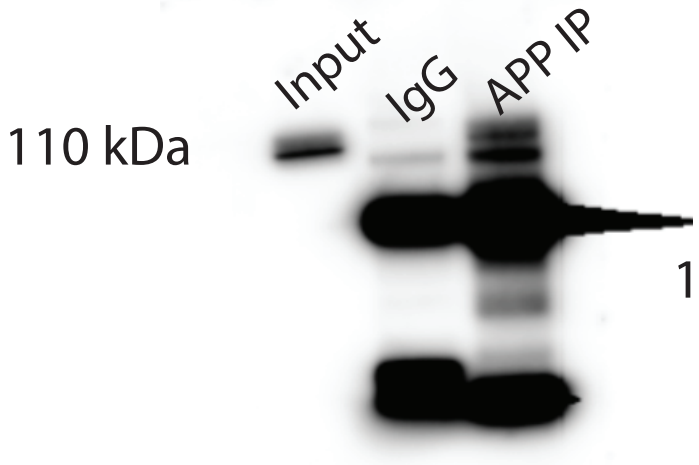

Fig 3A APP

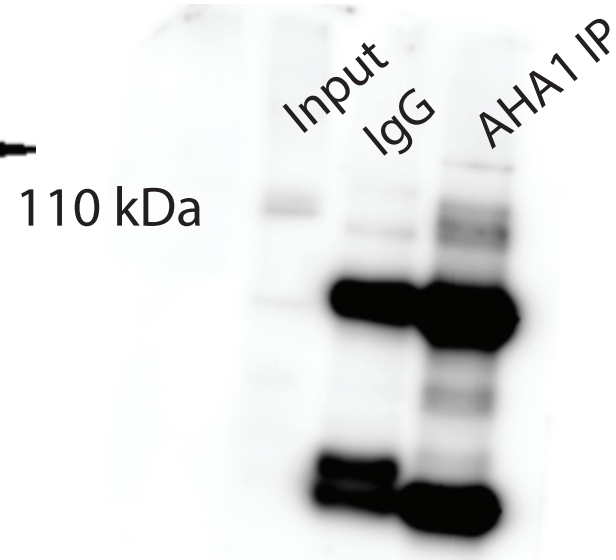

Fig 3A APP

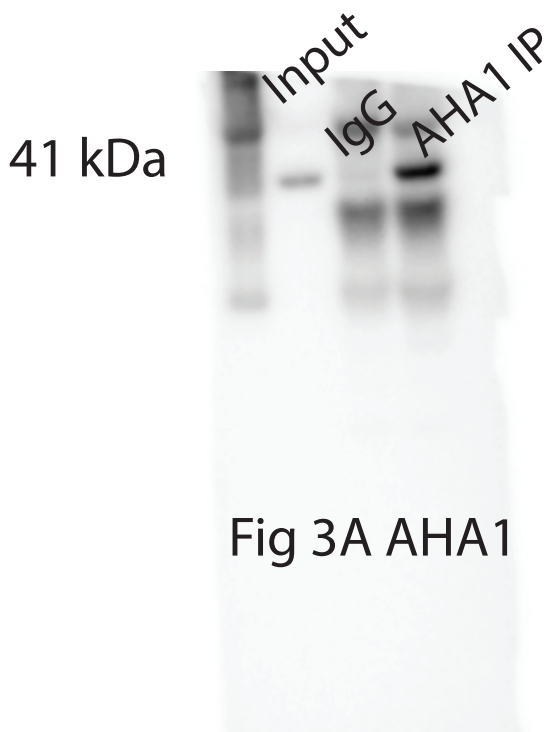

Fig 3A AHA1

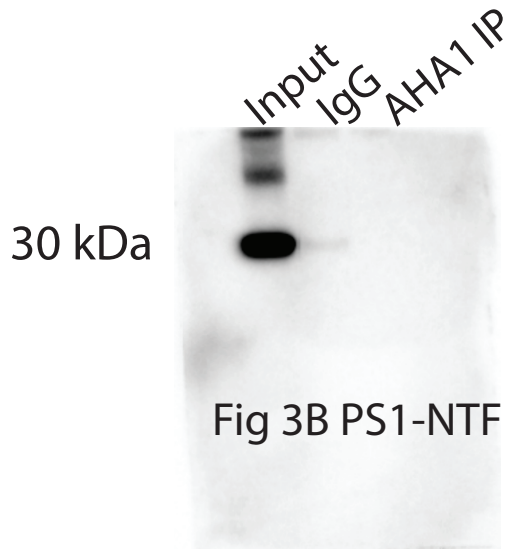

Fig 3B PS1-NTF

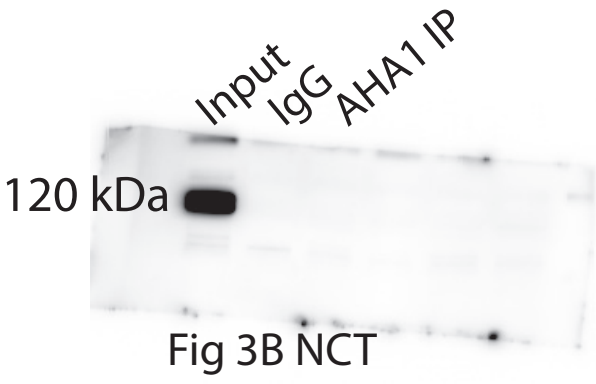

Fig 3B NCT

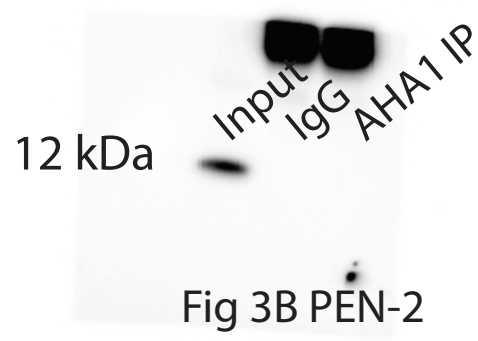

Fig 3B PEN-2

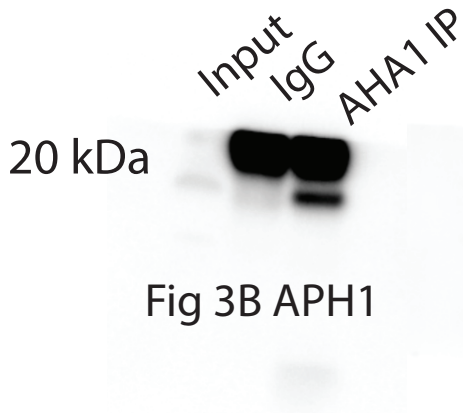

Fig 3B APH1

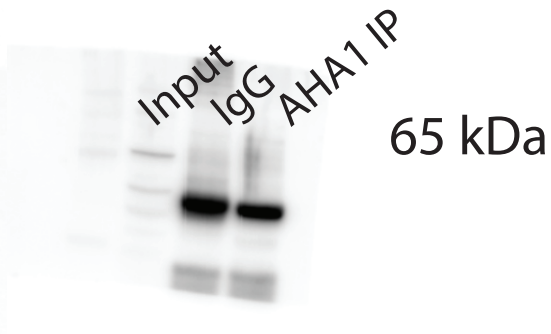

Fig 3B BACE1

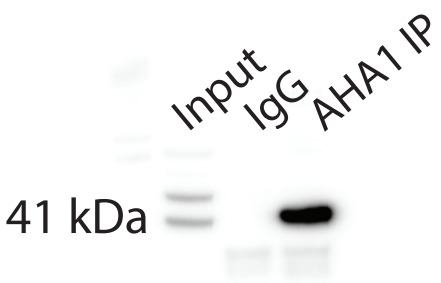

Fig 3B AHA1

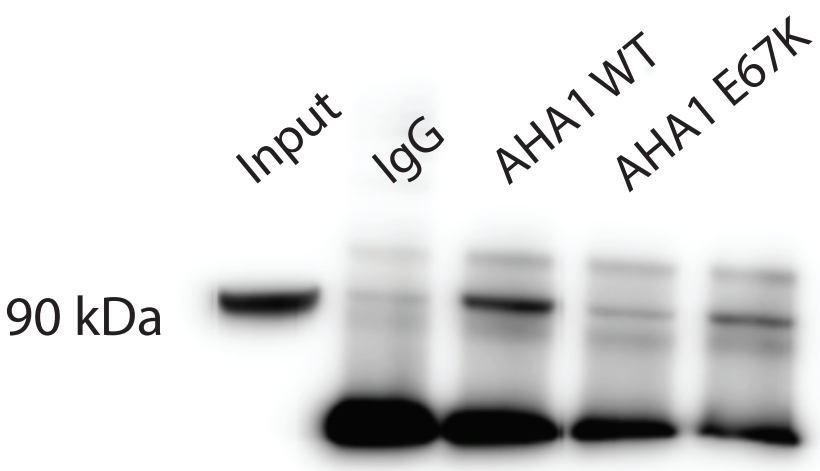

Fig 3C Hsp90

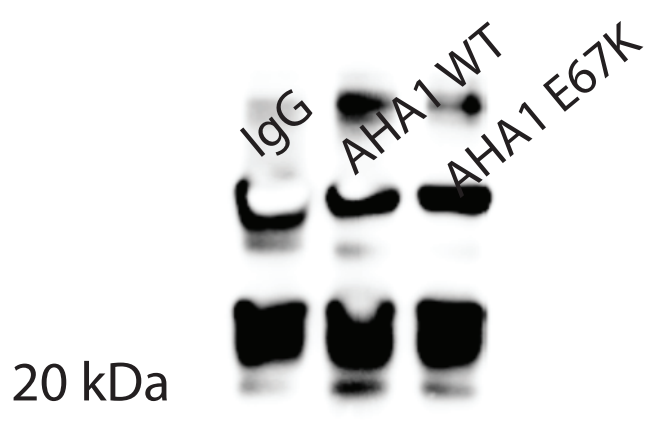

Fig 3C APH1

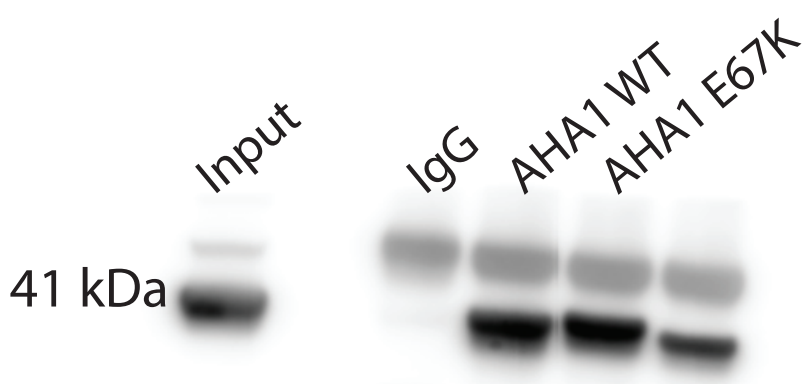

Fig 3C AHA1

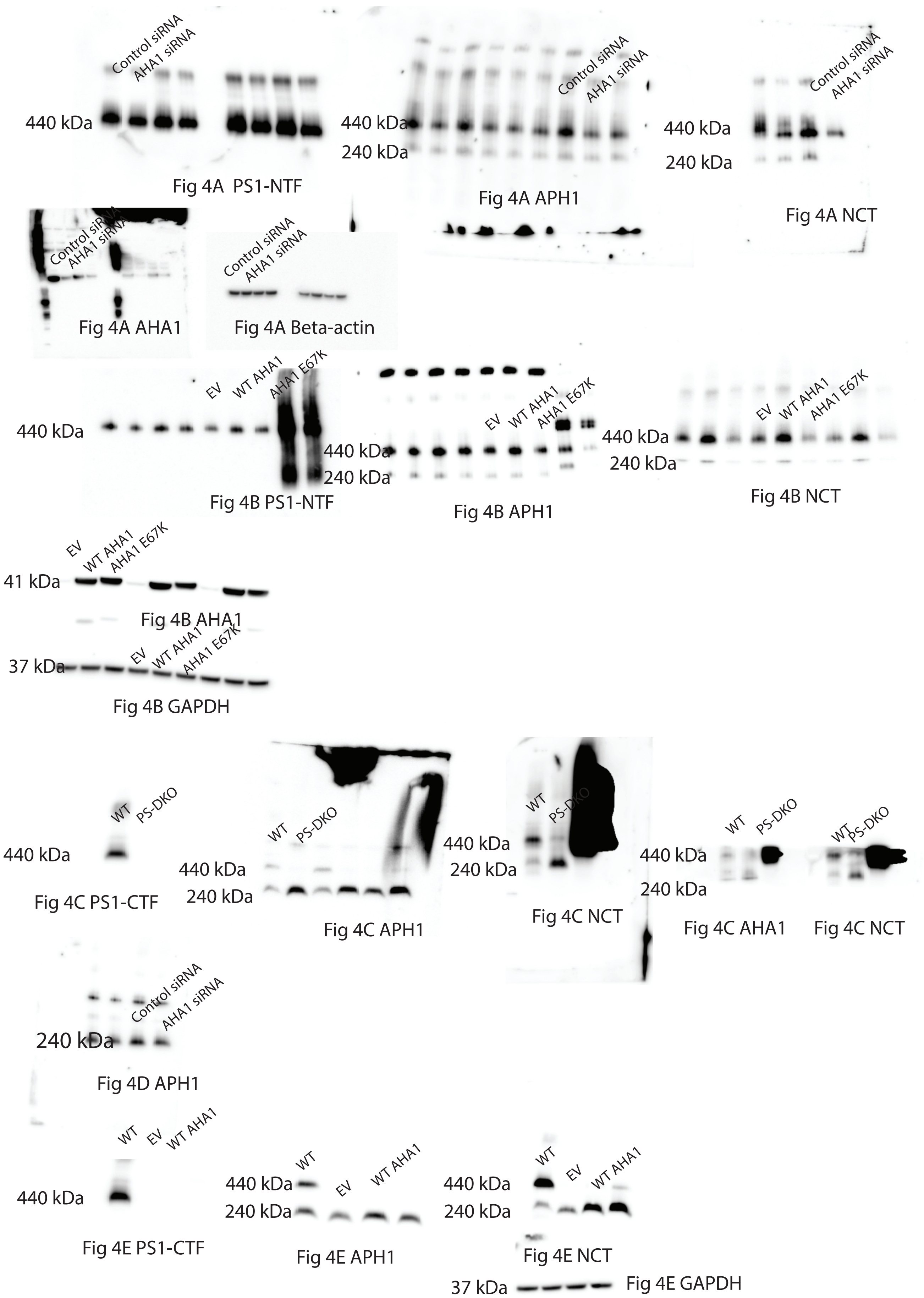
Control siRNA
AHA1 siRNA
440 kDa
Fig 4A PS1-NTF
440 kDa
240 kDa
Control siRNA
AHA1 siRNA
Fig 4A APH1
Control siRNA
AHA1 siRNA
440 kDa
240 kDa
Fig 4A NCT
Control siRNA
AHA1 siRNA
Fig 4A AHA1
Control siRNA
AHA1 siRNA
Fig 4A Beta-actin
EV
WT AHA1
AHA1 E67K
440 kDa
Fig 4B PS1-NTF
EV
WT AHA1
AHA1 E67K
440 kDa
240 kDa
Fig 4B APH1
EV
WT AHA1
AHA1 E67K
440 kDa
240 kDa
Fig 4B NCT
EV
WT AHA1
AHA1 E67K
41 kDa
Fig 4B AHA1
EV
WT AHA1
AHA1 E67K
37 kDa
Fig 4B GAPDH
WT
PS-DKO
440 kDa
Fig 4C PS1-CTF
WT
PS-DKO
440 kDa
240 kDa
Fig 4C APH1
WT
PS-DKO
440 kDa
240 kDa
Fig 4C NCT
WT
PS-DKO
440 kDa
240 kDa
Fig 4C AHA1
WT
PS-DKO
Fig 4C NCT
Control siRNA
AHA1 siRNA
240 kDa
Fig 4D APH1
WT
EV
WT AHA1
440 kDa
Fig 4E PS1-CTF
WT
EV
WT AHA1
440 kDa
240 kDa
Fig 4E APH1
WT
EV
WT AHA1
440 kDa
240 kDa
Fig 4E NCT
37 kDa
Fig 4E GAPDH

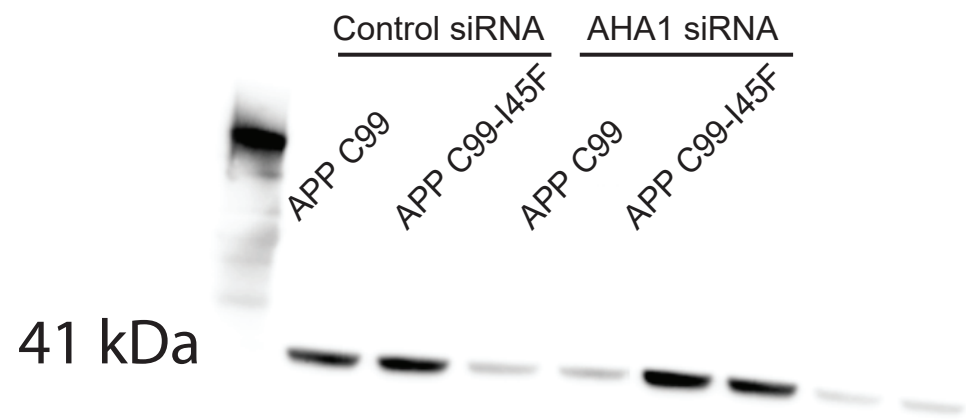

Fig 5C AHA1

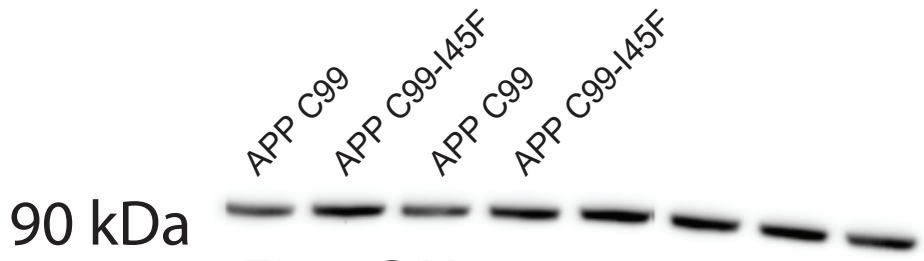

Fig 5C Hsp90

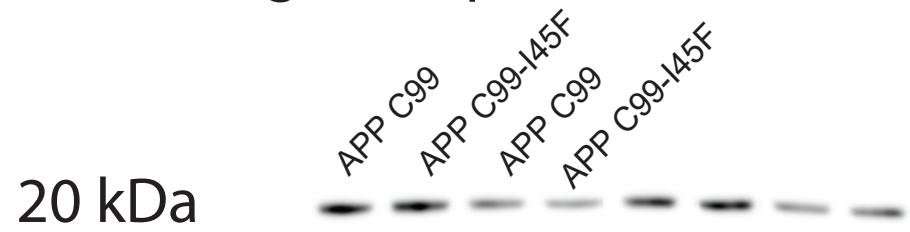

Fig 5C PS1-CTF

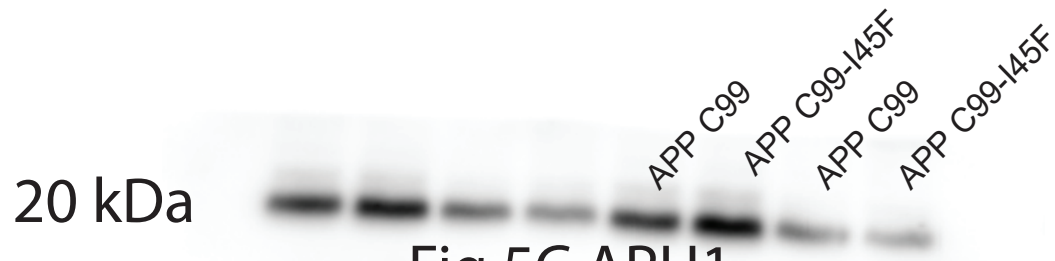

Fig 5C APH1

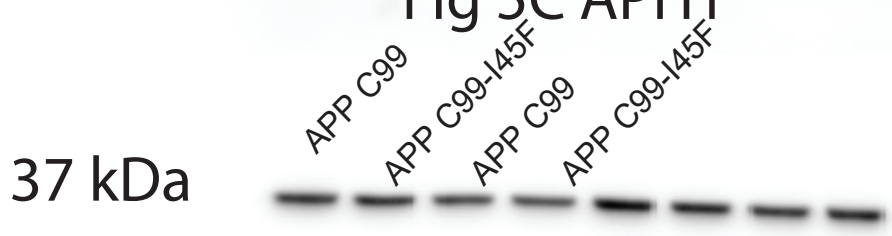

Fig 5C GAPDH

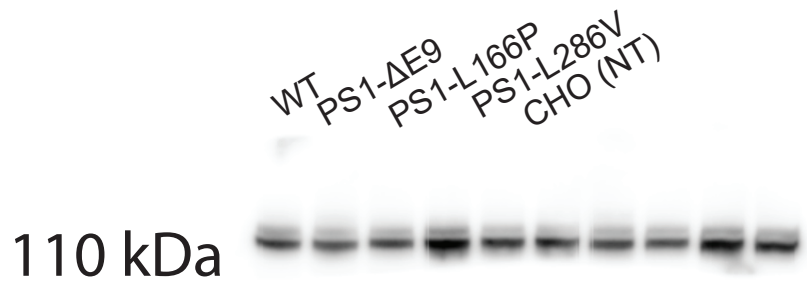

Fig 6A APP

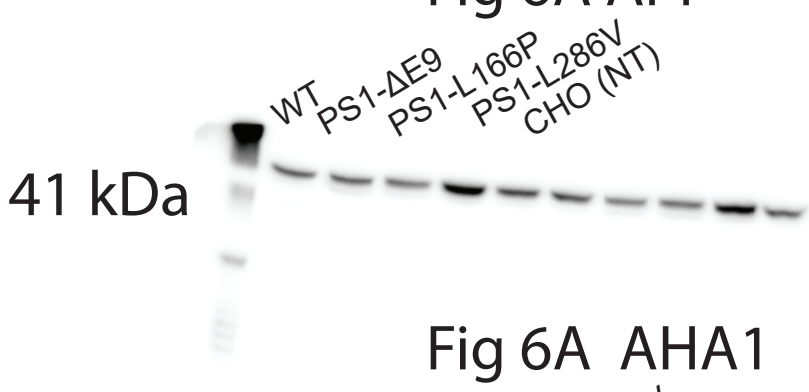

Fig 6A AHA1

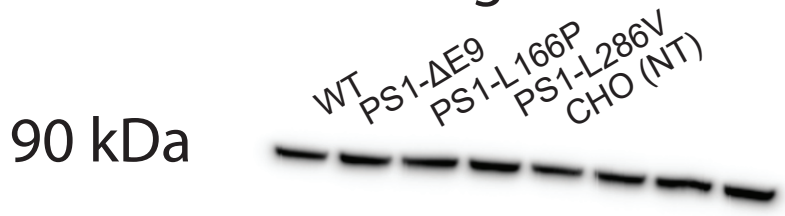

Fig 6 A Hsp90

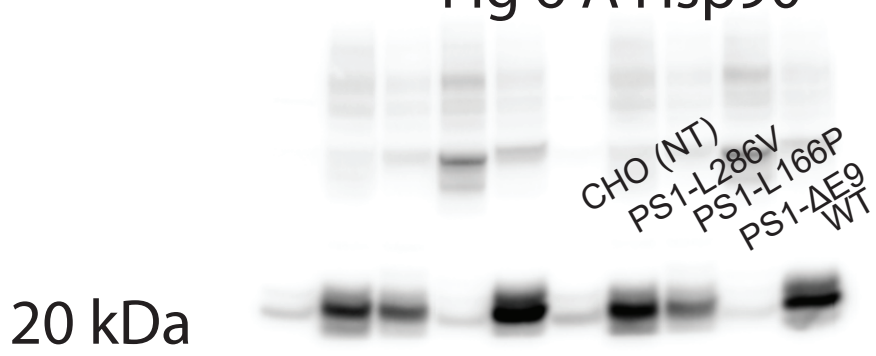

Fig 6A PS1-CTF

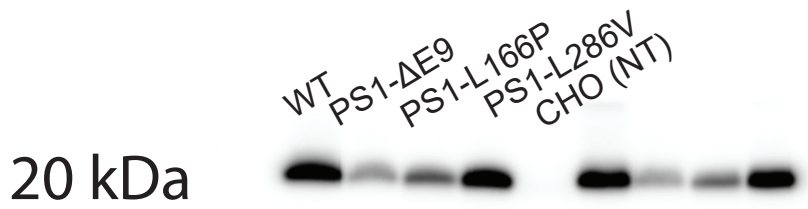

Fig 6A APH1

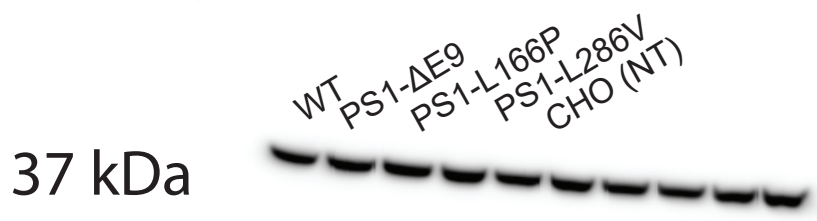

Fig 6A GAPDH

90 kDa

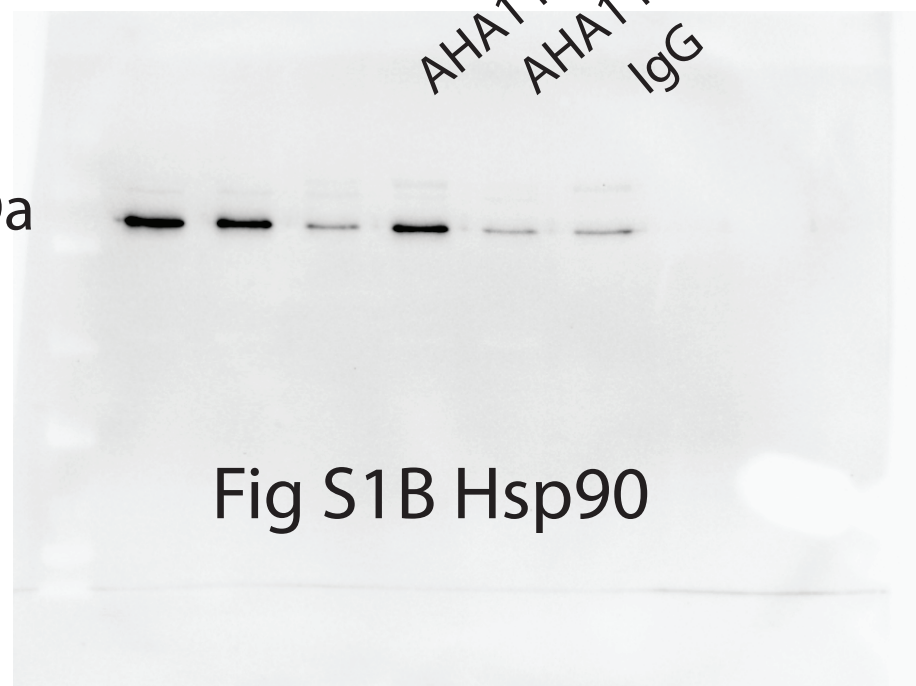

90 kDa

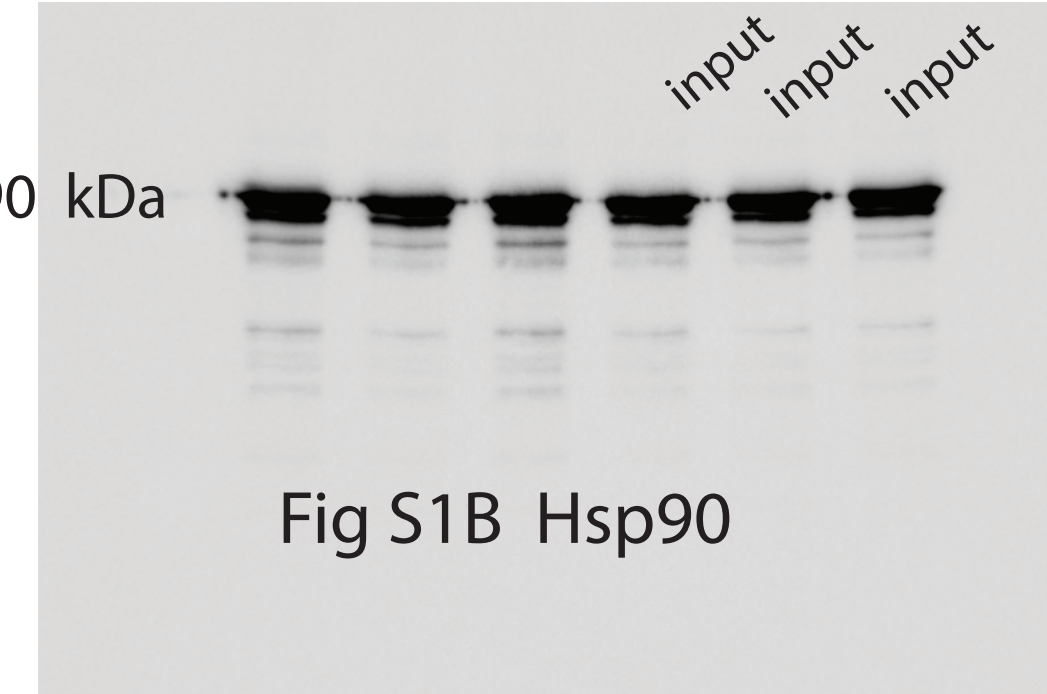

41 kDa

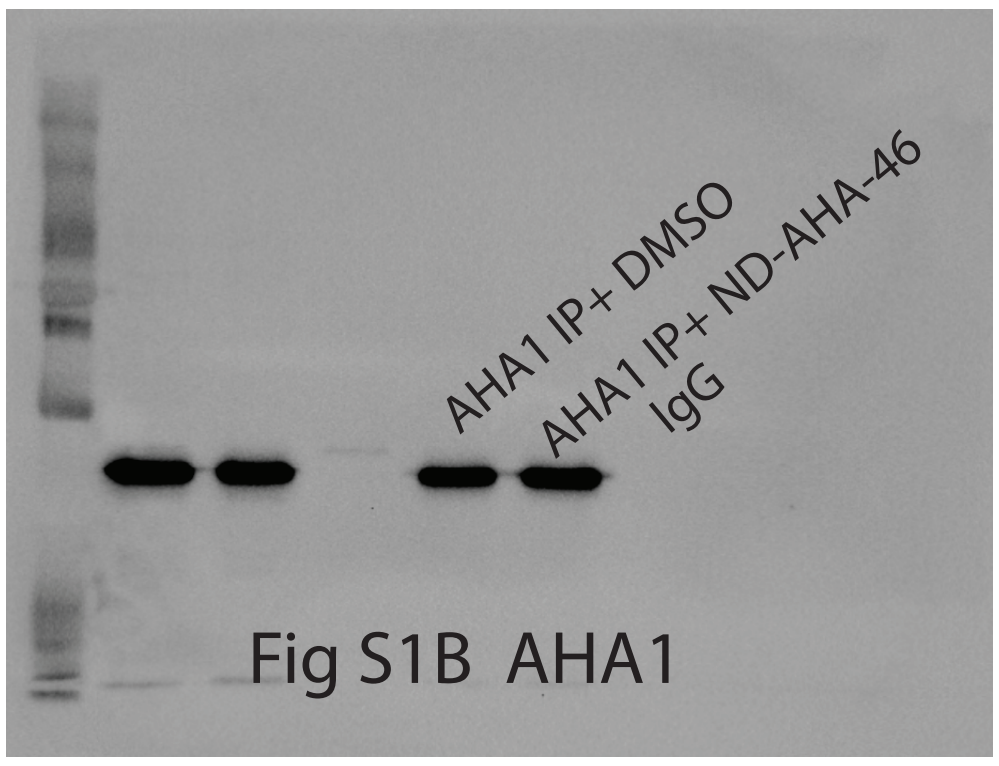

41 kDa

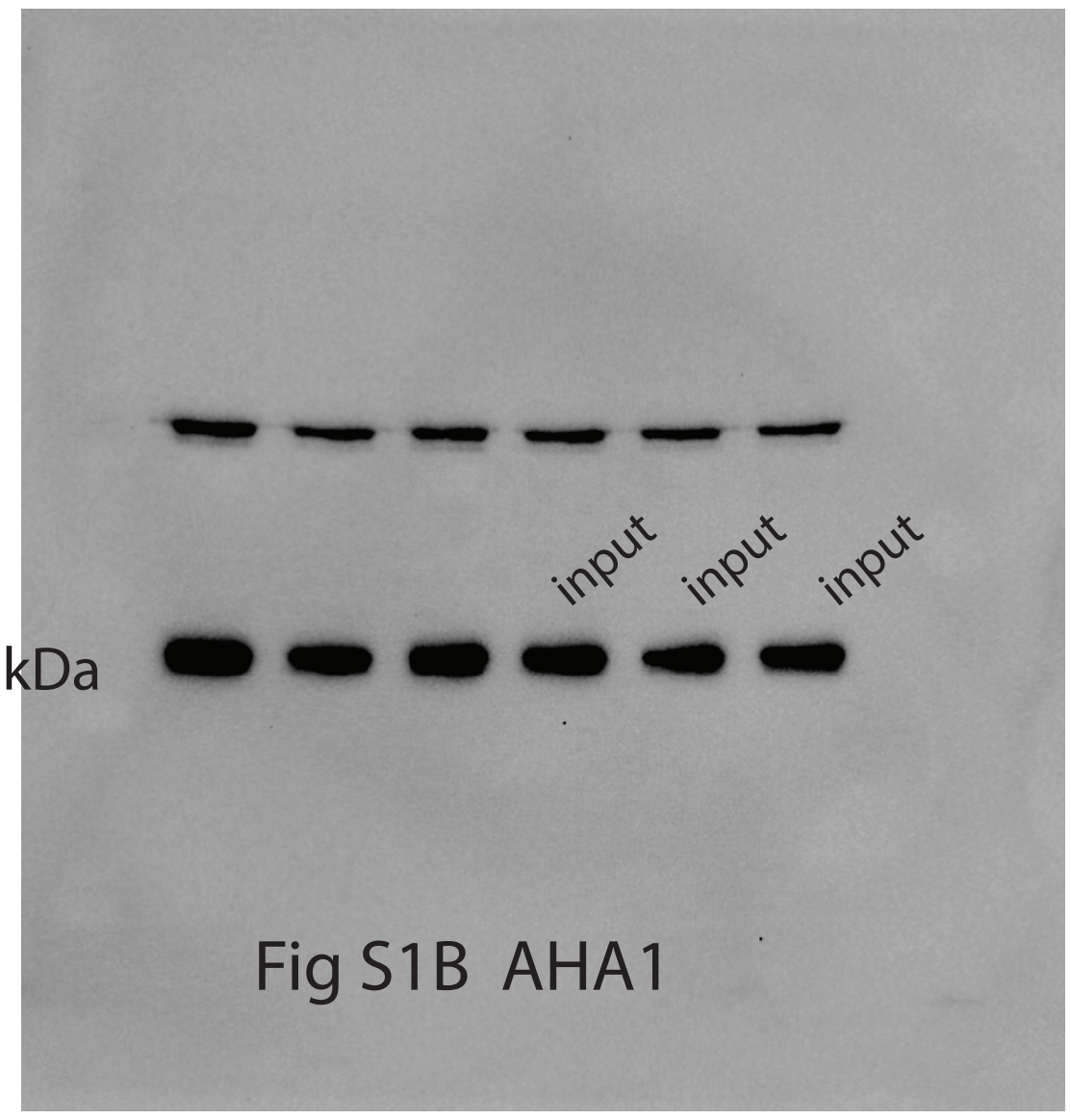

37 kDa

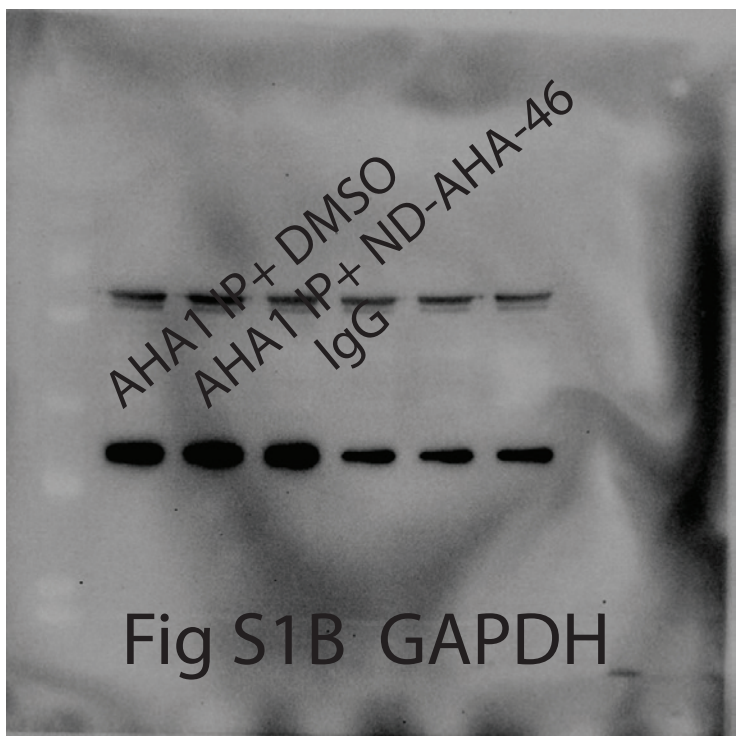

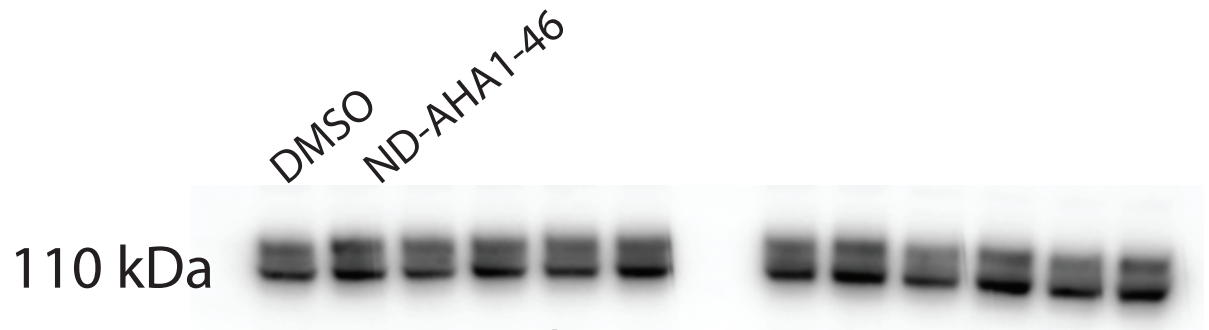

Fig S2A APP

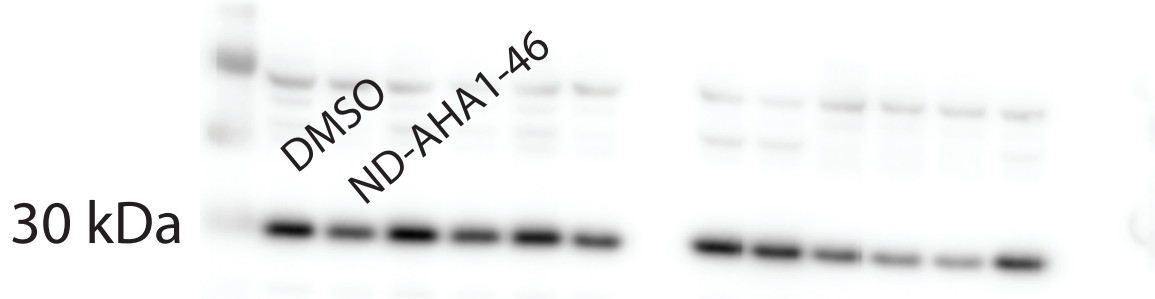

Fig S2A PS1-NTF

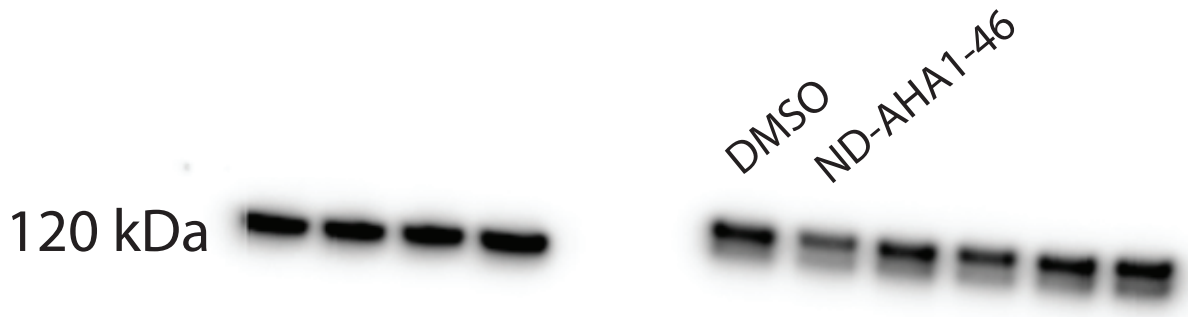

Fig S2A NCT

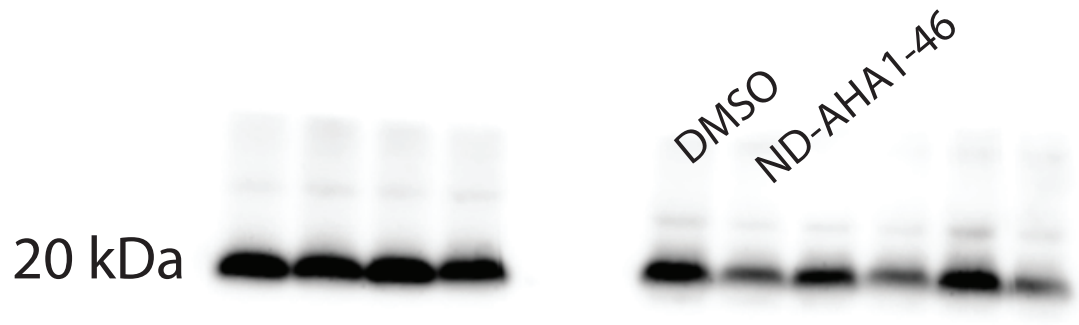

Fig S2A APH1

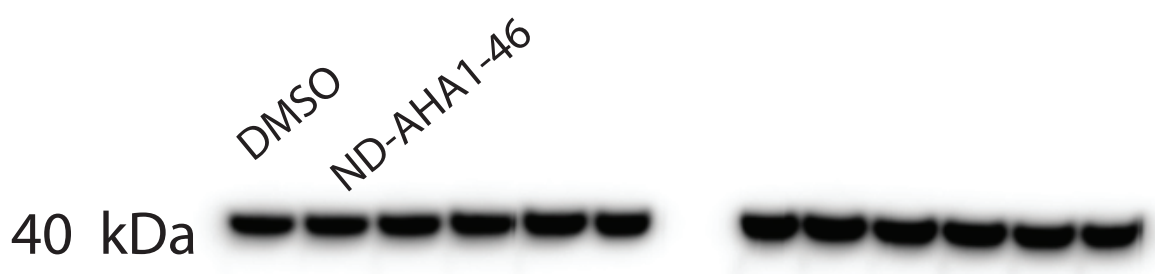

Fig S2A Beta-actin

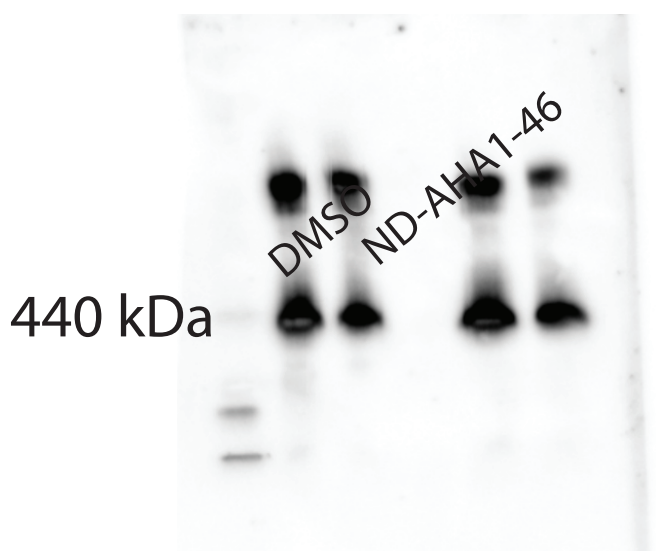

Fig S2B PS1-NTF

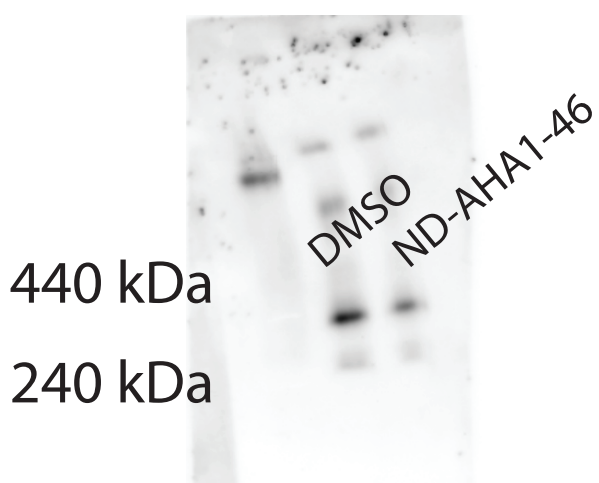

Fig S2B APH1

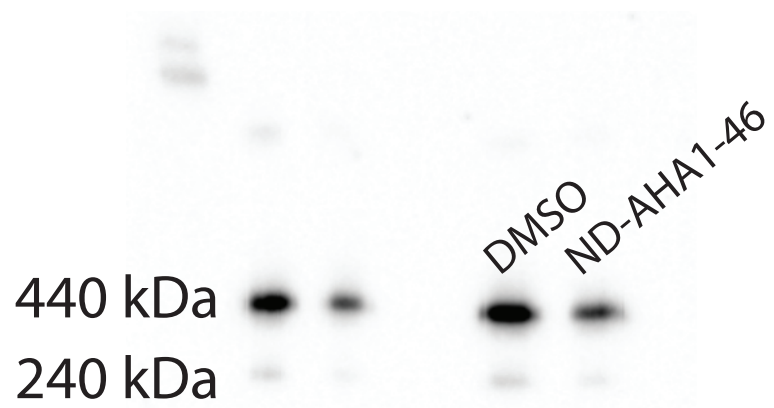

Fig S2B NCT

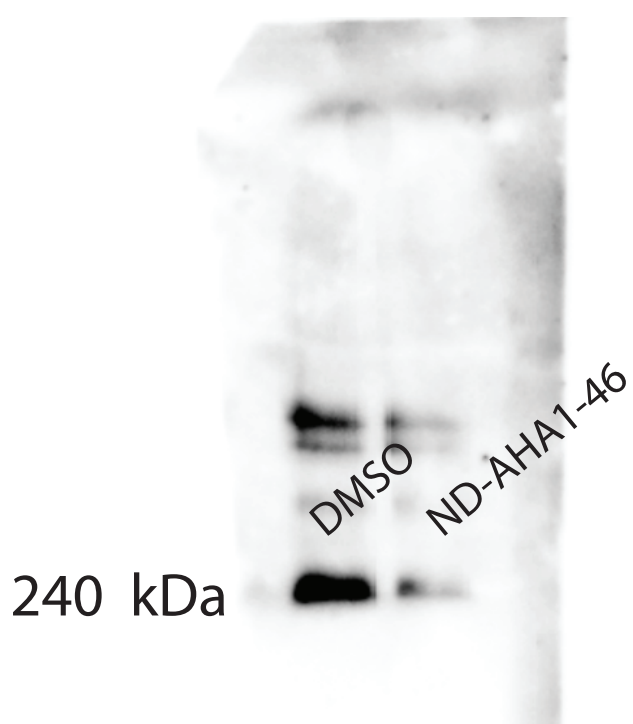

Fig S2C APH1

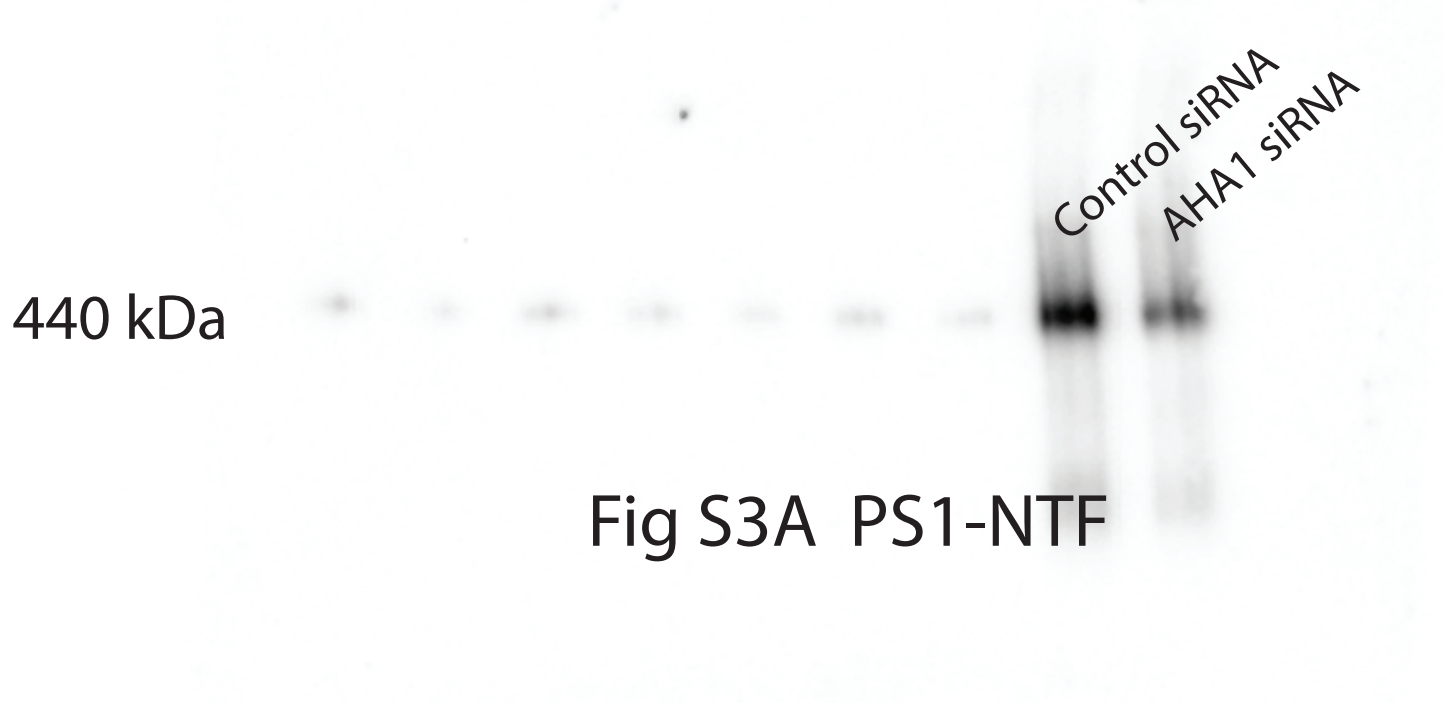

Fig S3A PS1-NTF

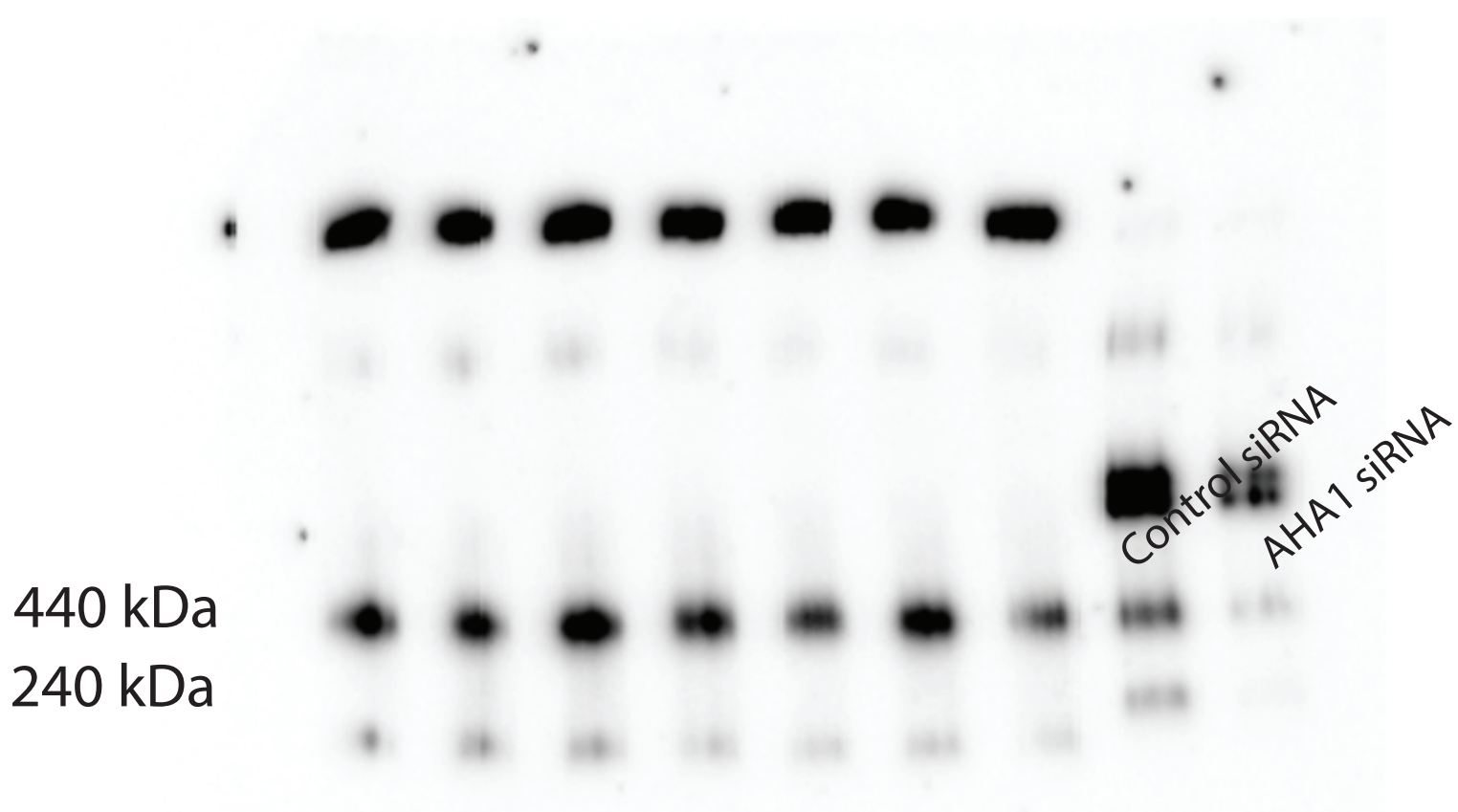

Fig S3A APH1

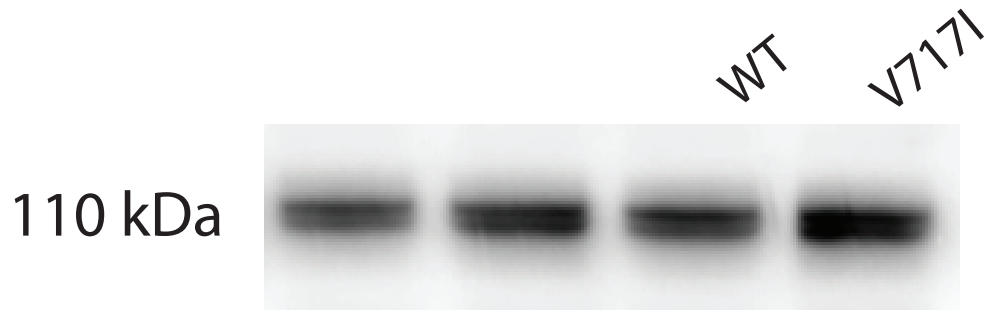

Fig S4B APP

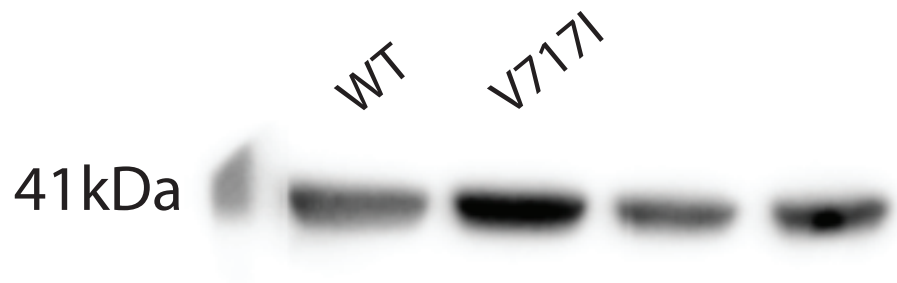

Fig S4B AHA1

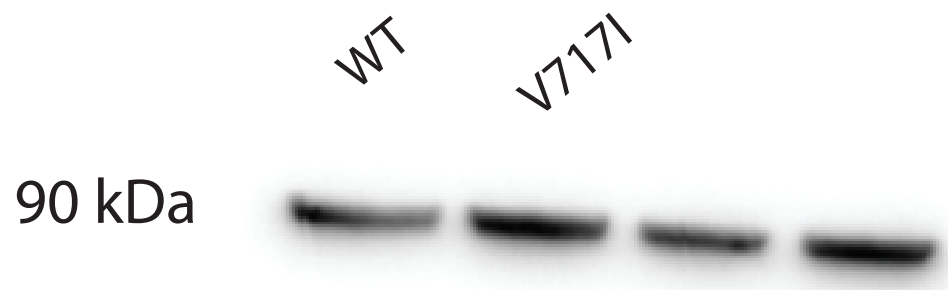

Fig S4B Hsp90

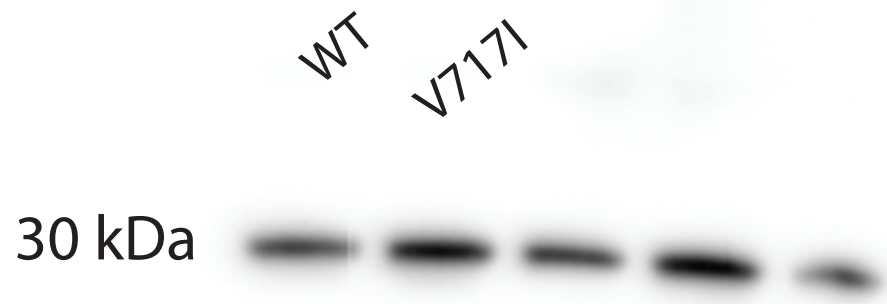

Fig S4B PS1-NTF

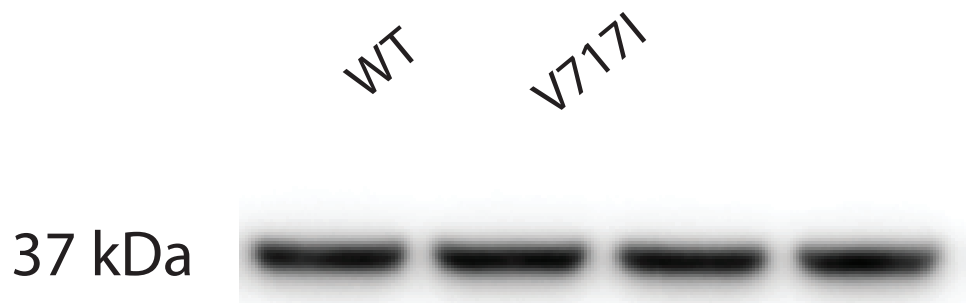

Fig S4B GAPDH

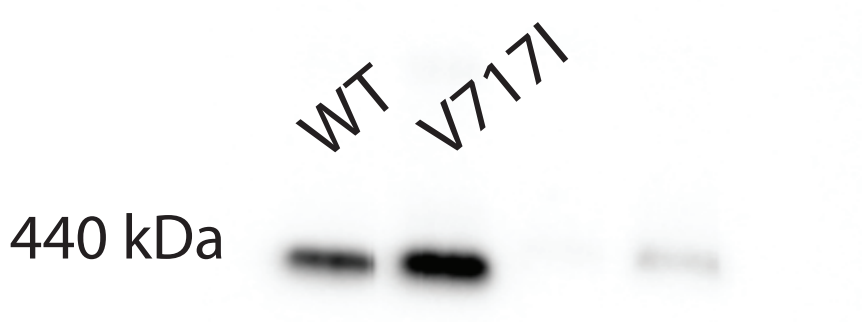

Fig S4C PS1-NTF

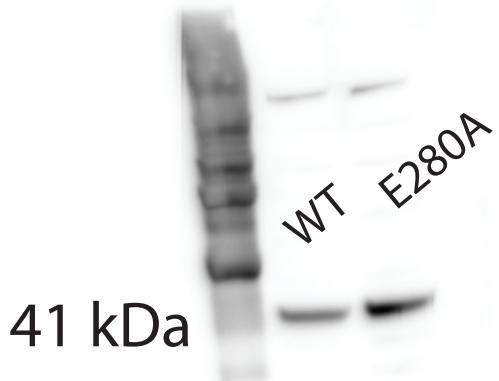

Fig S5A AHA1

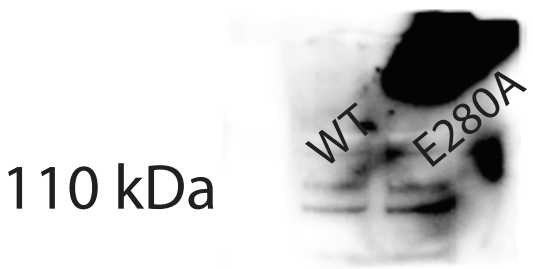

Fig S5A APP

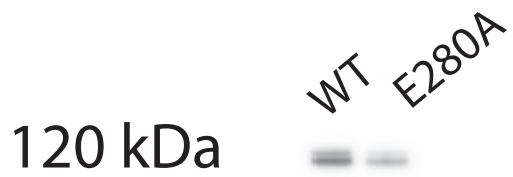

Fig S5A NCT

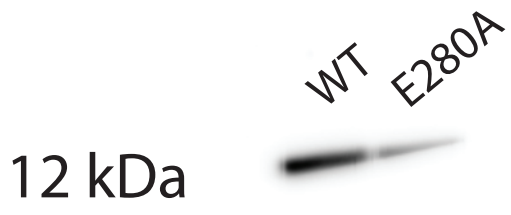

Fig S5A PEN2

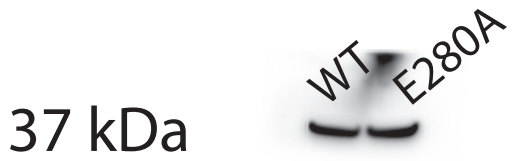

Fig S5A GAPDH

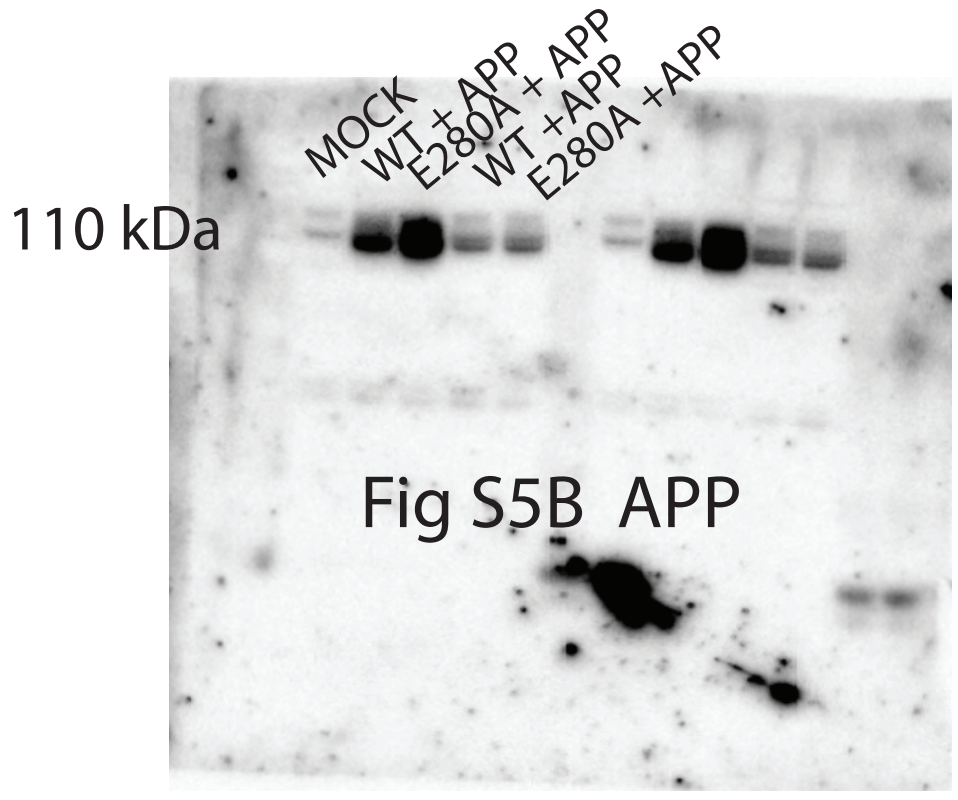

Fig S5B APP

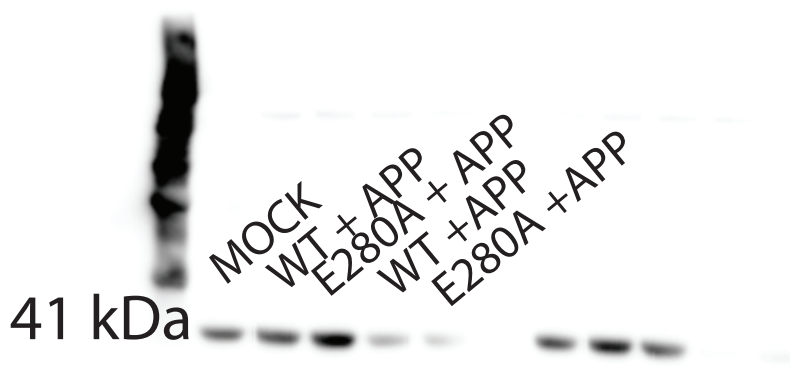

Fig S5B AHA1

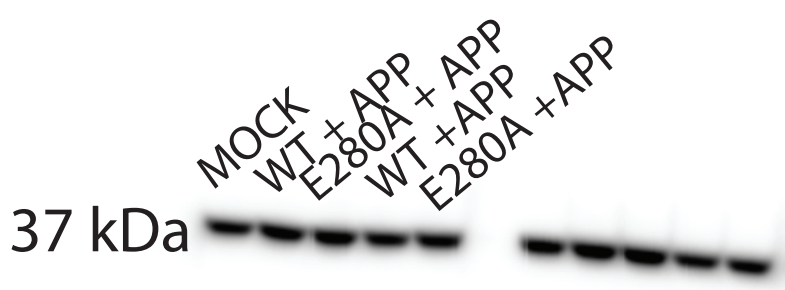

Fig S5B GAPDH

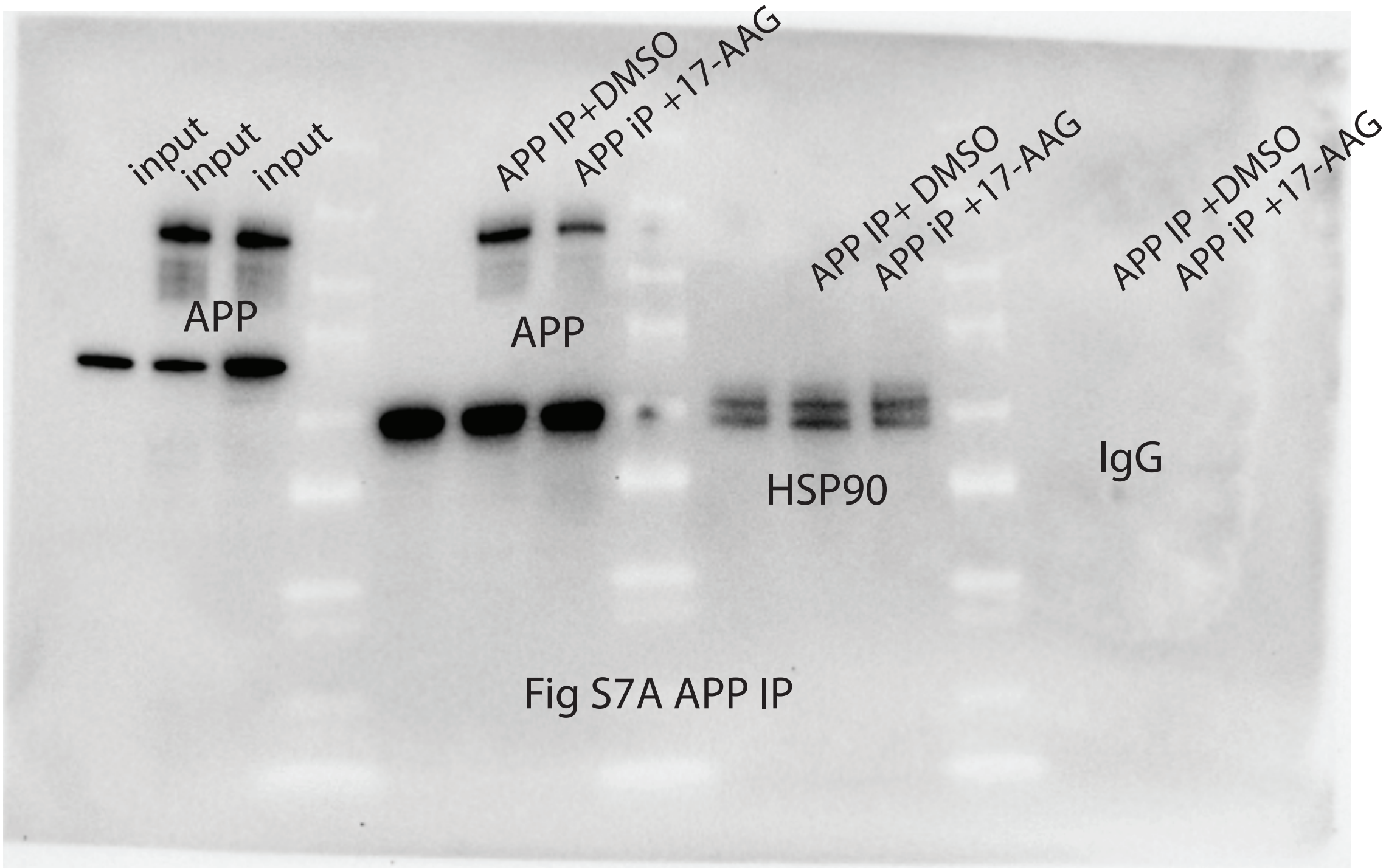
input
input
input
APP
APP IP+DMSO
APP iP +17-AAG
APP
APP IP+ DMSO
APP iP +17-AAG
HSP90
APP IP +DMSO
APP iP +17-AAG
IgG
Fig S7A APP IP

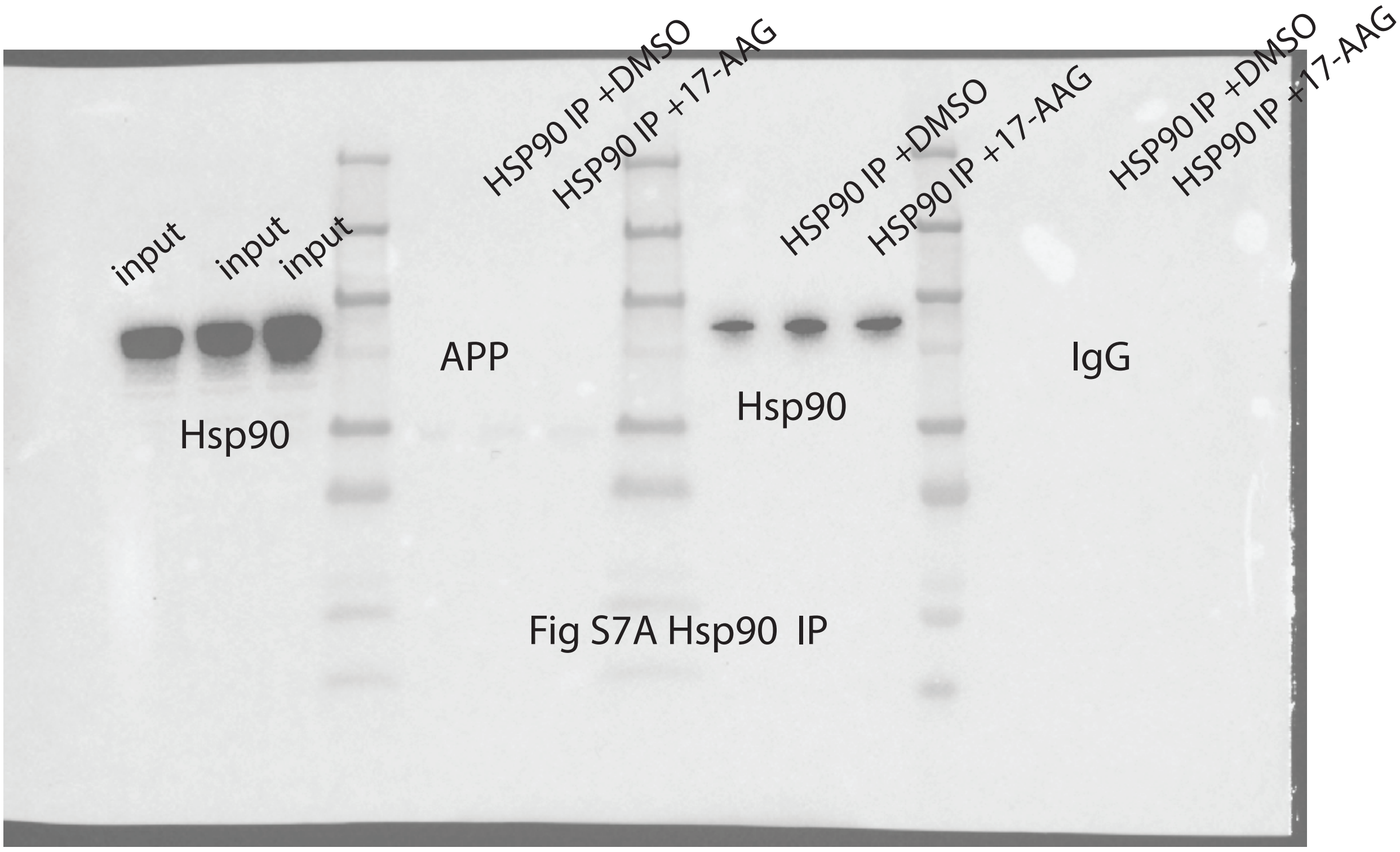
input
input
input
Hsp90
HSP90 IP +DMSO
HSP90 IP +17-AAG
APP
HSP90 IP +DMSO
HSP90 IP +17-AAG
Hsp90
HSP90 IP +DMSO
HSP90 IP +17-AAG
IgG
Fig S7A Hsp90 IP
