# Supplementary material for: AHA1 Regulates Aβ Production via Modulation of APP Abundance and γ‐Secretase Assembly
Source: J Neurochem. 2026 Aug 27;170(9):e70544. doi: 10.1111/jnc.70544 (PMC13522734; doi:10.1111/jnc.70544)

Noorani et. al.,
Fig. 1.

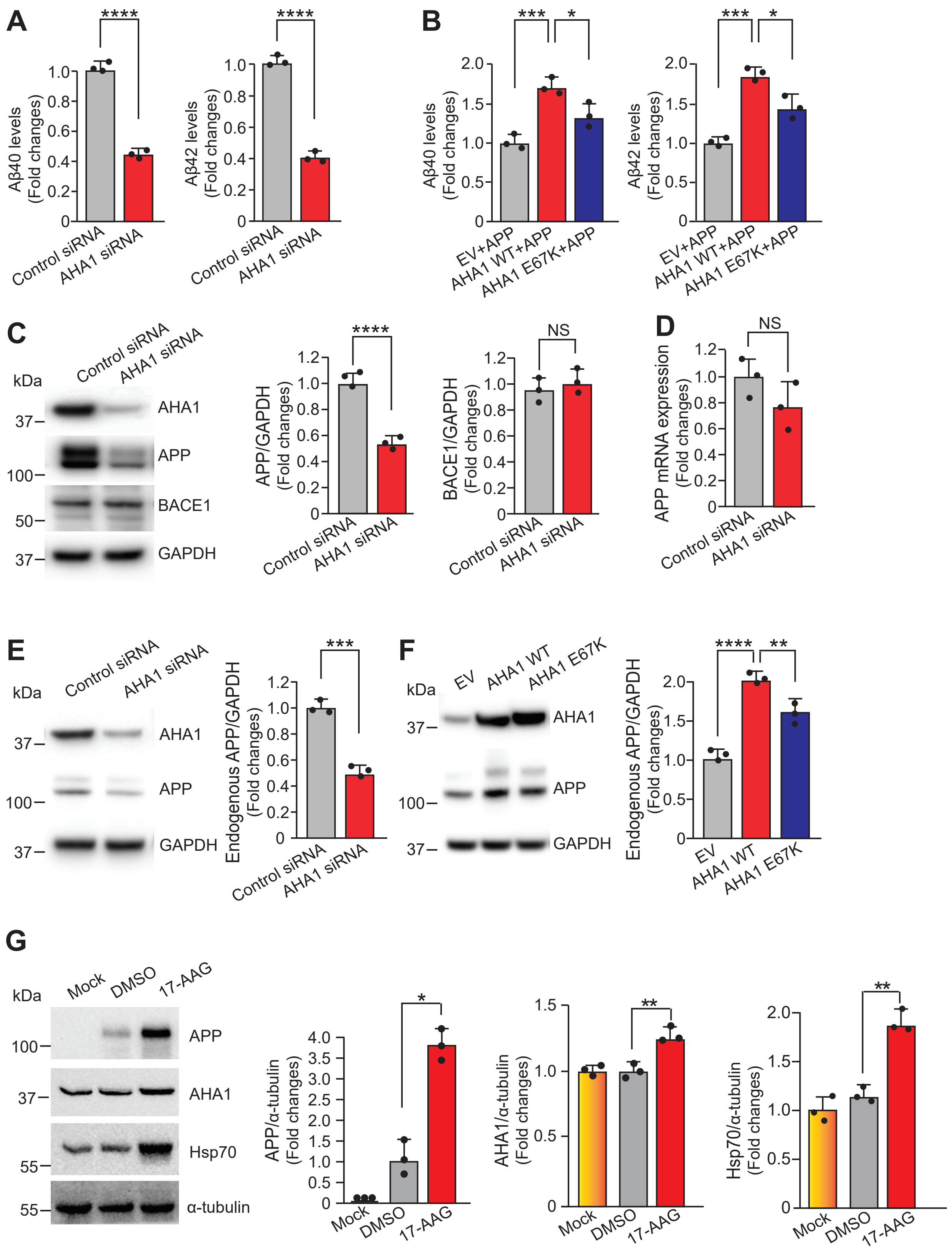

Noorani et. al.,
Fig. 2.

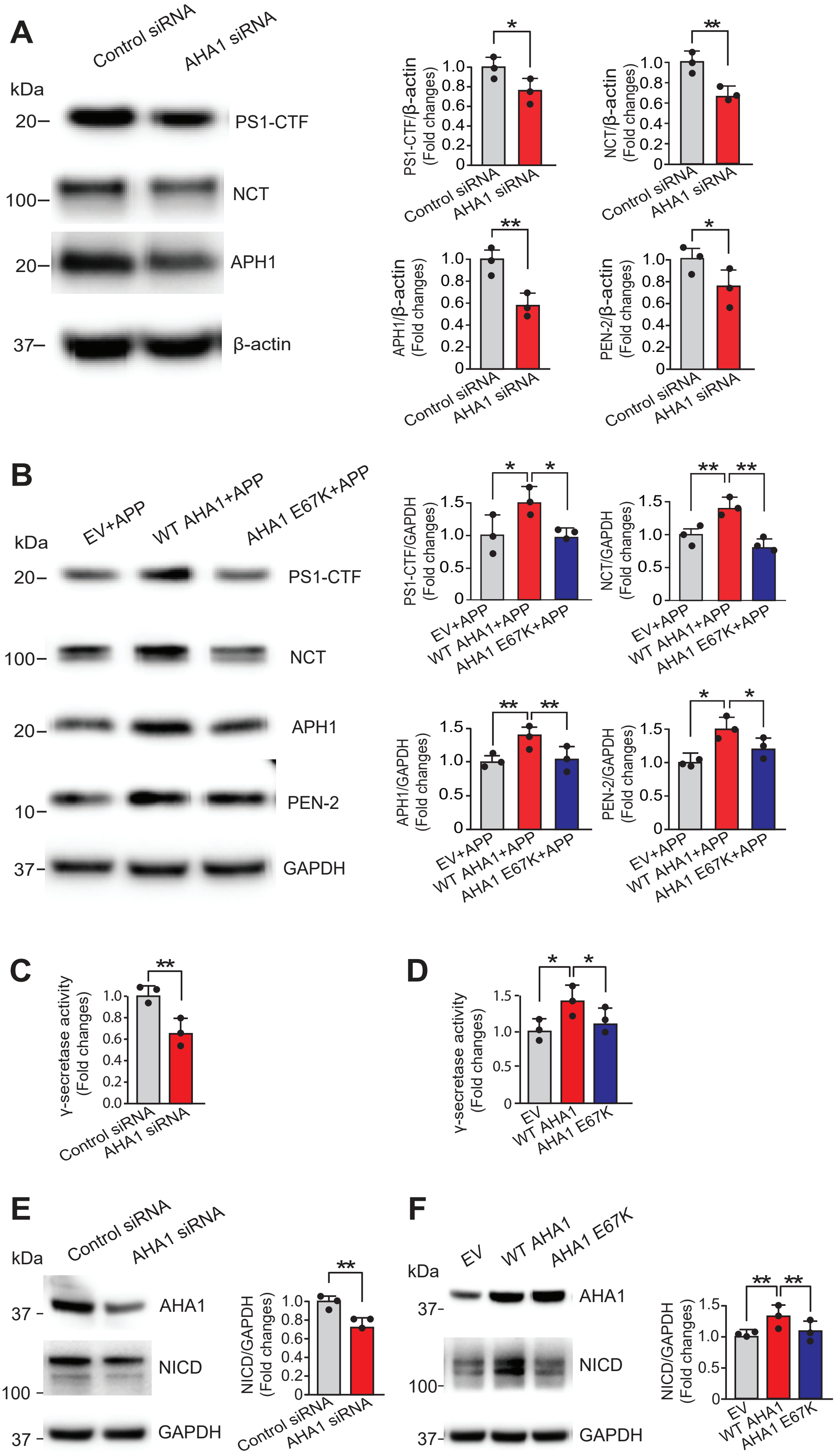

Noorani et. al.,
Fig. 3.

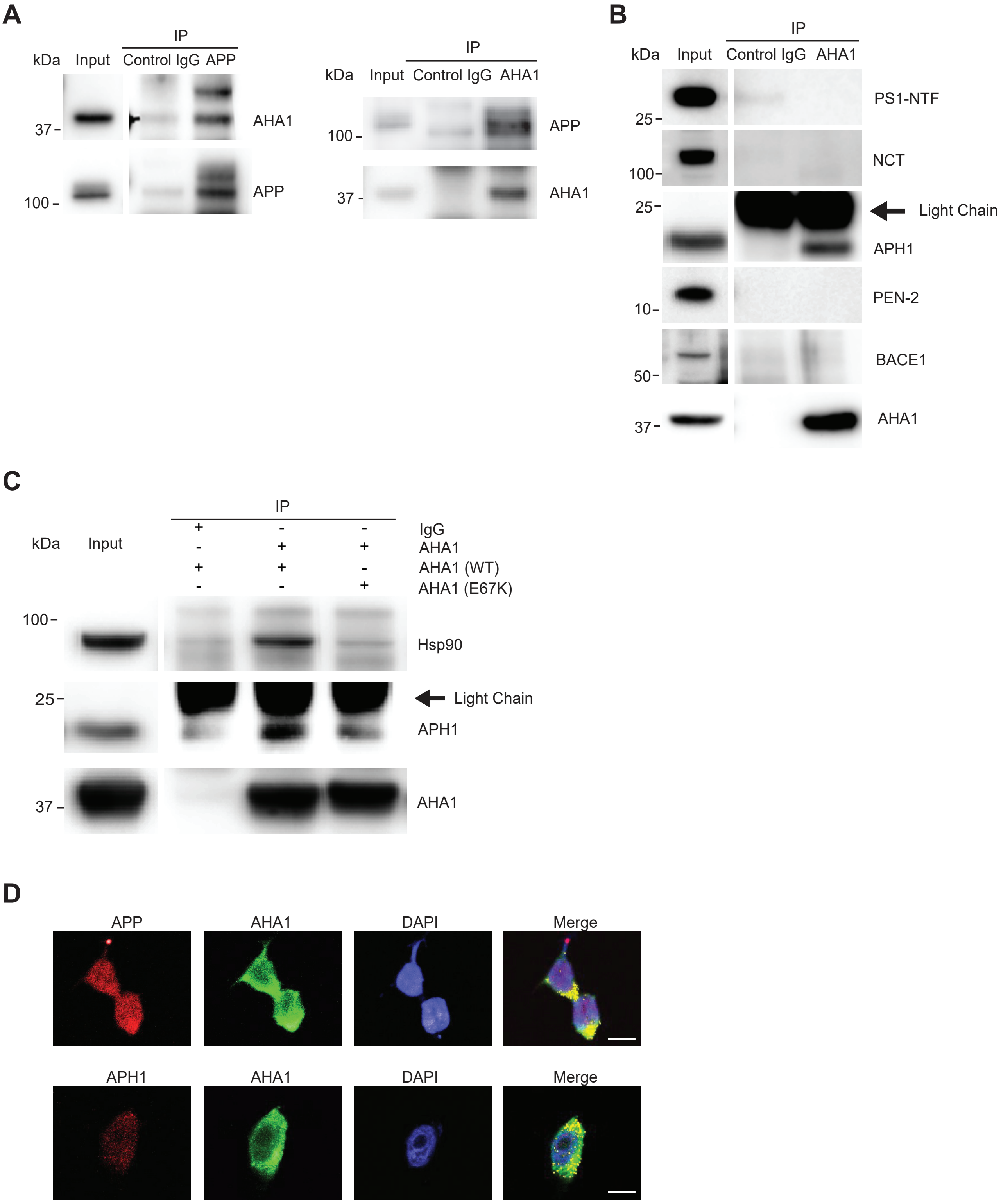

Noorani et. al.,
Fig. 4.

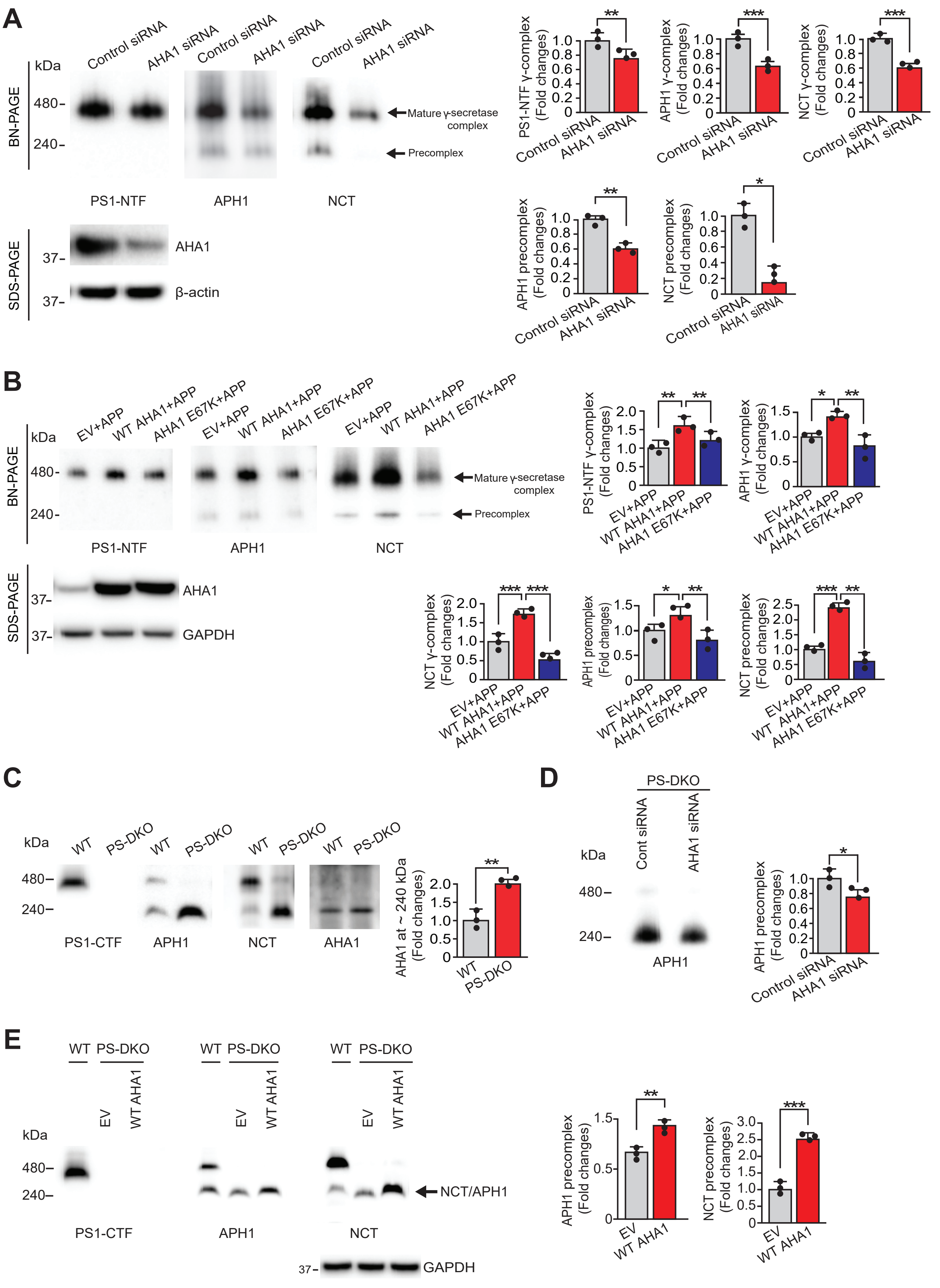

Noorani et. al.,
Fig. 5.

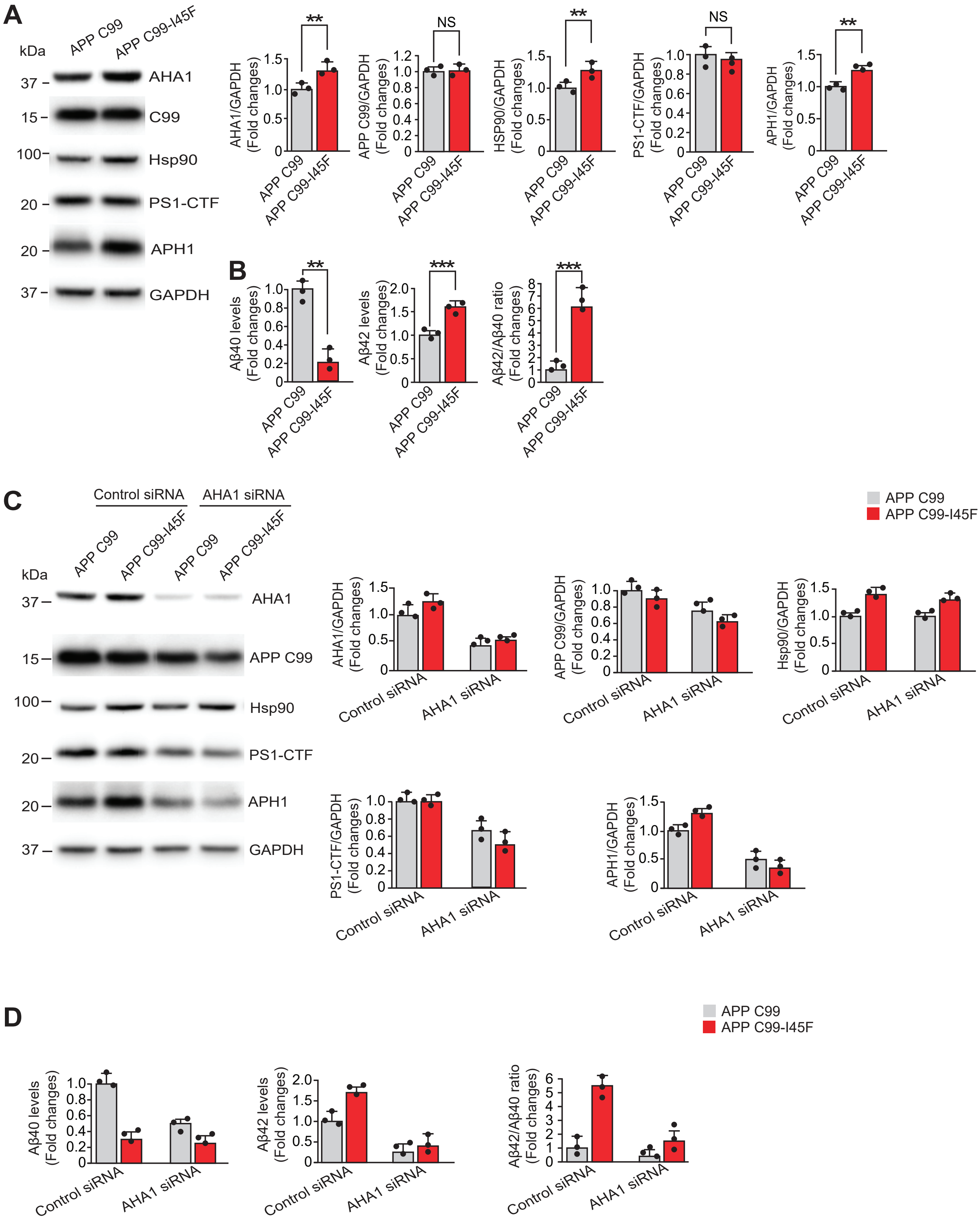

Noorani et. al.,
Fig. 6.

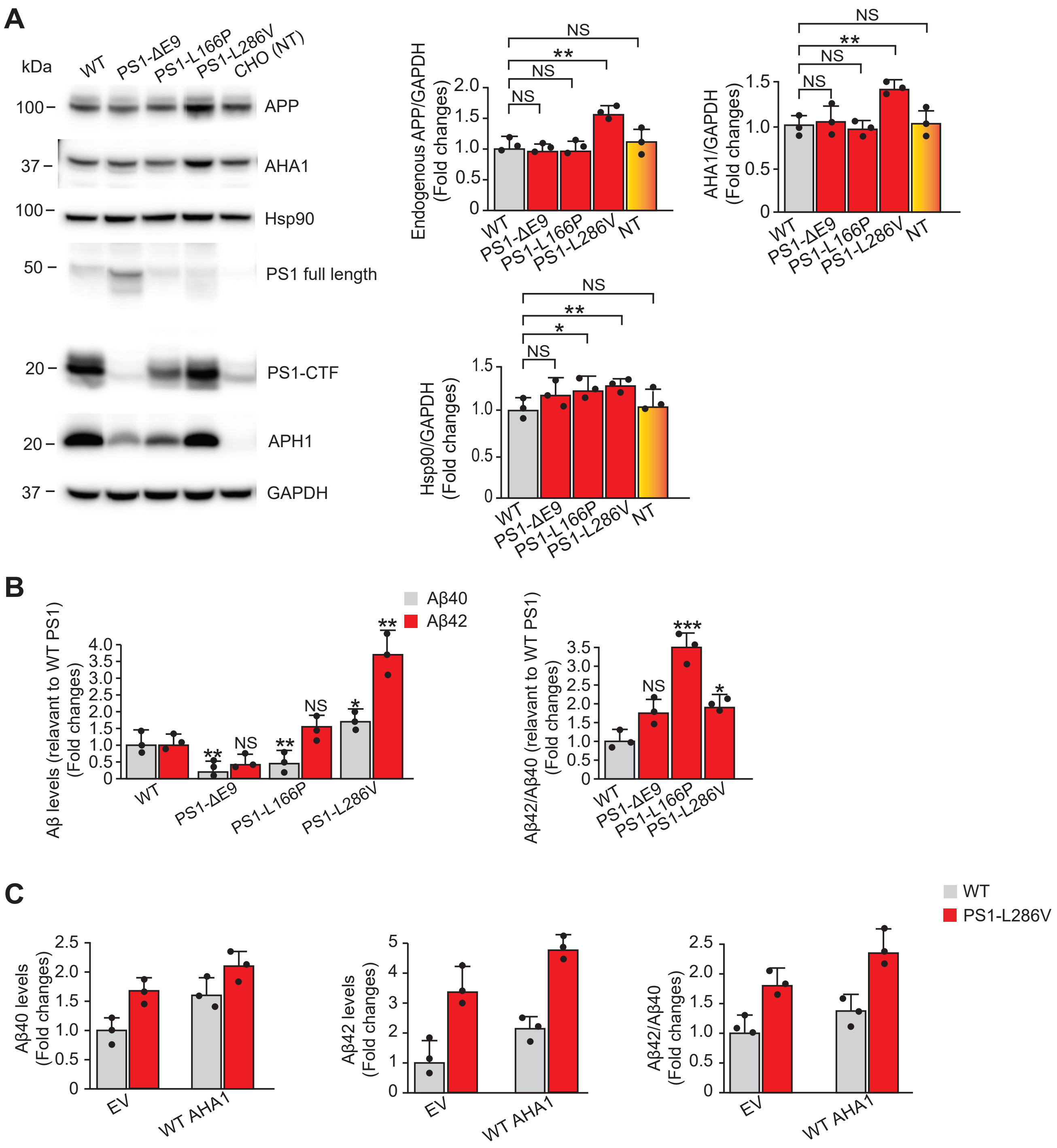

Supplement: Supplementary file 2 [file JNC-170-e70544-s002.zip › Noorani_et_al_FINAL_Main Figures_1–6(1).pdf]
